# p38MAPK guards the integrity of endosomal compartments through regulating necrotic death

**DOI:** 10.1038/s41598-022-20786-4

**Published:** 2022-09-29

**Authors:** Jia Yao, Svetlana Atasheva, Randall Toy, Emmeline L. Blanchard, Philip J. Santangelo, Krishnendu Roy, Edward S. Mocarski, Dmitry M. Shayakhmetov

**Affiliations:** 1 Departments of Pediatrics and Medicine, Lowance Center for Human Immunology, Emory University School of Medicine, Atlanta, GA 30322 USA; 2 Wallace H. Coulter Department of Biomedical Engineering, Georgia Institute of Technology and Emory University, Atlanta, GA 30332 USA; 3 Department of Microbiology and Immunology, Emory University School of Medicine, Atlanta, GA 30322 USA; 4 Emory Vaccine Center, Emory University School of Medicine, Atlanta, GA 30322 USA

**Keywords:** Immunology, Pathogenesis

## Abstract

Pathogens trigger activation of sensors of the innate immune system that initiate molecular signaling enabling appropriate host defense programs. Although recognition of pathogen-specific moieties or PAMPs by specialized receptors of the immune system is well defined for a great number of pathogens, the mechanisms of sensing of pathogen-induced functional perturbations to the host cell remain poorly understood. Here we show that the disruption of endosomal compartments in macrophages by a bacterium or fully synthetic nanoparticles activates stress-response p38MAPK kinase, which triggers execution of cell death of a necrotic type. p38MAPK-mediated necrosis occurs in cells with a compound homozygous deletion of pyroptosis-inducing caspases-1 and -11, apoptotic caspase-8, and necroptosis-inducing receptor-interacting protein kinase-3 (RIPK3), indicating that all of these principal cell death mediators are dispensable for p38MAPK-induced necrosis in response to endosome rupture. p38MAPK-mediated necrosis is suppressed by the receptor-interacting protein kinase 1, RIPK1, and degradation of RIPK1 sensitizes macrophages to necrotic death. Since pathogen-induced cell death of necrotic types is implicated in host defense against infection, our results indicate that functional perturbations in host cells are sensed as a component of the innate immune system.

## Introduction

Physiologically-regulated necrosis plays an essential role in development, tissue homeostasis, and host defense against bacterial and viral pathogens^1,2^. Based on genetic and biochemical evidence, several distinct pathways of regulated necrosis have been defined^3^. Among these, the two most thoroughly characterized are receptor-interacting protein kinase 3 (RIPK3)-dependent necroptosis^4,5^ and caspase-1- and/or caspase-11-dependent pyroptosis^6–9^. These have been implicated in the development of severe inflammation when deregulated or activated in response to pathogens^4,10^. The molecular signaling that underlies recognition of pathogens depends on sensors of an array of chemically-distinct pathogen-associated molecular patterns (PAMPs) that represents a complexity of microbes and viruses regularly encountered by the host^11^. Using specific knockdown of multiple components of signaling pathways mediating cell death, it was recently shown that macrophages can execute cell death in a functionally-redundant manner by flexibly engaging distinct cell death mechanisms, which leads to host protection against lethal Salmonella infection^12^. Although sensing of influenza virus infection via TLR3- and RIG-I-dependent RNA sensing pathways is well established^13,14^, recent data indicate that ZBP1-dependent sensing of IAV and viral ribonucleoprotein complexes leads to execution of necrotic cell death^15–18^. It was shown that IAV-dependent activation of ZBP1 leads to the assembly of a cell death-inducing complex, called the PANaptosome, which contemporaneously activates pyroptotic, apoptotic, and necroptotic cell death pathways, and ZBP1-dependent PANaptosome activation is critical for host protection from IAV^19,20^. In addition to specific recognition, infection perturbs and stresses host cells, for example via pathogen-activated “outside-in” signaling during endocytosis and pathogen-induced endosome rupture^21^. Although PAMP recognition by cellular receptors has been studied in great detail, the pathways and the consequences of functional perturbation of the cell by the pathogen-induced endosome rupture remains poorly understood. Here we show that the rupture of macrophage endosomal compartments by a cytosolic bacterium or synthetic nanoparticles results in activation of p38MAPK signaling, leading to execution of a novel necrotic form of cell death. We further show that p38MAPK-dependent necrosis occurs independently of all known principal components of pyroptotic, apoptotic, or necroptotic cell death pathways as macrophages with compound homozygous deletion of Caspases-1, -8 and -11, as well as RIPK3 are still able to undergo necrotic death in p38MAPK-dependent manner. We further show that a scaffold activity of RIPK1 counteracts p38MAPK-dependent necrosis and pharmacologic or genetic knockdown of RIPK1 sensitizes macrophages to necrotic death in response to endosome rupture. Taken together, our data suggest a unique pathogen sensing mechanism through endosomal compartment instability that relies on MAPK signaling in response to cytosolic entry of intracellular pathogens.

## Results

### *L.monocytogenes* or synthetic nanoparticles trigger necrotic death of Mϕs independently of caspases-1, -8, -11 and RIPK3

**Figure 1:**
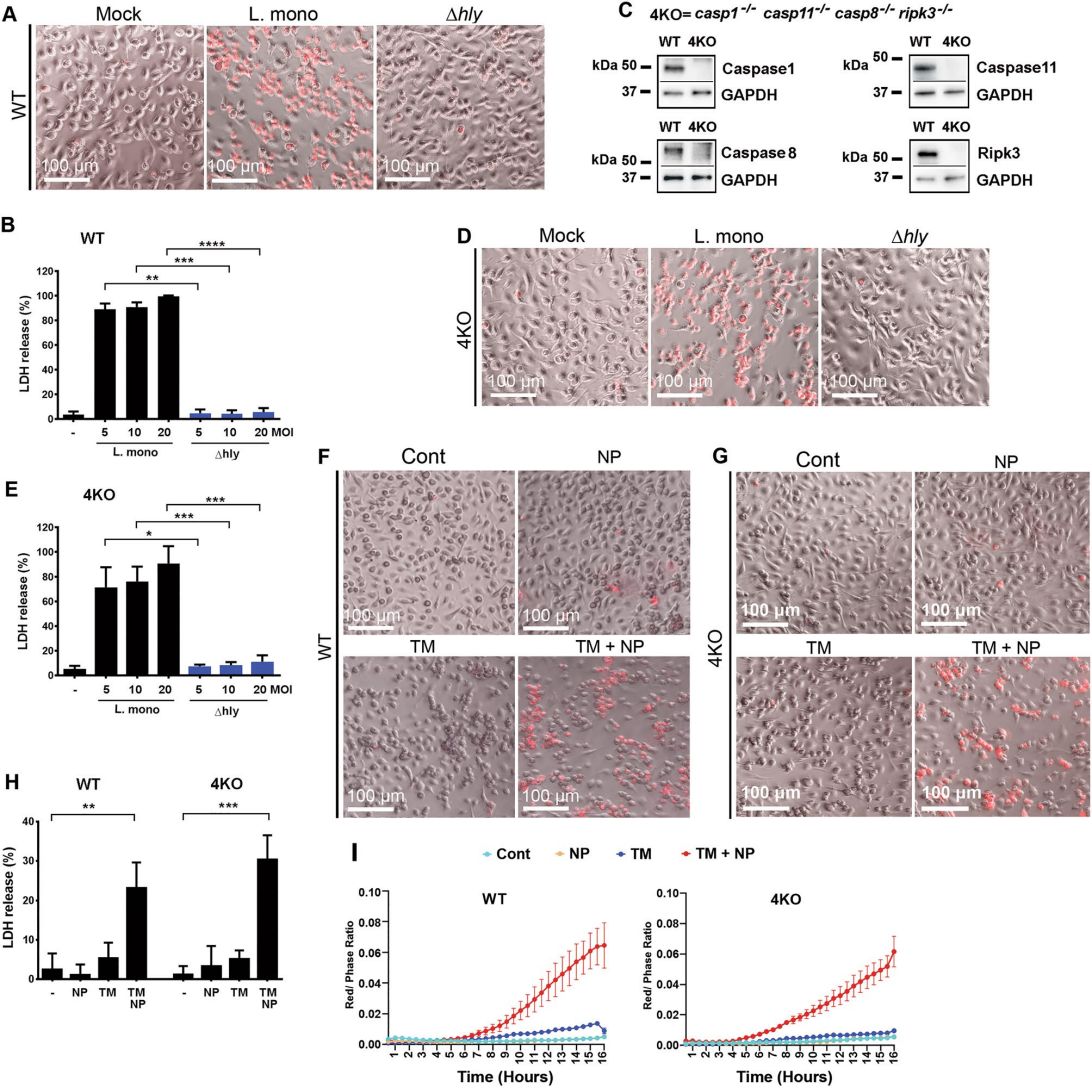
*L.monocytogenes* or synthetic nanoparticles trigger necrotic death of Mϕs from *WT* mice and mice with compound homozygous deficiency in caspases-1, -8, -11, and RIPK3. **(A)** BMDMs from WT mice undergo necrosis after infection with *L. monocytogenes* (MOI 5) as determined by the addition of PI (red) into the culture medium. Its isogenic strain (Δ*hly*), unable to penetrate endosomal compartment, does not cause necrotic death in BMDM. Images of representative fields acquired using bright-field and red channels are shown. Scale bar is 100 μm. N = 3. **(B)** Lactate dehydrogenase (LDH) release from BMDMs after infection with wild-type *L. monocytogenes* (L. mono) or its isogenic strain (Δ*hly*) at indicated MOIs. Data are presented as Mean ± SD, N = 3. **(C)** Western blot analysis of expression of Caspases-1, -8, -11, and RIPK3 in WT and compound homozygous deficient mice (4KO). GAPDH–glyceraldehyde-3-phosphate dehydrogenase. N = 5. **(D)** BMDMs from 4KO mice undergo necrosis after infection with *L. monocytogenes* (MOI 5), while isogenic strain (Δ*hly*) does not cause necrotic death in 4KO BMDM. Images of representative fields acquired using bright-field and red channels are shown. Scale bar is 100 μm. N = 3. **(E)** LDH release from 4KO BMDMs after infection with wild-type *L. monocytogenes* (L. mono) or its isogenic strain at indicated MOIs. N = 3. **(F)** BMDM from WT mice undergo necrosis after exposure to TM and NP as determined by the addition of PI (red) into the culture medium. Images of representative fields of Mϕs treated with DMSO (Cont), TM, NP, or with TM and NP combination, acquired using bright-field and red channels are shown. Scale bar is 100 μm. N = 20. **(G)** BMDM from 4KO mice undergo necrosis after exposure to TM and NP as determined by the addition of PI (red) into the culture medium. Images of representative fields of Mϕs treated with DMSO (Cont), TM, NP, or with TM and NP combination, acquired using bright-field and red channels are shown. Scale bar is 100 μm. N = 20. **(H)** LDH release from WT or 4KO BMDM after their treatment with NP, tunicamycin (TM), and their combination. N = 4. **(I)** Kinetics of necrotic death induction in WT and 4KO BMDM after their exposure to indicated stimuli and determined by IncuCyte continuous image acquisition. Necrotic cell death is assessed as ratio of red object confluence divided by phase object confluence and is depicted on the y-axis. Representative data are shown. N = 3.

To establish a tractable system to investigate functional consequences of pathogen phagocytosis and entry into the cytosol, we differentiated bone marrow-derived macrophages (BMDM) from the WT mice in vitro and infected them with a prototypic cytosolic bacterium *L. monocytogenes*. In agreement with previous reports^22,23^, after infection with *L. monocytogenes*, BMDM underwent a necrotic form of death associated with the loss of plasma membrane integrity and release of cytosolic enzyme lactate dehydrogenase into the surrounding milieu (Fig. 1A and B). The isogenic strain of *L. monocytogenes,* Δ*hly*, that does not rupture endosomes^24^ (Supplemental Fig. S1 online), failed to trigger necrotic death of cultured BMDM (Fig. 1A and B). To define the pathway(s) of necrotic death triggered by *L. monocytogenes*, we differentiated BMDM from mice, which were quadruple-homozygous knockouts for caspases-1, -8, -11, and RIPK3 (*Casp1*^-/-^*Casp8*^-/-^*Casp11*^-/-^*Ripk3*^-/-^ or 4KO, Fig. 1C) and infected these cells with *L. monocytogenes*. Surprisingly, 4KO BMDM remained as susceptible to *L. monocytogenes* and underwent necrotic death similarly to WT controls (Fig. 1D and E).

To determine whether the abundance of specific bacterial PAMPs contributed to the induction of BMDM necrosis, we next compared cell responses to PAMP-dense *L.monocytogenes* or its isogenic Δ*hly* strain with cell responses to low-PAMP fully synthetic, chitosan-based, biodegradable nanoparticles (NPs) that rupture endosomes after internalization into the cell (Supplemental Fig. S2 online). While chitosan-based NPs are known to trigger complement activation and pro-inflammatory cytokine secretion in vivo^25^, treatment of BMDMs with chitosan-based NP in vitro triggers only very limited number of innate immune signaling pathways, compared to *L.monocytogenes* and Δ*hly* strain (Supplemental Figs. S3, S4 online). We serendipitously found that NPs trigger necrotic-type death of WT BMDM after conditioning of cells with a low dose of endoplasmic reticulum stressor tunicamycin (TM) that was unable to induce cell death alone (Fig. 1F and H). Similar to our findings with *L. monocytogenes*, BMDM from 4KO mice underwent necrotic death after treatment with NPs following cell conditioning with TM (Fig. 1G and H). Analysis of kinetics of necrosis induction showed that TM-treated BMDM first lost plasma membrane integrity at 6 h post NP addition and more cells continue progressively to die until the terminal time point of 16 h for both WT and 4KO BMDM (Fig. 1I).

**Figure 2:**
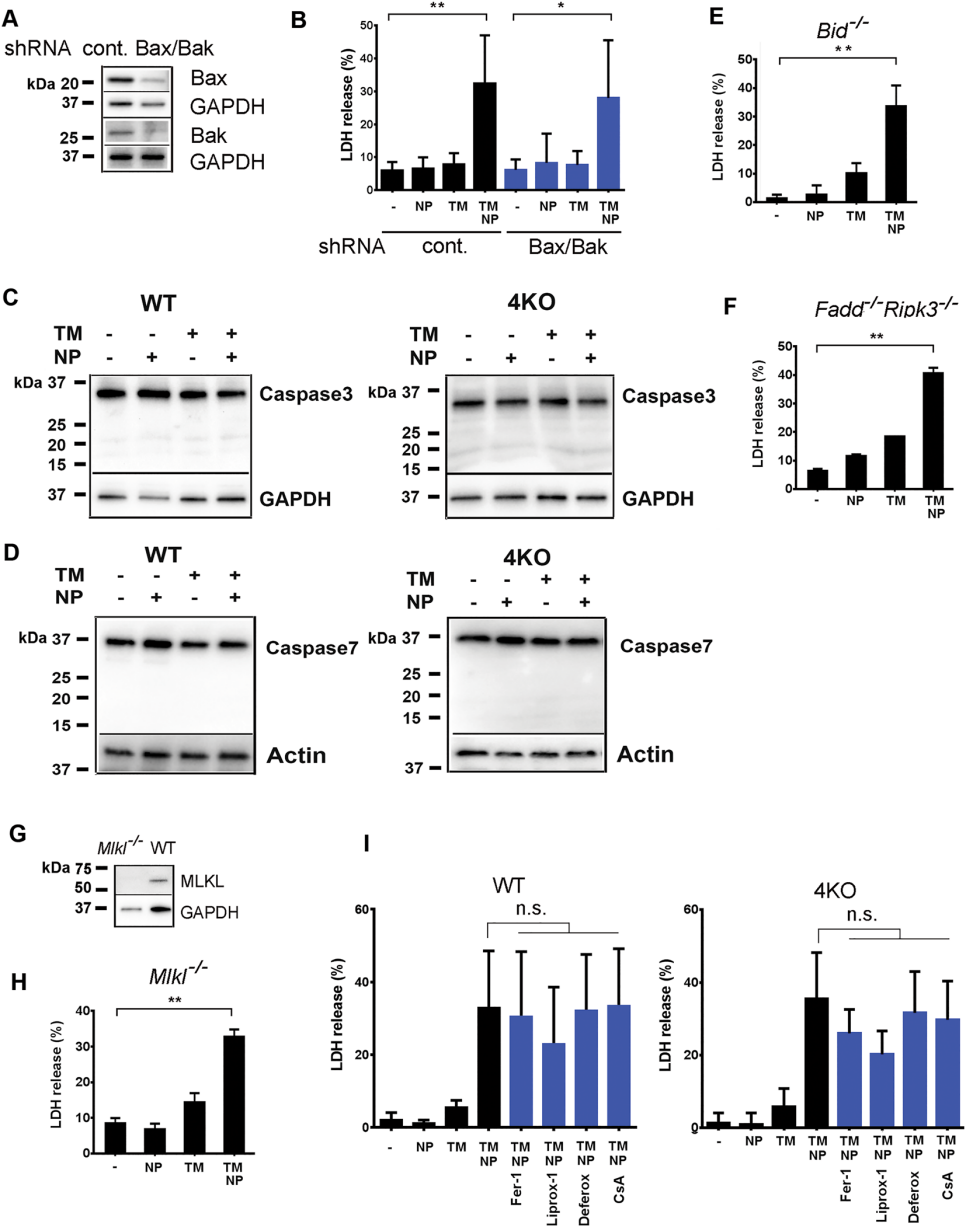
Synthetic nanoparticles trigger death of Mϕs in vitro independently of key regulators of cell death. **(A)** and **(B)**—BAX and BAK expression were knocked down by lentiviral shRNA stable-transduction in BMDM from 4KO mice. ShRNA knockdown efficacy was determined by Western Blotting **(A)** and BMDM death upon TM and nanoparticle treatment was determined by LDH release in the culture media **(B)** N = 5. **P = 0.0043; *P = 0.0258. **(C)** Caspase 3 activation in WT and 4KO BMDM was analyzed after TM and NP treatment by Western blotting. N = 4–5. **(D)** Caspase 7 activation in WT and 4KO BMDM was analyzed after TM and NP treatment by Western blotting. N = 2–3. **(E)** BMDM death from Bid^-/-^ mice were assessed by LDH activity release in the culture media, N = 3. **P = 0.0017 **(F)** BMDM death from Fadd^-/-^Ripk3^-/-^ mice was determined by LDH activity release in the culture media, N = 2, **P = 0.0018. **(G)** Western Blotting analysis shows lack of MLKL expression in Mlkl^-/-^ mice. N = 2. **(H)** BMDM death from Mlkl^-/-^ mice was assessed by LDH activity in the culture media, N = 2; *P* = 0.0055. **(I)** Effects of chemical inhibitors of known necrotic death pathways on necrotic death of WT and 4KO BMDM after TM and NP treatment were analyzed by measuring LDH activity in the culture media. N = 3. n.s.–not statistically significant.

To further explore cell death pathway response to endosome rupture, we analyzed responses of BMDM from mice deficient in key components of apoptotic signaling, necroptosis executioner MLKL, or in the presence of pharmacological inhibitors of ferroptosis or necrosis dependent of CYPD^1,3^. shRNA knockdown of both BAK and BAX in 4KO BMDM using shRNAs failed to protect from the NP-induced death (Fig. 2A and B). Apoptotic caspases -3 and -7 were not processed at the time of cell death in TM-treated WT and 4KO BMDM after their exposure to NP (Fig. 2C and D). BMDM from *Bid*^-/-^, *Fadd*^-/-^*Ripk3*^-/-^ (Fig. 2E and F), and *Mlkl*^-/-^ mice (Fig. 2G, H, and movie-S1 and movie-S2) were all susceptible to NP-induced necrosis. We used pharmacological inhibitors of other forms of necrotic death, namely ferroptosis and CYPD-dependent necrosis^3^ and found that these compounds were unable to significantly prevent death of WT or 4KO BMDM after their exposure to NPs (Fig. 2I). The genetic background of mice had no effect on BMDM death and Mϕs derived from WT mice of C57BL6/J or 129S1 strains underwent necrosis to a similar extent after their exposure to *L. monocytogenes* or NP (Supplemental Fig. S5 online).

Taken together, our data suggest that macrophages sense functional perturbations in a form of endosome rupture. Both the high-PAMP density pathogen *L. monocytogenes* and low-PAMP synthetic NPs that can rupture endosomes, activate necrotic-type death independently of components of pyroptotic (caspases-1 and -11), apoptotic (caspase-3, caspase-7, caspase-8, FADD, BID, BAK, and BAX), and necroptotic (RIPK3 and MLKL) cell death pathways.

### Synthetic nanoparticles trigger activation of MAPK signaling

**Figure 3:**
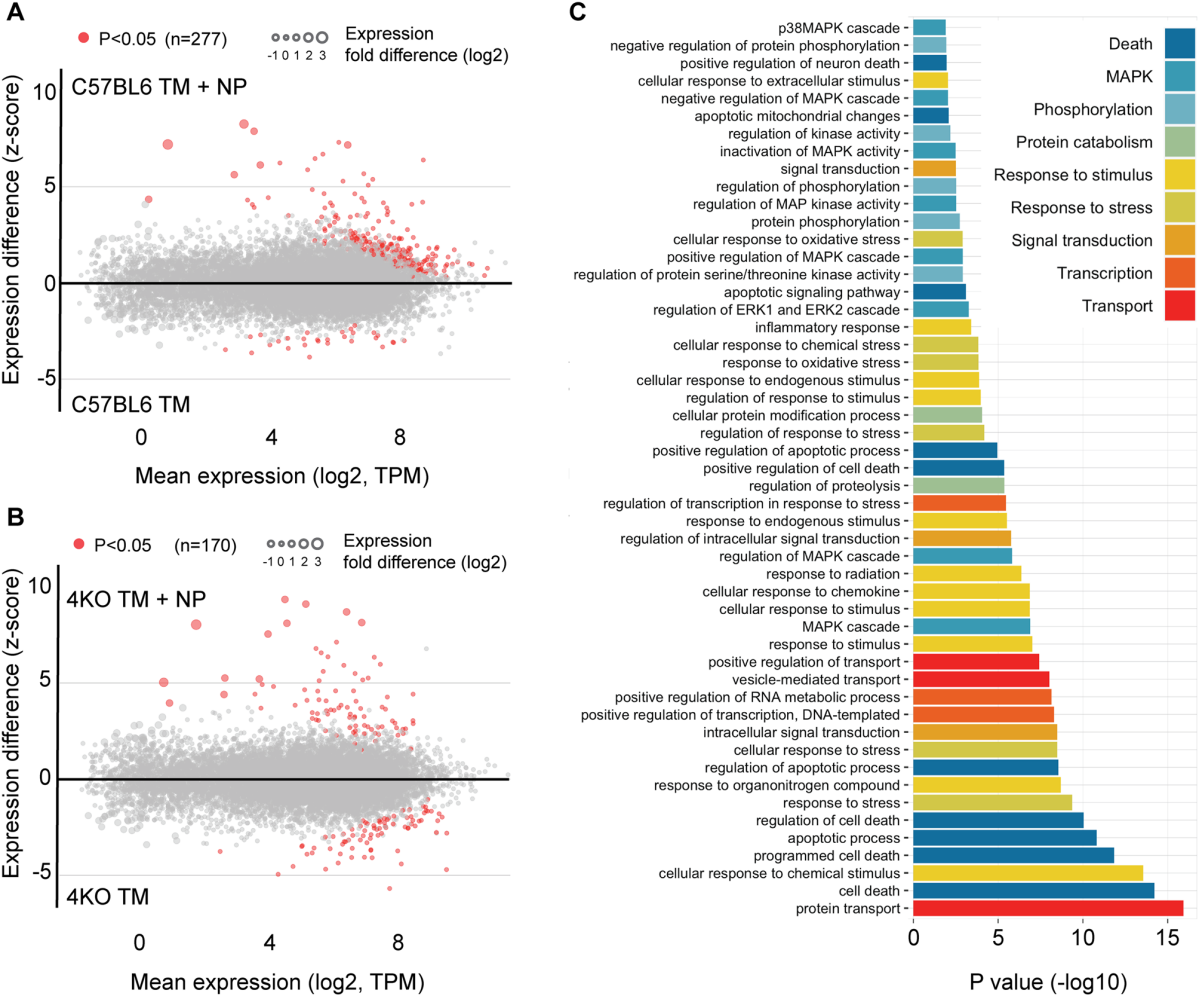
Synthetic nanoparticles trigger activation of MAPK signaling in TM-preconditioned macrophages. **(A)** Z-score plot of genome-wide differences in expression of genes in macrophages from WT mice treated with TM or TM+NP. Genes differentially-expressed between these two treatment conditions with *p* < 0.05, based on DESeq2 analysis, are shown in red. **(B)** Z-score plot of genome-wide differences in expression of genes in macrophages from 4KO mice treated with TM or TM+NP. Genes differentially-expressed between these two treatment conditions with *p* < 0.05, based on DESeq2 analysis, are shown in red. **(C)** The GO biological process gene sets that are overrepresented in TM-preconditioned BMDMs after the addition of NP, compared to BMDM treated with TM alone. The complete functional profiling of biological processes over-represented (*P* < 0.05) in TM-conditioned BMDM after NP treatment is shown in Table S2).

To identify which signal transduction pathways activated following the addition of NPs to TM-conditioned BMDM, we first characterized changes in global transcriptional programs that NPs trigger in WT and 4KO TM-conditioned BMDMs using a genome-wide RNAseq analysis. Differential gene expression analysis demonstrated that the addition of NPs to TM-treated BMDMs changed the expression of 277 genes in C57BL6-derived BMDMs and 170 genes in 4KO-derived BMDMs with *p* < 0.05 (Fig. 3A and B). We also found that although TM treatment alone did not trigger macrophage cell death, the addition of TM to cells triggered global changes in cell transcriptome with over 7,000 genes significantly changing expression after TM treatment compared to cells treated with control solvent DMSO (Supplemental Fig. S6 online). To identify signaling pathways that may drive a “cell death” phenotype after the addition of NP to TM-treated BMDMs, compared to BMDM treated with TM alone, using an entire genome-wide transcriptome data, we first conducted a principal component analysis with an imputed variable, which separates these two experimental conditions and extracted 500 genes that most strongly contributed to their divergence on a principal component PC-1 axis (Supplemental Fig. S7 and Supplemental Table S1 online). Gene ontology (GO) biological process over-representation analysis of these genes showed that all of them fall into a small number of biological processes groups broadly-defined as protein transport, cell death, signal transduction, and cell stress and MAPK pathway with P values from ranging from 0.05 to 10^–15^ (Fig. 3C and Supplemental Table S2 online). While over-representation of biological processes annotated as protein transport, cell death, and signal transduction was not surprising, a significant over-representation of biological processes annotated as response to stress and MAPK pointed to their potential involvement in mediating or driving a “cell death” phenotype observed after the addition of NP to TM-conditioned BMDMs.

**Figure 4:**
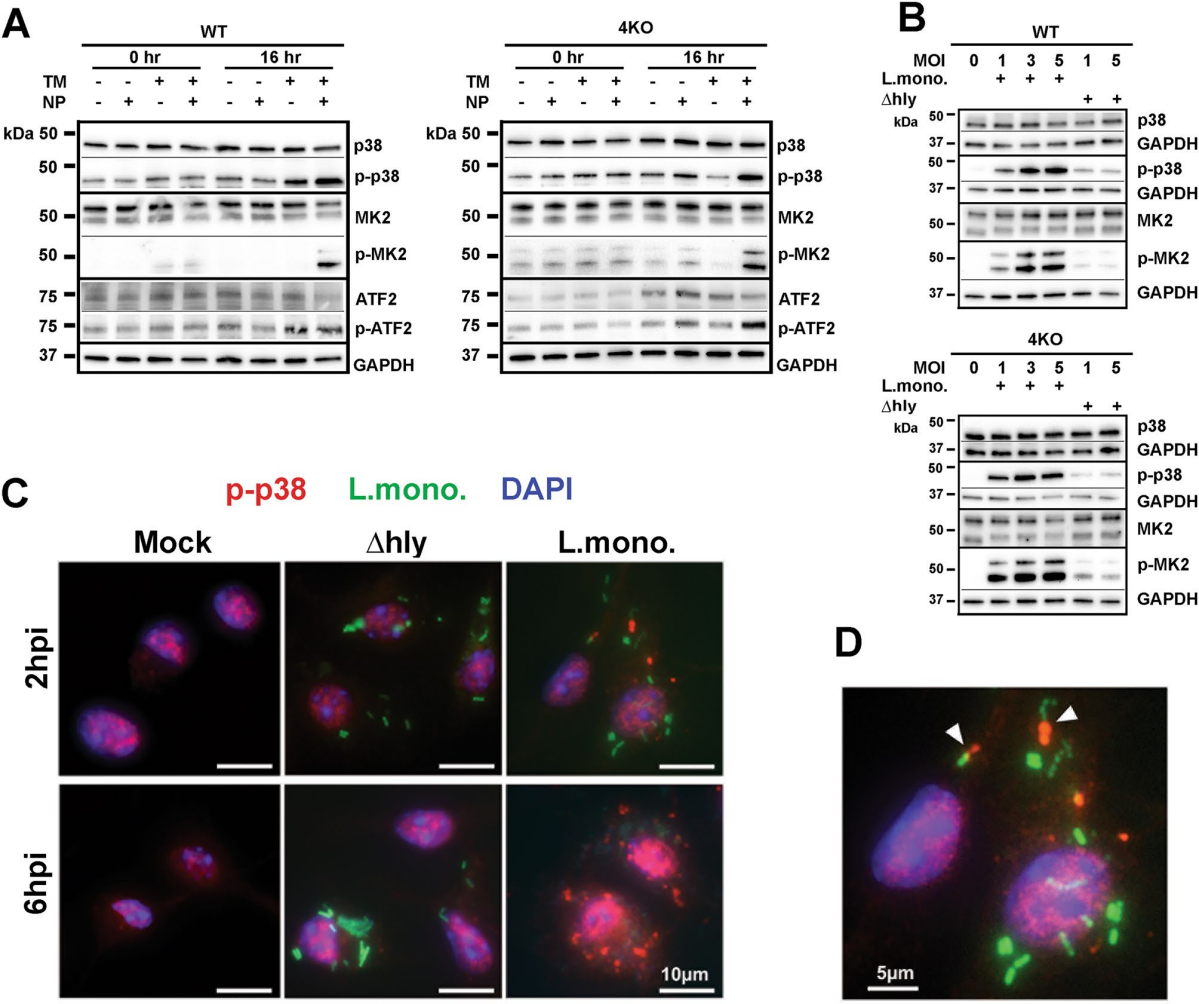
p38MAPK is activated in Mϕs after their exposure to *L.monocytogenes* or synthetic nanoparticles. **(A)** Western blot analysis of activation of p38 and its downstream signaling targets MK2 and ATF2 at indicated time points in WT and 4KO BMDMs after their treatment with DMSO (-), TM, NP, or TM and NP combination. N = 3. **(B)** Western blot analysis of activation of p38 and its downstream signaling target MK2 in WT and 4KO BMDMs after infection with wild-type *L. monocytogenes* (L. mono) or its isogenic strain (Δ*hly*) at 6 h post infection. N = 2. **(C)** Immunofluorescent staining of WT BMDM infected with CFDA-labeled (green) wild-type *L. monocytogenes* (L. mono) or its isogenic strain (Δ*hly*) at 2 h and 6 h post infection. Cells were stained for phosphorylated p38MAPK (p-p38) –specific antibody (red) and DAPI staining to show cell nuclei (blue). N = 3. **(D)** Enlarged cropped image from 2-h panel (**C**) for *L.monocytogenes*. Arrows point to phosphorylated p38MAPK staining (red) in proximity to the wild-type *L. monocytogenes* cells (green).

MAPK signaling pathway is fundamental for integrating and gauging cellular responses to a plethora of extracellular and intracellular stimuli^26^. MAPK pathway is highly evolutionary conserved, and it operates through a cascade of sequentially activated signal transducing serine/threonine protein kinases, many of which have overlapping substrate specificities. MAPK signaling ultimately culminates in activation of transcription factors that modulate expression of specific gene sets to mount the appropriate cellular response to a specific stimulus. Because cell stress and MAPK were significantly over-represented biological processes in NP-treated cells, we next analyzed whether NP addition to TM-treated BMDM activated MAPK signaling by evaluating phosphorylation state of key MAPK components AKT, ERK, JNK, and p38. This analysis demonstrated that neither TM nor NP treatment alone or in combination changed homeostatic amounts of these MAPK kinases in cells (Fig. 4A and Supplemental Fig. S8 online**)**. We also did not find activation of ERK, JNK, or JNK substrate c-JUN, and the amounts of phosphorylated AKT were reduced after NP addition to TM-treated BMDM (Supplemental Fig. S8 online). However, using antibody that recognizes all four mouse p38MAPK isoforms, p38α, p38β, p38γ, and p38δ, we found that the phosphorylation of p38MAPK was consistently increased and p38 downstream phosphorylation targets MK2 and ATF2 were both phosphorylated in response to NP in both WT and 4KO TM-conditioned BMDM (Fig. 4A). The analysis of activation state and the involvement of known upstream activators of p38MAPK in driving p38MAPK-dependent signaling demonstrated that the levels of phosphorylation of ASK1, TAK1, and TRAF2 did not change after NP addition at the time when cell underwent necrotic death (Supplemental Fig. S8 online). Neither shRNA-mediated ASK1 knockdown (Supplemental Fig. S9 online), nor the addition of pharmacological inhibitors of ROS or MK2 signaling could prevent BMDM necrosis in response to NP (Supplemental Fig. S9 online). Dose dependent increase in phosphorylated p38MAPK forms and the downstream p38MAPK target MK2 was also observed after infection of WT and 4KO BMDM with *L.monocytogenes* but not with isogenic Δ*hly* strain, which is unable to disrupt endosomal compartments (Fig. 4B). Next we analyzed intracellular distribution of CFDA-labeled *L.monocytogenes* cells and p-p38 using fluorescent microscopy. Staining of BMDM with antibodies specific for p-p38 revealed bright cytosolic puncta of positive p-p38 staining, localized in proximity to green-fluorescent bacteria cells at 2 h post infection (Fig. 4C and D). P-p38-specific staining puncta became bigger and more abundant in the cytosol and also in the nucleus by 6 h post infection (Fig. 4C), which is consistent with an increase in p-p38 amount detected by western blotting at later time points, compared to its homeostatic levels.

### p38MAPK mediates necrotic Mϕ death in response to *L.monocytogenes* or synthetic nanoparticles

**Figure 5:**
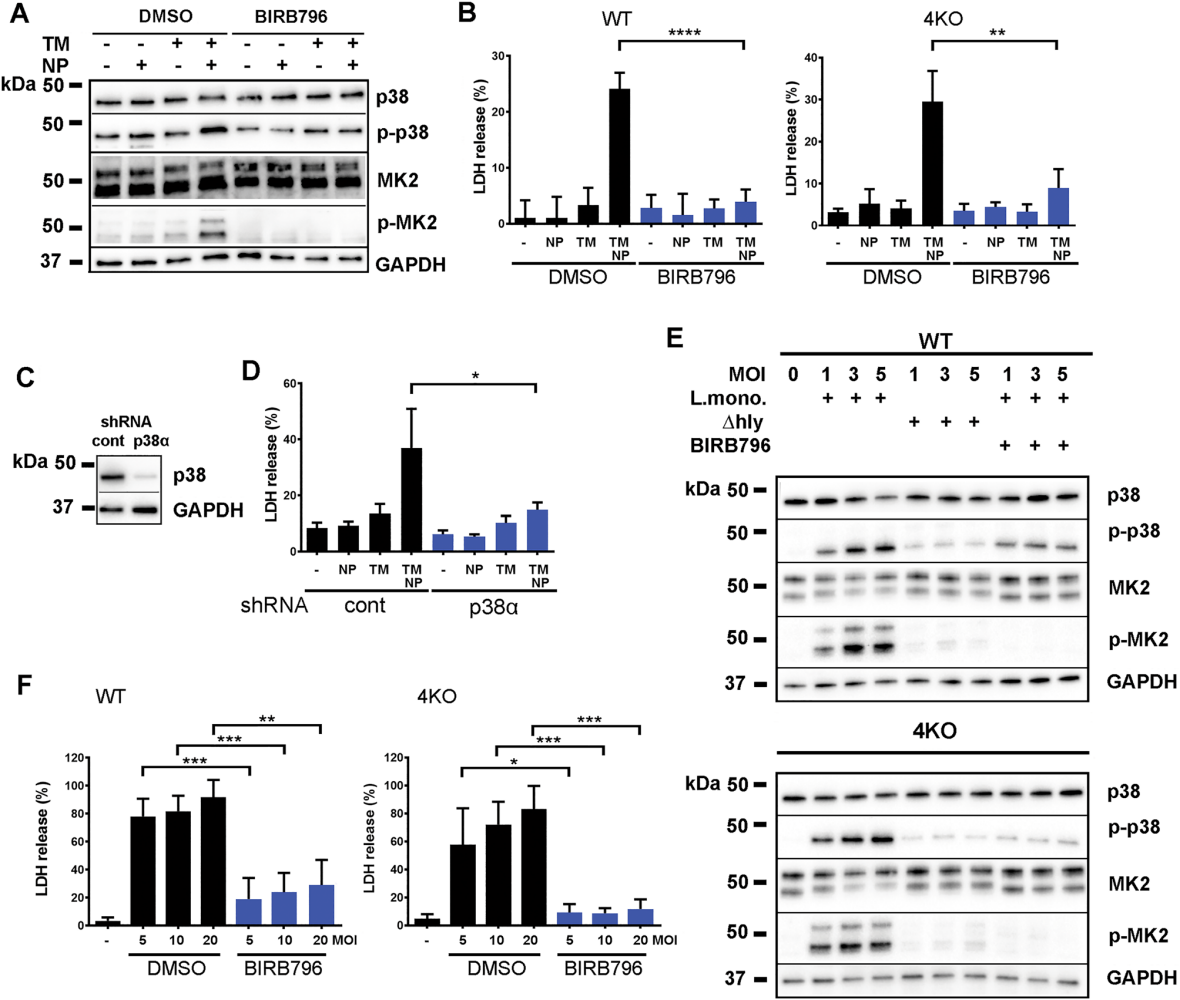
p38MAPK mediates necrotic death of BMDM after their exposure to *L.monocytogenes* or synthetic nanoparticles. **(A)** Western blotting analysis of BIRB796-mediated inhibition of p38 and MK2 activation in 4KO BMDMs after their treatment with DMSO (-), TM, NP, or TM and NP combination. N = 2. **(B)** LDH release from WT and 4KO BMDMs in the presence or absence of BIRB796, after cell treatment with DMSO (-), TM, NP, or TM and NP combination. N = 4–5. *P* = 0.0031 (4KO) and *P* < 0.0001 (WT). **(C)** Western blotting analysis of p38 protein expression in 4KO BMDMs after their infection with lentiviruses expressing control (cont) or p38α-specific shRNAs. N = 3. **(D)** LDH release from 4KO BMDM stably expressing control or p38α-specific shRNAs after their treatment with DMSO (-), TM, NP, or TM and NP combination. N = 3, *P = 0.0219. **(E)** Western blotting analysis of activation of p38 and its downstream signaling target MK2 in WT and 4KO BMDMs after their infection with *L.monocytogenes* of a wild-type and Δ*hly* strains at indicated MOIs, and inhibition of p38 signaling by BIRB796 during *L.monocytogenes* infection. N = 2. **(F)** LDH release from 4KO and WT BMDM in the absence or presence of BIRB796 after their infection with *L.monocytogenes* at indicated MOIs. N = 4.

To define whether the activation of p38 MAPK is a cell-death irrelevant event or it may be mechanistically implicated in the cell-death-promoting signaling, we first added pan-p38 MAPK inhibitor BIRB796^27^ to TM-treated BMDM prior to the addition of NP. The addition of pan-p38 inhibitor reduced p38 phosphorylation to a background levels and eliminated phosphorylation of p38-downstream signaling target MK2 (Fig. 5A). The addition of p38 inhibitor also significantly prevented NP-induced death of WT and 4KO BMDMs (Fig. 5B). To exclude potential off-target effects of p38 inhibiting drug, we knocked down major p38MAPK isoform, p38α, in 4KO BMDM using p38α-specific shRNA (Fig. 5C). The knockdown of p38α significantly reduced necrotic death of 4KO BMDM after NP exposure (Fig. 5D). Once again, only a wild-type *L.monocytogenes* but not its isogenic Δ*hly* strain, which is unable to rupture cellular phagosomal compartments, triggered p38 and MK2 phosphorylation in WT and 4KO BMDMs (Fig. 5E). Pre-treatment of cells with BIRB796 reduced phosphorylation of p38, completely prevented phosphorylation of MK2 (Fig. 5E), and prevented cell death of WT and 4KO BMDM after *L. monocytogenes* infection (Fig. 5F). We noticed that the infection of cells with Δ*hly* strain was also able to activate low-level p38 phosphorylation (Fig. 5E) without triggering cell death. This low-level p38 phosphorylation may occur due to the activation of cell surface receptors that may detect PAMPs present at the surface of both wild-type bacteria and its isogenic Δ*hly* strain. Collectively, these data implicate p38MAPK and its major isoform p38α as a key component of a necrotic cell death-promoting signaling, which is activated in response to a bacterium and a fully-synthetic NP, both of which compromise integrity of endosomal compartments of the cell.

### p38MAPK-dependent necrosis is distinct from necroptosis and is suppressed by RIPK1

**Figure 6:**
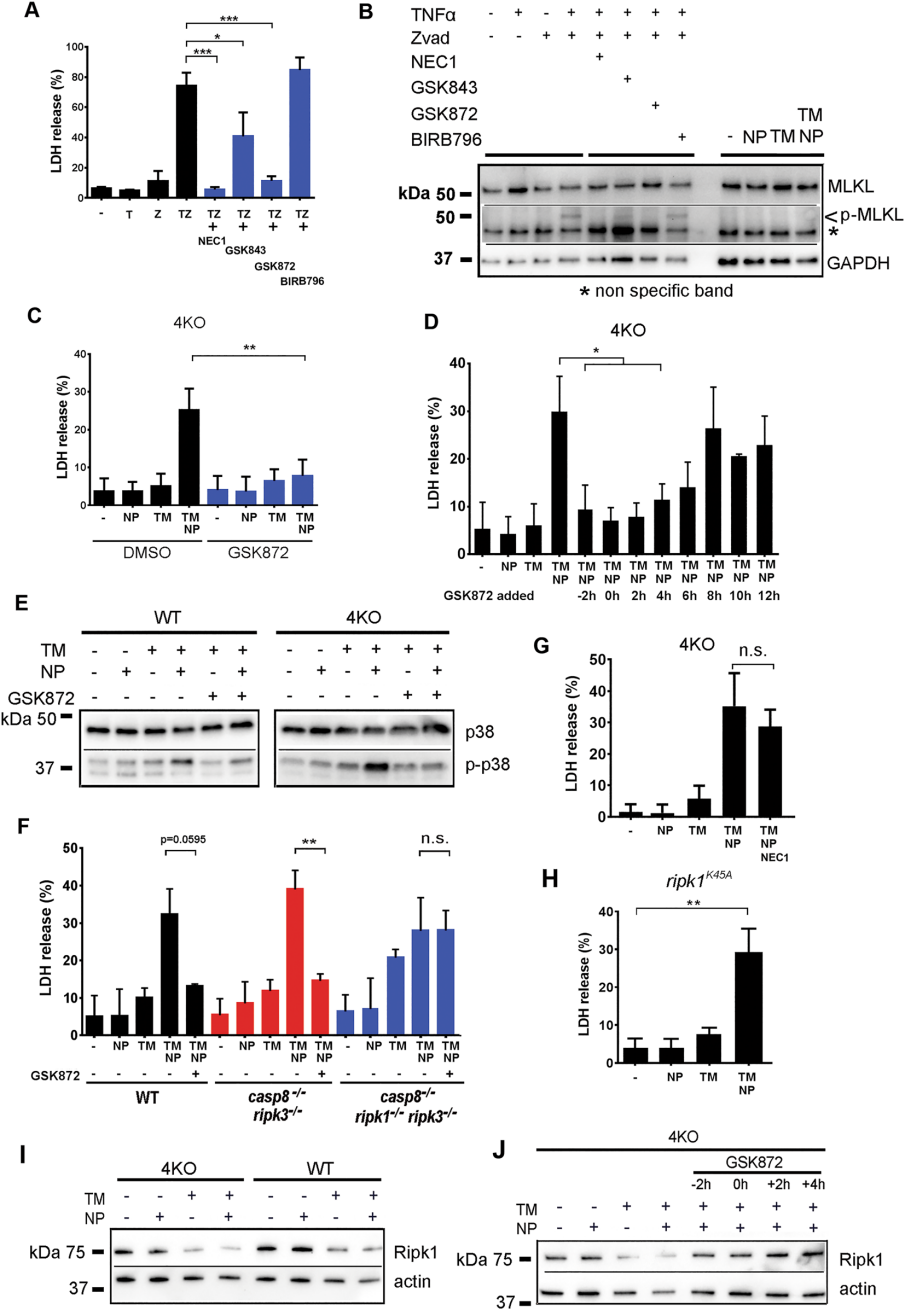
p38MAPK-dependent necrosis is distinct from necroptosis and is suppressed by RIPK1. (**A**) LDH release from WT BMDM treated with DMSO (-), TNFα (T), z-VAD-fmk (Z) or their combination in the absence or presence of indicated inhibitors. N = 3, *P = 0.0329, ***P = 0.0003. (**B**) Western blotting analysis of MLKL phosphorylation in WT BMDM after their treatment with necroptosis inducers TNFα and z-VAD in the absence or presence of inhibitors of necroptosis or after treatment with DMSO (-), TM, NP, or TM and NP combination. N = 3. (**C**) LDH release from 4KO BMDMs in the presence or absence of GSK872, after cell treatment with DMSO (-), TM, NP, or TM and NP combination. N = 4. *P* = 0.0017. (**D**) BMDM cultures from 4KO mice were conditioned with TM for 6 h, then nanoparticles were added (defined as 0 h point). GSK872 were added at different time points, cell death was determined by LDH activity release in culture media. N = 3. *P* = 0.02. **(E)** Western blotting analysis of GSK872-mediated inhibition of p38 phosphorylation in WT and 4KO BMDM after their treatment with DMSO (-), TM, NP, or TM and NP combination. N = 3. (**F**) LDH release from BMDM obtained from mice of indicated genotypes treated with DMSO (-), TM, NP, or TM and NP combination in the absence or presence of GSK872. N = 3. **P = 0.0015, n.s.–not significant. (**G**) Necroptosis inhibitor, necrostain-1 (NEC1) exhibits no protection in BMDM from 4KO mice upon TM and nanoparticle treatment determined by LDH activity release in the culture media. N = 4. (**H**) Necrotic cell death in BMDM from kinase-dead *Ripk1*^k45A^ mice upon TM and nanoparticle treatment was assessed by LDH activity in the culture media. N = 3. *P* = 0.0028. **(I)** Western blot analysis of RIPK1 expression in WT and 4KO BMDM after their treatment with DMSO (-), TM, NP, or TM and NP combination. N = 3. **(J)** Western blot analysis of RIPK1 expression in 4KO BMDM after their treatment with DMSO (-), TM, NP, or TM and NP combination in the absence or presence of GSK872 added at indicated times with regards to the addition of NP. N = 3.

To further exclude that p38-mediated necrotic death of BMDM occurs via a canonical necroptosis program, we treated WT BMDM with TNF and a pan-caspase inhibitor z-VAD with or without the addition of chemical inhibitors of necroptosis, NEC-1 (RIPK1 inhibitor^28^), GSK843 and GSK872 (RIPK3 inhibitors^29^), or pan-p38 inhibitor BIRB769. Consistent with previous reports^28^, a combination of z-VAD and TNF triggered death of BMDM, which could be inhibited by the addition of the inhibitors of necroptosis, but not by pan-p38 inhibitor BIRB769 (Fig. 6A). Although inhibitors of necroptosis could block phosphorylation of the terminal necroptosis effector MLKL^30^, BIRB769 failed to block MLKL phosphorylation upon cell treatment with TNF and z-VAD (Fig. 6B). Furthermore, neither the addition to cells of NP or TM, nor their combination could trigger MLKL phosphorylation at the time of BMDM death (Fig. 6B). In an attempt to exclude the contribution of components of the necroptotic pathway in mediating p38α-dependent necrosis, we next treated TM-conditioned BMDM from 4KO mice with inhibitors of necroptosis, prior to the addition of NP. Surprisingly, the addition of GSK872, a RIPK3-specific inhibitor, to a RIPK3-deficient 4KO BMDM completely prevented necrotic death in response to NPs (Fig. 6C), providing clear evidence that GSK872 acts on a target distinct from its previously defined substrate, RIPK3, in 4KO BMDMs, which were confirmed to be RIPK3-deficient both at mRNA (RNA-seq data files) and protein levels (Fig. 1C). GSK872 prevented 4KO BMDM necrotic death even when added to cells 4 h after the addition of NP (Fig. 6D), and p38 phosphorylation in response to TM+NP treatment was either greatly reduced (for WT cells) or completely blocked (for 4KO cells) (Fig. 6E). We hypothesized that to prevent cell death, GSK872 could target RIPK1, which is present in 4KO cells, and which is structurally homologous to RIPK3^5^. To test this hypothesis, we exposed TM-conditioned BMDM from WT, *casp8*^-/-^*ripk3*^-/-^, and *casp8*^-/-^*ripk1*^-/-^*ripk3*^-/-^ mice^31–33^ to NP with and without the addition of GSK872. This analysis revealed that GSK872 could prevent death of WT and *casp8*^-/-^*ripk3*^-/-^ BMDM, but not *casp8*^-/-^*ripk1*^-/-^*ripk3*^-/-^ BMDM, which lack RIPK1 (Fig. 6F). Because NEC-1 was unable to block NP-triggered necrosis (Fig. 6G), and NP triggered necrosis in TM-conditioned BMDM from mice with a RIPK1^K45A^ knock-in mutation, rendering RIPK1 catalytically inactive^31,34^ (Fig. 6H), catalytic activity of RIPK1 is unlikely to play a role in triggering of or protecting from the p38-dependent necrotic death. Instead, the known RIPK1 scaffolding function and pro-survival signaling^31,35^ may play a role in suppressing Mϕ necrosis mediated by p38. We, next, analyzed the amounts of RIPK1 in both WT and 4KO BMDMs upon their exposure to TM and NP. This analysis showed that TM treatment alone or in combination with NP leads to a reduction in the amount of intracellular RIPK1 (Fig. 6I). Importantly, the addition of GSK872 to cells resulted in stabilization of the amounts of RIPK1 even in the presence of TM and NP (Fig. 6J). Because both pro-survival and pro-death RIPK1 signaling can occur upon activation of TNFRI or pro-death signaling can occur downstream of TLR4/TRIF signaling, we next analyzed whether macrophages derived from mice deficient in TLR4, TRIF, or TNFRI may be protected from NP-induced death. Our analyses showed that deficiency in TLR4, TRIF, or TNFRI does not protect cells from *L.monocytogenes* or TM+NP-induced death (Supplemental Fig. S9 online). Collectively, this data provides evidence that in contrast to the canonical necroptosis signaling, RIPK1 enables cells survival by suppressing pro-death p38MAPK signaling under the WT conditions, or under the conditions when all major pathways of necrotic death have been eliminated (in 4KO cells).

## Discussion

Regulated cell death of necrotic types plays important roles in host defense against viral and bacterial pathogens, tissue homeostasis, and in the formation of effective anti-cancer immunity^2,4,5,10,20,36^. Earlier studies provided evidence that caspase-1-driven pyroptosis is an important mechanism of host protection against cytosolic bacteria^12,37^. Upon detecting bacterial PAMPs through specific cytosolic sensors, caspase-1-activating inflammasome complexes are formed^38^. This leads to proteolytic cleavage of caspase-1 and its downstream pyroptotic effector Gasdermin D, whose N-terminal domain forms pores in the plasma membrane, leading to necrotic cell death^39^. Gasdermin D also functions as an ultimate terminal effector of pyroptosis downstream of activated Caspase-11^39,40^, which senses LPS of gram-negative bacteria in the cytosol^41,42^. RIPK3-MLKL-dependent necroptosis is an important mechanism of host antiviral defense, as several viral pathogens, including CMV, HSV, and VACV, express specific proteins that interfere with this cell death pathway^43^, and viral PAMPS are detected by several upstream host sensors that converge their signaling on RIPK3 activation and execution of MLKL-dependent necroptosis^44–47^. Recent analyses suggest that in response to IAV infection, an interferon-inducible protein ZBP1 (DAI) drives the assembly of a death-inducing complex, PANaptosome, which simultaneously activates caspase-1 and caspase-8, as well as RIPK3 to execute cell death through activation of canonical terminal effectors of pyroptosis, apoptosis, and necroptosis^20^. Genetic deficiency in ZBP1 abrogates cell death induction and makes hosts highly susceptible to IAV infection, leading to early lethality^17,18^. Together, these data provide clear evidence that cytosolic pathogen sensing and prompt execution of death of an infected cell are the principal mechanisms of host innate immune defense against invading pathogens.

Our study showed that p38MAPK plays direct and non-redundant role in activating a physiologically-regulated necrosis in Mϕs in response to endosome rupture. Moreover, using genetic evidence, we show that p38-dependent necrosis does not require all established canonical mediators of necrotic-type death including Caspases-1, -11, RIPK3, and apoptotic caspase-8^48^. Previous studies with *L. monocytogenes* revealed that the ability to penetrate endosomal compartments correlated with the induction of necrotic death of liver Mϕs in vivo^49^. Although those studies implicated endosome rupture as the initiating event, the complexity of the pathogens and their many PAMPs as well as the redundancy in a substrate specificity of principal components of the MAPK signaling cascade made formal evidence on specific pathways involved difficult to assemble. Here, we analyzed Mϕ responses to fully synthetic chitosan-based NPs, which have limited number of PAMPs but effectively rupture cellular phagosomes (Supplemental Fig. S2). Similar to *L. monocytogenes*, NPs triggered necrotic Mϕ death, thus providing for the first time direct evidence that specific PAMPs are not required to trigger necrotic Mϕ death but rather the endosomes may function as pathogen sensors as a functional perturbation in a form of endosome rupture is necessary and sufficient for 38MAPK activation, which triggers Mϕ necrosis.

The rupture of cellular endosomal compartment, resulting in the release of endosomal contents into the cytosol, is a primordial as well as a prototypical danger signal. Recent studies suggests that galectins may play a role in activation of autophagy and inhibition of mTOR signaling in response to endomembrane damage in non-immune HEK293 and HeLa cells^50^. It was also shown that lysosomal damage activates AMPK through galectin-9 in a TAK1-dependent manner^50^. Using HeLa cells as a model, it was also determined that galectin-8 is a principal sensor of damaged endosomes and lysosomes after sterile injury as well as upon cell infection with *S.typhimurium*, *L.monocytogenes*, and *S.flexneri*, and galectin-8-dependent recruitment of an adaptor protein NDP52 activates autophagy, which limits pathogen burden inside infected cells^51^. In contrast to the autophagy-inducing function of galectin-8, in response to *L.monocytogenes*-induced endomembrane damage, galectin-3 suppresses activation of autophagy in mouse macrophages, allowing for efficient intracellular replication of bacteria even in the presence of damaged endo/phagosomes^52^. Whether galectin-induced signaling activates p38MAPK-dependent pro-necrotic signaling remain unknown. Our analyses showed that TAK1 phosphorylation levels do not change upon cell treatment with TM+NP at the time of cell death execution. It was previously shown that p38α isoform can autophosphorylate itself and activate its downstream signaling targets in a TAB1-dependent manner, independently of kinase activities of canonical upstream p38MAPK activators^53^. Whether autophosphorylation or canonical activation is the mechanism of p38MAPK activation in response to endosome rupture requires further studies.

Our study demonstrates that p38MAPK signaling leads to execution of necrosis and in our experimental system, RIPK1 plays a pro-survival role by protecting cells from death in response to endosome rupture. While pre-treatment of cells with TM leads to a global de-regulation of transcription for over 7,000 genes (Supplemental Fig. S6 online), which undoubtedly leads to a dramatic phenotypic changes in TM-treated cells compared to mock-treated controls, one of the effects of TM conditioning we found was a reduction in the amounts of intracellular RIPK1, which correlated with sensitizing cells to NP-induced necrotic death. Although TM treatment did not appear to reduce the amount of intracellular RIPK1 in MEF cells^54^, recent studies using primary human melanocytes^55^ and primary human melanocyte HEMn-MP cells^56^ showed that TM treatment leads to a reduction of intracellular RIPK1, similar to finding in our system, when primary mouse BMDM were cultured on serum-treated glass slides. We further found that a necroptosis inhibitor GSK872, which was previously determined to function by blocking catalytic activity of RIPK3, was able to stabilize amounts of RIPK1 in TM-treated cells and protect cells from NP-induced necrotic death. Although it is plausible that GSK872 may bind RIPK1 without blocking its catalytic activity due to structural similarity between RIPK3 and RIPK1, and thus prevent its degradation following TM treatment, our study cannot formally exclude that GSK872 may bind to and/or block the activity of other upstream regulator(s) of RIPK1, thus indirectly affecting RIPK1 stability. Further studies are needed to ascertain these possibilities.

p38MAPK and its phosphorylation target MK2 were recently implicating as key suppressors of RIPK1 pro-apoptotic function downstream of TNF receptor signaling^57–59^. Mechanistically, TNF binding to TNF receptor activates RIPK1, leading to its ubiquitination and activation of pro-survival TAK1-p38MAPK-MK2 signaling axis, which serves as a negative feedback loop whereby MK2 phosphorylates RIPK1 S321 and S336 and prevents its pro-death activity^57–59^. Therefore, pro-survival p38MAPK-MK2-dependent signaling plays an important host protective role by preventing severe TNF-dependent inflammatory responses and death. In the context of cancer therapy, pharmacologic targeting of p38 and MK2 was shown to enhance anti-leukemic activity of smac-mimetic by potentiating TNF production, TNF-dependent apoptosis, and tumor cell death^60^. In contrast to the pro-survival role of p38-MK2 signaling in response to TNF, our analyses showed that in response to endosome rupture, p38MAPK activation triggers signaling leading to cell necrosis. We found that the potential upstream p38MAPK activators TAK1 and ASK1 do not play a role in phosphorylating p38 as phosphorylation of neither TAK1 nor ASK1 is increased in macrophages in response to TM+NP treatment and shRNA knockdown of ASK1 does not protect cells from NP-induced death. Furthermore, although MK2 is clearly phosphorylated downstream of p38 signaling in our system, pharmacological inhibition of MK2 did not lead to cell protection from death, suggesting that MK2 is not the principal target of pro-death p38 signaling in response to endosome rupture. Our analyses also showed that RIPK1 plays a pro-survival role and stabilizing the amounts of RIPK1 by GSK872 protects macrophages from NP-induced cell death. This data suggests that TNF and TNFRI-signaling may not be engaged in sensing endosome rupture events. Consistently with this idea, we found that BMDM isolated from p55 TNFRI-deficient (*Tnfrsf1a*^*tm1Imx*^) mice still undergo necrotic death after TM+NP treatment. Future studies should determine whether p38-mediated necrosis is a regulated or unregulated cell death mechanism. Specifically, it remains unclear whether cell death executioners of the known cell death pathways are ultimately engaged either individually or in a functionally redundant, “flexible” manner^12^ to trigger macrophage necrosis in response to endosome rupture.

Taken together, similar to a two-step inflammasome activation model^9,38^, our study points to a two-step model of a danger-induced regulated necrosis induction/execution, where under homeostatic conditions, tonic RIPK1 signaling indicates a lack of danger and p38MAPK signaling is maintained at homeostatically low levels. However, under the stress conditions, where a pro-survival RIPK1 signaling is reduced, the second signal in a form of endosome rupture activates p38MAPK, ultimately leading to the execution of necrosis, which in the context of bacterial and viral infections was shown to play host-protective functions^12,19,37,49^. Because cell stress is universally associated with many degenerative diseases and aging, p38-dependent necrosis may play critical pathological role in a broad spectrum of human diseases, beyond of its immediate role in host defense against pathogens.

## Materials and methods

### Animal studies and mouse strains

All animal studies were conducted in accordance with the National Institutes of Health (NIH) Guide for the Care and Use of Laboratory Animals. Animal protocols were approved by the Institutional Animal Care and Use Committee (IACUC) of the Emory University under the protocol number PROTO201700034. For tissue harvesting, mice were sacrificed through cervical dislocation without anesthesia following institutional standardized procedure “Cervical Dislocation”, which was approved by Emory University IACUC under the protocol number PROC00016339. Manual cervical dislocation without anesthesia is an acceptable method with conditions and was utilized as per the recommendations of the most current edition of AVMA Guidelines for the Euthanasia of Animals. All individuals performing this technique were trained by authorized Emory University Division of Animal Resources’ personnel to ensure proficiency. The identity of strains was examined periodically using PCR and Western blotting. C57BL/6 mice were purchased from Charles River, Wilmington, MA. 129S1 mice (stock #2448) were purchased from Jackson Laboratories. All mice were matched by age and housed on campus of Emory University in specific-pathogen-free and a dedicated enhanced biosecurity (EBS) animal housing facility, which additionally excludes several commensal and opportunistic pathogenic bacteria. All methods are reported in accordance with ARRIVE guidelines.

### Bacterial culture and strains

Wild-type *L. monocytogenes* and isogenic mutant Δ*hly* strain were streaked on BHI agar plates from frozen stocks. Stationary cultures were initiated from single colonies and incubated at 30◦C with shaking overnight. Three to 4 h prior to bacteria administration, fresh cultures were initiated from overnight cultures and incubated at 37 °C with shaking.

### Preparation and formulation of nanoparticles

Nanoparticle preparations were made fresh for each experiment and were standardized to produce consistent levels of macrophage death, as measured by propidium iodide positivity in vitro. Chitosan was purchased from [Novamatrix (PCL113, Sandvika, Norway)/Heppe Medical Chitosan GMBH (Item 55,040, Halle, Germany)] and sodium tri-polyphosphate (TPP) was purchased from Sigma-Aldrich (St. Louis, MO). The chitosan was dissolved in sterilized 200 mM sodium acetate buffer (pH = 4.5). Chitosan and TPP were mixed at a mass ratio of 5:1, vortexed for 30 s, and then rotated for 30 min at room temperature. Sodium acetate buffer was removed from the nanoparticles by centrifugal filtration at 4000 g for 20 min using Amicon filters (4 mL, MWCO = 100,000). Nanoparticles were resuspended in sterile PBS at a concentration of 1 mg/mL before use in experiments.

### Western-blot analyses

Macrophage lysates were mixed with SDS-PAGE sample buffer and heated at 95 °C for 5 min. Denatured macrophage lysates were electrophoresed using pre-casted 4–15% gradient gels (Bio-Rad, Hercules, CA). The proteins were transferred to polyvinylidene difluoride membranes (Millipore, Billerica, MA) for Western-blotting analysis. Antibodies used are listed in Table S3. After washing un-bound primary antibodies, blots were incubated with the appropriate horseradish peroxidase (HRP) conjugated secondary antibody, washed and specific protein bands were visualized using an enhanced chemiluminescence detection kit (Bio-Rad, Hercules, CA).

The same blots were re-probed with an anti- GAPDH (Thermo Fisher Scientific Inc, Suwanee, GA), or anti-actin (Sigma, St. Louis, MO) antibody to assess equal loading.

### BMDM culture and in vitro death induction with tunicamycin and chitosan nanoparticles

Bone marrow cells were harvested from femurs of mice. Marrow cells were re-suspended in DMEM media supplemented with 10% FBS (Life Technologies, Carlsbad, CA), 1 × pencillin/ streptomycin (Life Technologies, Carlsbad, CA) and 40 ng/ml of macrophage colony–stimulating factor, mouse M-CSF (R&D, Minneapolis, MN). After 6 days differentiation, bone marrow derived macrophages (BMDM) were retrieved and plated on FBS pre-treated 4-well glass bottom slides (Thermo Fisher Scientific Inc, Suwanee, GA) at 2–2.2 × 10^5^ cell/well. One day later, culture media were changed with phenol-red free DMEM with 20 ng/ml of M-CSF in the presence of DMSO or 0.5 µg/ml of tunicamycin (Sigma) for 6 h. Chitosan nanoparticles were added after 6 h treatment at final 29–55 µg/ml at which concentration minimum cytotoxicity of nanoparticle was observed, equal volume of PBS was added in controls. Fourteen to 16 h later, cell death assessment was performed by propidium iodide (PI) uptake and lactate dehydrogenase (LDH) assay. Finally, cell lysate were prepared using lysis buffer containing protease and phosphatase inhibitors (Cell Signaling Technology, Danvers, MA). Various chemical inhibitors or vehicle control were added to culture media 2 h before nanoparticle treatment, and the list of chemicals used is listed in the Table S4.

### Cell death assessment with propidium iodide (PI) uptake and lactate dehydrogenase (LDH) assay

Fourteen to sixteen hours after nanoparticle treatment, necrotic cell death were determined by cell membrane-impermeant DNA dye propidium iodide. 2.5 µg of PI were added to each well and fluorescence images were taken with a Zeiss Axio Observer microscope (Oberkochen, Germany). Four images were taken for each experimental condition at each time point. The acquired images were analyzed with IncuCyte analysis software. The phase object confluence (%) was used to indicate total cell number and red object confluence (%) to indicate necrotic cells. Red object confluence at different time point was divided by the starting time Phase object confluence as arbitrary death index. To analyze the time-course of the cell death execution in BMDM, cells were continuously imaged using IncuCyte machine with manufacturer’s recommended cell death dye present in the media. Live-cell imaging and kinetic evaluation of necrotic cell death in BMDMs was performed with Incucyte live-cell analysis instrument (Essen Biosciences).

Necrotic cell death was also determined by measuring the LDH enzyme activity in the culture media. Culture media were spun at 1000 × g for 10 min to remove cell debris. LDH activity in the supernatant was determined using an assay kit from Sigma (St. Louis, MO) following manufacture’s instruction. To determine the percentage of cell death in each experimental setting, the amount of LDH activity determined upon different treatments was normalized to a total LDH activity present in cells plated in control wells which were left without any treatment and expressed as a percent of total LDH. For the vast majority of experiments (unless stated otherwise), experiments in gene-deficient cells or mice were done together with WT cells. All experiments were repeated two or more times with similar findings, which are reported.

### Lentiviral shRNA knockdown of endogenous proteins in BMDM

MISSION™ shRNA Lentiviral constructs targeting specific proteins and a mammalian non- specific shRNA control were purchased from Sigma (St. Louis, MO). ShRNA lentivirus were prepared by co-transfection of shRNA lentiviral vector, psPAX2 and pMD2.G (Addgene, Cambridge, MA) in HEK293 cells. Viral media were harvested twice from the culture and filtered through 0.45 μm pore PVDF membranes and concentrated by centrifuging at 6700 × g for a minimum of 16 h at 4 °C. The virion pellets were re-suspended in MEM and stored at − 80 °C. Lentivirus titers were semi-quantified by Lenti-X GoStix (Clonetech, Mountain View, CA).

Bone marrow cells were isolated from mouse femurs. Cell suspensions were transduced with shRNA lentivirus in Retro-nectin (Takara Bio Inc, Mountain View, CA) coated non-tissue culture plate for overnight in the presence of 50 ng/ml of mSCF (R&D, Minneapolis, MN). After overnight transduction, bone marrow cells were harvested and washed with cold PBS and plated in non-tissue culture plates. BMDM were differentiated as described before and transduced cells were selected by adding fresh puromycine at 3 µg/ml at Day 3 and Day 5. At Day 7 transduced mature macrophages were used for cell death experiment. Protein knockdown efficiency was confirmed by Western- blotting analyses.

### Immunofluorescence of cultured BMDM

BMDM cells were infected with CFSE-labeled *L.monocytogenes*, Δ*hly*, or incubated with NP and fixed at indicated times. After fixation, cells were permeabilized with 0.2% Triton X-100 (Sigma Aldrich) for 5 min at RT. Cells were washed with nuclease free PBS and blocked with 5% BSA in PBS for 30 min at 37 °C. Next, cells were incubated for 30 min at 37 °C with primary antibodies (Ab) diluted in PBS. Primary Abs used were mouse anti-clathrin light chain (1:500, Biolegend, Cat. No. MMS-423P), rabbit anti-caveolin-1 (1:200, Santa Cruz, Cat. No. sc-894), and rabbit anti-Rab7 (1:100, Abcam, Cat. No. ab137029). Rabbit anti-p-p38 (1:200, CellSignaling, #9211). Cells were then washed with PBS and incubated for 30 min at 37 °C with secondary Abs. Secondary Abs used were donkey anti-mouse conjugated to Alexa Fluor 546 (Thermo Fisher) or donkey anti-rabbit conjugated to Alexa Fluor 647 (Thermo Fisher). Goat anti-rabbit IgG Alexa-Flour-549. Finally, cells were washed with PBS and stained with DAPI for 5 min. Glass coverslips were mounted onto stained cells using Prolong Gold (Thermo Fisher).

### Microscopy and co-localization analyses

Images for co-localization analysis were acquired with a Hamamatsu Flash 4.0 v2 sCMOS camera on a PerkinElmer UltraView spinning disk confocal microscope mounted to a Zeiss Axiovert 200 M body with a 63 × NA 1.4 oil plan-apochromat objective. Z-stacks were taken in 0.2 µm increments with Volocity acquisition software (PerkinElmer, Waltham, MA). Co-localization analysis with clathrin light chain and cavolin-1 for nanoparticles was performed using Volocity software. Briefly, regions of interest were drawn around each cell containing nanoparticles, and the ‘Calculate Object Co-localization’ function was used, with background threshold levels manually set, to obtain the Mander’s overlap coefficient on a per cell basis. Co-localization analysis with Rab7 for listeria was performed manually in Volocity by counting the number of listeria per cell that were engulfed in Rab7 as a percentage of total number of listeria per cell. To obtain intensity line profiles, image files were exported from Volocity as TIFF images and imported into Image J (NIH) for analysis using the RGB profiler plugin. Line profile results were plotted in Excel (Microsoft). Quantification data was plotted with Prism 7 (Graphpad). For image clarity, linear contrast enhancement was performed on images, and individual channels were pseudocolored as indicated. All analysis was performed on unenhanced data.

### RNAseq analysis

The BMDM were seeded in quadruplicates and treated with either TM along for 6 h, followed by the media treatment (mock) for 8 h or TM for 6 h followed by NP treatment for 8 h. The cells were harvested in RLT buffer (Qiagen) and submitted for RNA isolation and RNA sequencing on Illumina platform at the Nonhuman primate genomics core at Yerkes National Primate Research Center. The raw data was analyzed for QC and aligned to the mouse mm10 genome using DNASTAR software (Lasergene). The analysis of the RNA-seq data was performed in SeqMonk software (Babraham Bioinformatics, version 1.47.2). The aligned (*bam) sequencing files were loaded in SeqMonk, and raw counts were calculated using the SeqMonk RNA-seq pipeline. The raw counts were used for DESeq2 differential genes expression analysis in the SeqMonk and R environments. The tunicamycin (TM)-treated data sets were compared to TM + Nanoparticles (TM + nano)-treated samples from C57BL6 and 4KO mice (four replicates for each condition). The z-scores of differences were calculated using the R package "intensitydiff" with modifications. Briefly, the gene expression data were log2-normalized. The local mean differences were calculated for each 1% window (along the ranked by the expression level genes) and used for local StDev and z-scores calculations. The scatter plots highlight differentially expressed genes with adjusted (FDR/Benjamini-Hochberg) *p* values < 0.05, and the size of points reflects the fold change between the samples (log2).

We employed PCA (R package "factoextra") to find which genes contribute the most to the differences between dying (TM + nano) and alive (TM) treated cells. In detail, we used all expressed genes in the data sets to calculate the PCs. Then, to pull the dimension that describes the difference between TM + nano versus TM-treated cells to the surface, we assigned an additional variable that guided the separation between conditions. Finally, we selected 500 genes most contributing to the separation of conditions and analyzed functional overrepresentation using gProfiler (Raudvere) webserver against GO biological processes sets. The graphs were generated in R package "ggplot2".

### Statistical analyses

All experiments were repeated at least twice. Results are expressed as mean and standard derivation. Unless indicated otherwise, Student’s unpaired two-tailed t test was used for comparing experimental groups, with *P* < 0.05 considered statistical significant.

## Supplementary Information

Supplementary Information 1.

Supplementary Video 1.

Supplementary Video 2.

Supplementary Information 2.

Supplementary Information 3.

## Data Availability

The RNA-seq data presented in this manuscript are available in the Gene Expression Omnibus under the accession number GSE117042. All data are available in the main text or the supplementary materials. The full-length images of the original western blot membranes demonstrating expression of proteins, shown in all figures of the manuscript in a cropped form, are available in Supplemental Figures S10 to S44, online.
