# Supplementary material for: p38MAPK guards the integrity of endosomal compartments through regulating necrotic death
Source: Sci Rep. 2022 Sep 29;12:16357. doi: 10.1038/s41598-022-20786-4 (PMC9523023; doi:10.1038/s41598-022-20786-4)
Supplement: Supplementary file 1 — Supplementary Information 1. [file 41598_2022_20786_MOESM1_ESM.pdf]

Supplemental Materials for

p38MAPK Guards the Integrity of Endosomal Compartments through Regulating Necrotic Death

Jia Yao, Svetlana Atasheva, Randall Toy, Emmeline L. Blanchard, Philip J. Santangelo, Krishnendu Roy, Edward S. Mocarski, Dmitry M. Shayakhmetov*

*Corresponding author. Email: dmitryshay@emory.edu

This PDF file includes:

Legends to Movies S1 and S2
Legends to Tables S1 and S2
Figs. S1 to S44
Tables S3 to S4

Other Supplemental Materials for this manuscript include the following:

Movies S1 to S2
Tables S1 to S2

Legends to Supplemental Movies S1 and S2.

Movie S1

Treatment of BMDMs from MLKL-deficient mice with TM alone triggers no necrotic cell death, as determined by the addition to cell media PI (red). Time-lapsed images of treated cells were obtained with IncuCyte imaging system.

Movie S2

Treatment of BMDMs from MLKL-deficient mice with TM and NPs triggers necrotic-type cell death, as determined by the addition to cell media PI (red). Time-lapsed images of treated cells were obtained with IncuCyte imaging system.

Legends to Supplemental Tables S1 and S2.

Table S1.

The list of 500 genes that most strongly contributed to the divergence on a principal component PC-1 axis between transcriptomes of macrophages treated with tunicamycin alone compared to macrophages treated with a combination of tunicamycin and NP. Please see Methods section for more information on data processing pipeline.

Table S2.

The list of biological processes over-represented in macrophages treated with a combination of tunicamycin and NP compared to macrophages treated with tunicamycin alone based on Gene ontology (GO) biological process over-representation analysis. Please see Methods section for more information on data processing pipeline.

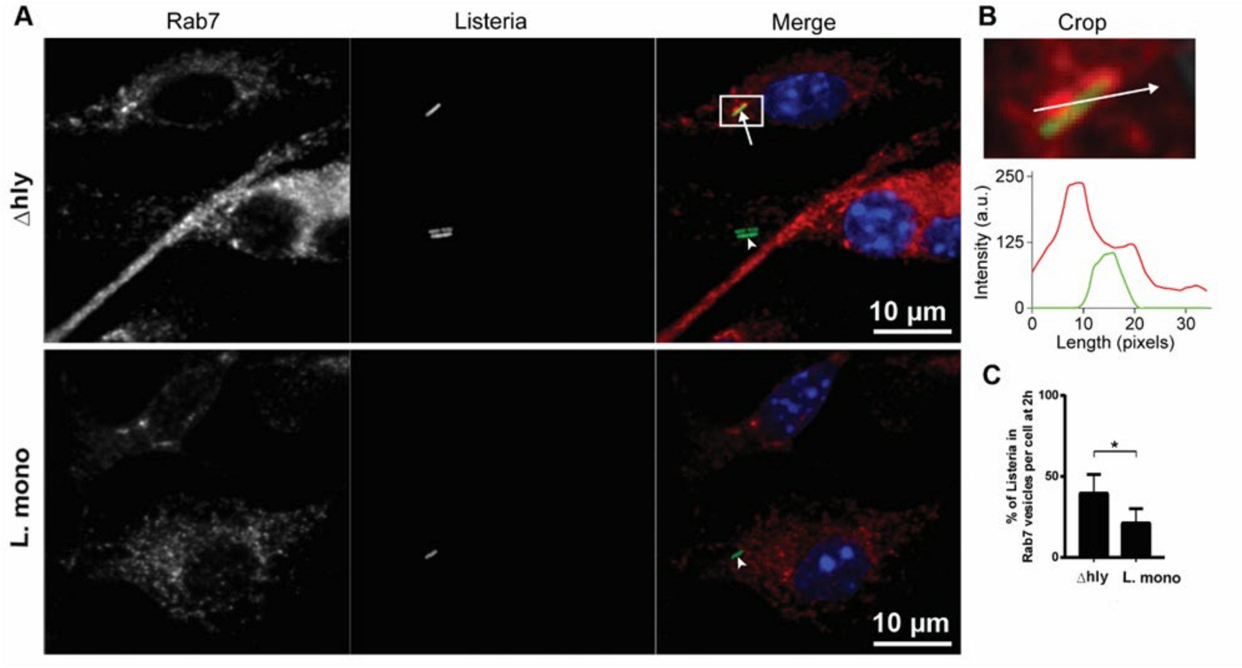

Fig. S1.

Co-localization analysis of mutant and wild-type *L. monocytogenes* or isogenic Δ*hly* strain with Rab7-containing endosomal vesicles. (**A**) Internalization of mutant (top) or wild-type (bottom) CFDA-labeled *L. monocytogenes* (green) in bone marrow derived macrophages at 2 h. Cells were stained for Rab7 (red) and nuclei (blue). Extended focus images are shown. Scale bar represents 10 μm. Arrow indicates listeria engulfed in Rab7-containing vesicle, while arrowheads indicate listeria not contained in Rab7-containing vesicles. (**B**) Enlarged cropped image from the white boxed region from part (**A**). Intensity profile shows co-localization along the white arrow. (**C**) Quantification of Mander's overlap coefficient between the bacteria cells and Rab7. Error bars represent 95% confidence intervals. N = 30 cells per group. Asterisks represent $P < .005$ by Tukey's Multiple Comparisons test.

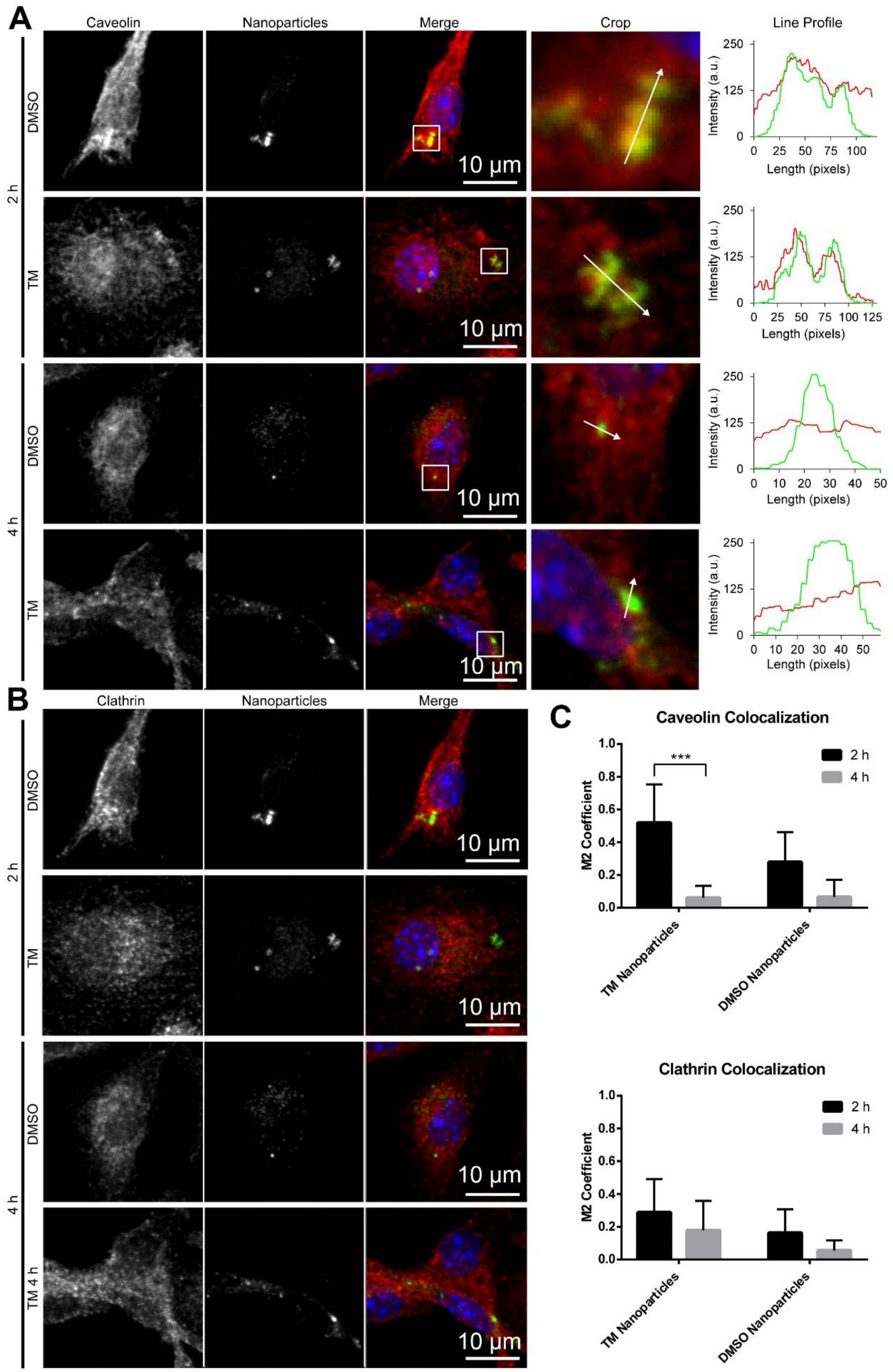
A
Caveolin
Nanoparticles
Merge
Crop
Line Profile
2 h
4 h
DMSO
TM
10 μm
Intensity (a.u.)
Length (pixels)
B
Clathrin
TM 4 h
C
Caveolin Colocalization
Clathrin Colocalization
M2 Coefficient
***
2 h
4 h
TM Nanoparticles
DMSO Nanoparticles

Fig. S2.

Colocalization analysis of nanoparticles with clathrin or caveolin-1. (A) Internalization of nanoparticles (green) in bone marrow derived macrophages at 2 h or 4 h treated with either DMSO or tunicamycin (TM). Cells were stained for nuclei (blue) and caveolin-1 (red). Extended focus images are shown. Scale bar represents 10 μm. Cropped images show enlargements from the white boxed images with intensity line profiles drawn along the white arrows. **(B)** Internalization of nanoparticles (green) at 2 h or 4 h treated with either DMSO or tunicamycin (TM). Cells were stained for nuclei (blue) and clathrin light chain (red). **(C)** Quantification of Mander's overlap coefficient between the nanoparticles and caveolin-1 or clathrin light chain as indicated under each treatment condition. Error bars represent 95% confidence intervals. N = 20 cells per group. Asterisks represent $P < .005$ by Tukey's Multiple Comparisons test.

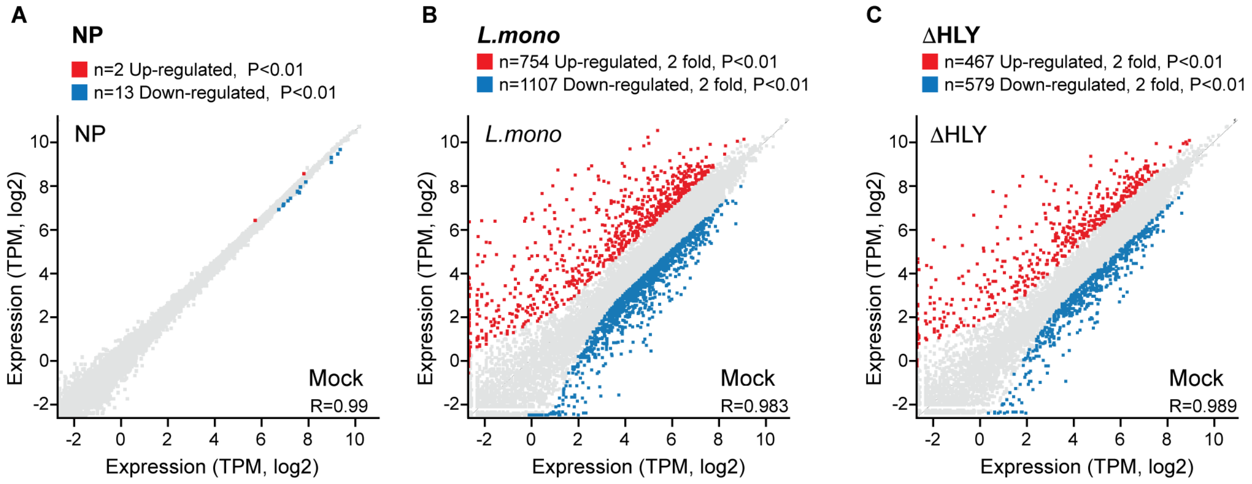

Fig. S3.

Nanoparticle treatment has lesser impact on BMDM transcriptional landscape than *L.monocytogenes* or Δ*hly* strain. Scatter plots of gene expression in BMDMs treated with **(A)** nanoparticles, **(B)** *L.mono,* **(C)** ΔHLY. The X-axis shows normalized gene expression in the Mock samples, the Y-axis shows the normalized gene expression in the treated samples. The red dots represent the genes that are significantly (P<0.01, DeSeq2) upregulated compared to the Mock sample, the blue dots represent the genes that are significantly (P<0.01, DeSeq2) downregulated compared to the Mock sample (A), the red and blue dots for (B) and (C) have more strict criteria and additionally have more than two-fold difference in expression compared to Mock. TPM – transcripts per million.

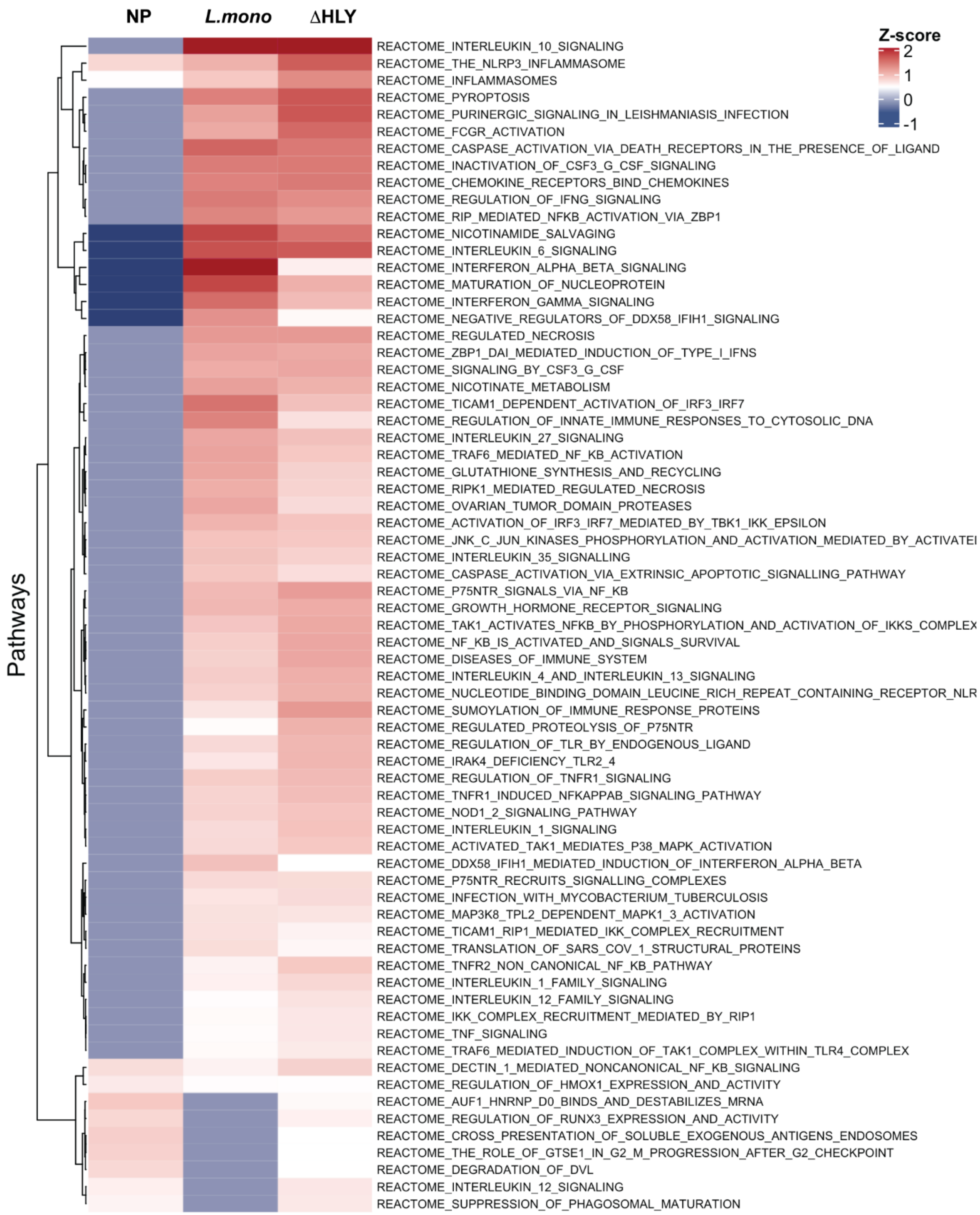
NP
L.mono
ΔHLY
Z-score
2
1
0
-1
Pathways
REACTOME_INTERLEUKIN_10_SIGNALING
REACTOME_THE_NLRP3_INFLAMMASOME
REACTOME_INFLAMMASOMES
REACTOME_PYROPTOSIS
REACTOME_PURINERGIC_SIGNALING_IN_LEISHMANIASIS_INFECTION
REACTOME_FCGR_ACTIVATION
REACTOME_CASPASE_ACTIVATION_VIA_DEATH_RECEPTORS_IN_THE_PRESENCE_OF_LIGAND
REACTOME_INACTIVATION_OF_CSF3_G_CSF_SIGNALING
REACTOME_CHEMOKINE_RECEPTORS_BIND_CHEMOKINES
REACTOME_REGULATION_OF_IFNG_SIGNALING
REACTOME_RIP_MEDIATED_NFKB_ACTIVATION_VIA_ZBP1
REACTOME_NICOTINAMIDE_SALVAGING
REACTOME_INTERLEUKIN_6_SIGNALING
REACTOME_INTERFERON_ALPHA_BETA_SIGNALING
REACTOME_MATURATION_OF_NUCLEOPROTEIN
REACTOME_INTERFERON_GAMMA_SIGNALING
REACTOME_NEGATIVE_REGULATORS_OF_DDX58_IFIH1_SIGNALING
REACTOME_REGULATED_NECROSIS
REACTOME_ZBP1_DAI_MEDIATED_INDUCTION_OF_TYPE_I_IFNS
REACTOME_SIGNALING_BY_CSF3_G_CSF
REACTOME_NICOTINATE_METABOLISM
REACTOME_TICAM1_DEPENDENT_ACTIVATION_OF_IRF3_IRF7
REACTOME_REGULATION_OF_INNATE_IMMUNE_RESPONSES_TO_CYTOSOLIC_DNA
REACTOME_INTERLEUKIN_27_SIGNALING
REACTOME_TRAF6_MEDIATED_NF_KB_ACTIVATION
REACTOME_GLUTATHIONE_SYNTHESIS_AND_RECYCLING
REACTOME_RIPK1_MEDIATED_REGULATED_NECROSIS
REACTOME_OVARIAN_TUMOR_DOMAIN_PROTEASES
REACTOME_ACTIVATION_OF_IRF3_IRF7_MEDIATED_BY_TBK1_IKK_EPSILON
REACTOME_JNK_C_JUN_KINASES_PHOSPHORYLATION_AND_ACTIVATION_MEDIATED_BY_ACTIVATE
REACTOME_INTERLEUKIN_35_SIGNALLING
REACTOME_CASPASE_ACTIVATION_VIA_EXTRINSIC_APOPTOTIC_SIGNALLING_PATHWAY
REACTOME_P75NTR_SIGNALS_VIA_NF_KB
REACTOME_GROWTH_HORMONE_RECEPTOR_SIGNALING
REACTOME_TAK1_ACTIVATES_NFKB_BY_PHOSPHORYLATION_AND_ACTIVATION_OF_IKKS_COMPLEX
REACTOME_NF_KB_IS_ACTIVATED_AND_SIGNALS_SURVIVAL
REACTOME_DISEASES_OF_IMMUNE_SYSTEM
REACTOME_INTERLEUKIN_4_AND_INTERLEUKIN_13_SIGNALING
REACTOME_NUCLEOTIDE_BINDING_DOMAIN_LEUCINE_RICH_REPEAT_CONTAINING_RECEPTOR_NLR
REACTOME_SUMOYLATION_OF_IMMUNE_RESPONSE_PROTEINS
REACTOME_REGULATED_PROTEOLYSIS_OF_P75NTR
REACTOME_REGULATION_OF_TLR_BY_ENDOGENOUS_LIGAND
REACTOME_IRAK4_DEFICIENCY_TLR2_4
REACTOME_REGULATION_OF_TNFR1_SIGNALING
REACTOME_TNFR1_INDUCED_NFKAPPAB_SIGNALING_PATHWAY
REACTOME_NOD1_2_SIGNALING_PATHWAY
REACTOME_INTERLEUKIN_1_SIGNALING
REACTOME_ACTIVATED_TAK1_MEDIATES_P38_MAPK_ACTIVATION
REACTOME_DDX58_IFIH1_MEDIATED_INDUCTION_OF_INTERFERON_ALPHA_BETA
REACTOME_P75NTR_RECRUITS_SIGNALLING_COMPLEXES
REACTOME_INFECTION_WITH_MYCOBACTERIUM_TUBERCULOSIS
REACTOME_MAP3K8_TPL2_DEPENDENT_MAPK1_3_ACTIVATION
REACTOME_TICAM1_RIP1_MEDIATED_IKK_COMPLEX_RECRUITMENT
REACTOME_TRANSLATION_OF_SARS_COV_1_STRUCTURAL_PROTEINS
REACTOME_TNFR2_NON_CANONICAL_NF_KB_PATHWAY
REACTOME_INTERLEUKIN_1_FAMILY_SIGNALING
REACTOME_INTERLEUKIN_12_FAMILY_SIGNALING
REACTOME_IKK_COMPLEX_RECRUITMENT_MEDIATED_BY_RIP1
REACTOME_TNF_SIGNALING
REACTOME_TRAF6_MEDIATED_INDUCTION_OF_TAK1_COMPLEX_WITHIN_TLR4_COMPLEX
REACTOME_DECTIN_1_MEDIATED_NONCANONICAL_NF_KB_SIGNALING
REACTOME_REGULATION_OF_HMOX1_EXPRESSION_AND_ACTIVITY
REACTOME_AUF1_HNRNP_D0_BINDS_AND_DESTABILIZES_MRNA
REACTOME_REGULATION_OF_RUNX3_EXPRESSION_AND_ACTIVITY
REACTOME_CROSS_PRESENTATION_OF_SOLUBLE_EXOGENOUS_ANTIGENS_ENDOSOMES
REACTOME_THE_ROLE_OF_GTSE1_IN_G2_M_PROGRESSION_AFTER_G2_CHECKPOINT
REACTOME_DEGRADATION_OF_DVL
REACTOME_INTERLEUKIN_12_SIGNALING
REACTOME_SUPPRESSION_OF_PHAGOSOMAL_MATURATION

Fig. S4.

Macrophage treatment with bacteria activates broader range of innate immune pathways than nanoparticles. The heatmap of the GSEA z-scores of the REACTOME gene sets that are enriched after BMDM treatment with NP, *L.mono*, or ΔHLY, compared to buffer-treated control. The z-scores in the heatmap were clustered without supervision. The light blue color represents an imputed value of z-scores equal zero and P-values >0.05, compared to Mock buffer treated controls.

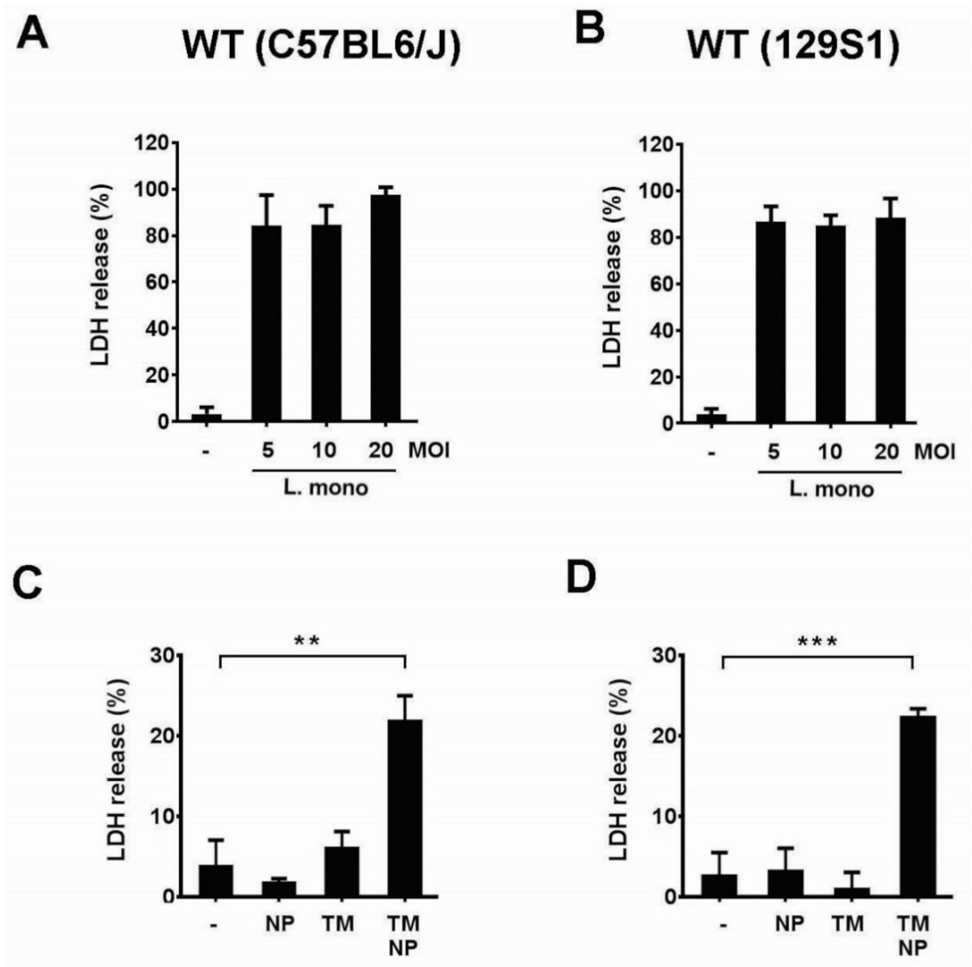

Fig. S5.
BMDM harvested from WT mice of C57BL6/J and 129S1 genetic backgrounds undergo necrotic death after exposure to *L.monocytogenes* (L.mono) or TM+NP treatment. (**A**) and (**B**) - BMDM cells from C57BL6/J or 129S1 mice were infected with *L.monocytogenes* at different MOI and cell death was assayed by LDH activity in the media, N=3. (**C**) and (**D**) BMDM cells from C57BL6/J or 129S1 mice were treated with TM and NP and cell death was assayed by LDH activity in the media, N=3. ** P=0.0025; *** P=0.0004.

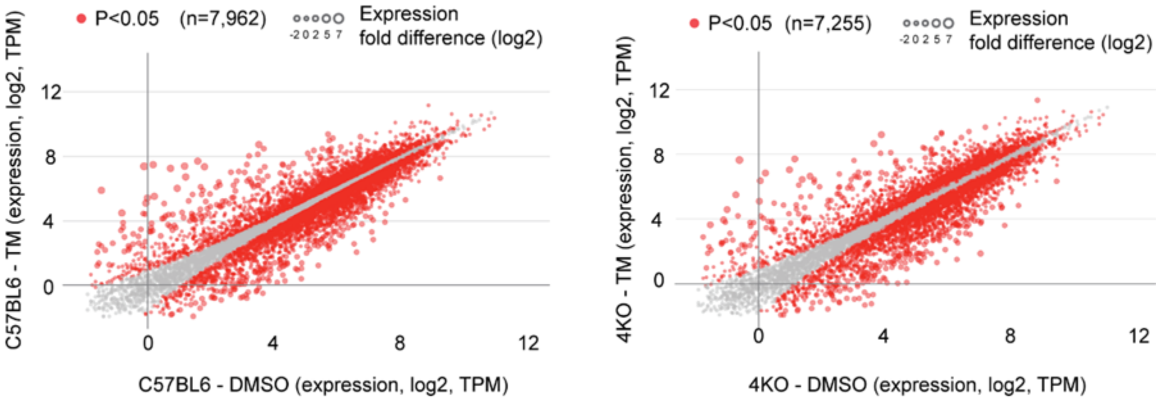

Fig. S6.

Treatment of cells with TM alone trigges global changes in gene expression in both WT and 4KO BMDM. Scatter plots of the expression values of genes in WT and 4KO BMDM treated with DMSO or TM. Genes differentially-expressed between these two treatment conditions with p <0.05, based on DESeq2 analysis, are shown in red. N=4 for each condition.

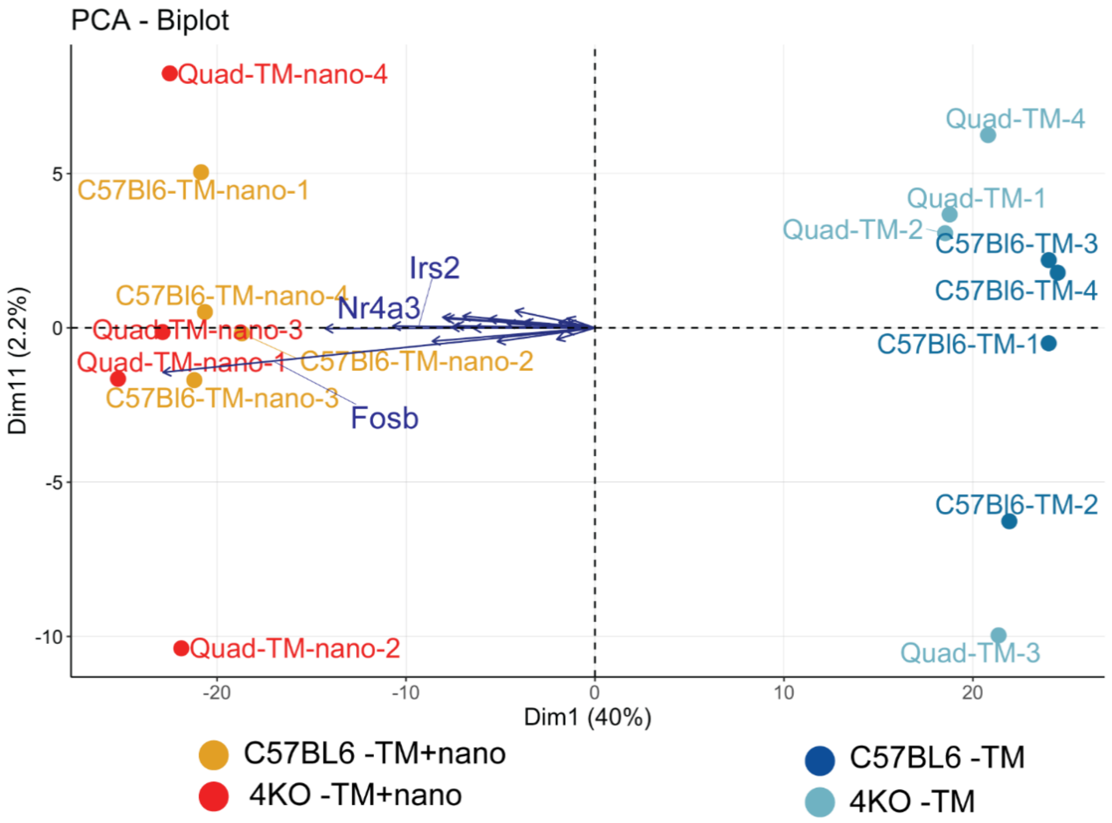

Fig. S7.

The distribution of transcriptomic signatures of BMDMs from WT and 4KO mice treated with TM or TM+NP on a principal component dimention plot. The imputation of as a single variable into the raw global transcriptomics data files for each sample allows for isolation of top genes contributing to the divergence of transcriptional phenotypes between TM+NP and TM-treated cells, and leads to defined sample distribution between these two treatment conditions along PCA Dimention-1 axis. The top 500 genes contributing to separation of samples on PC-1 axis are shown in Table S1. The 22 genes most contributing to sample separation along dimention 1 axis are shown with blue arrows. N=4 for each experimental condition.

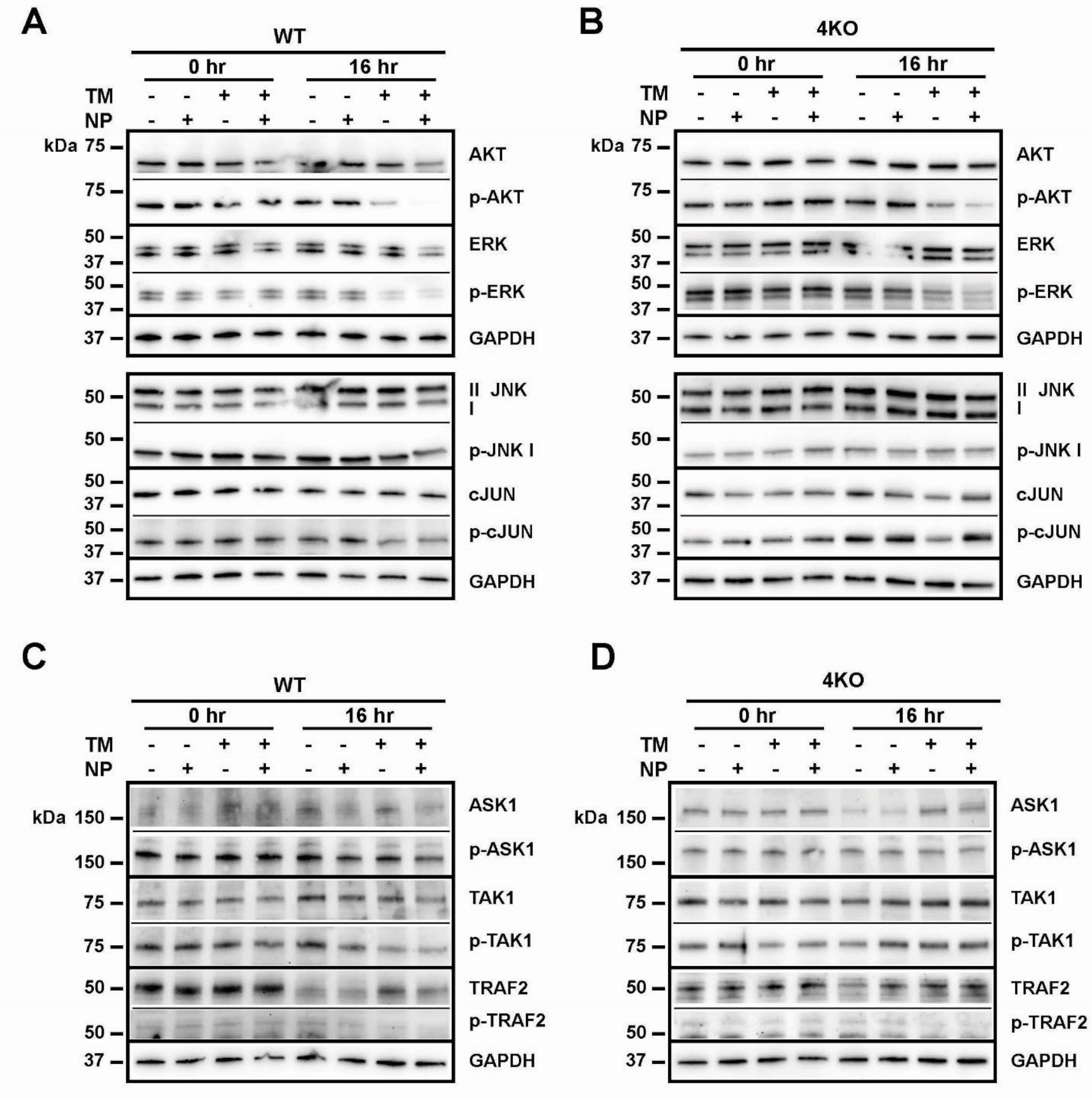

Fig. S8. Western blot analysis of activation of MAPK cascade signaling components at indicated times in WT **(A and C)** and 4KO **(B and D)** BMDMs after their treatment with DMSO (-), TM, NP, or TM and NP combination. N= 3.

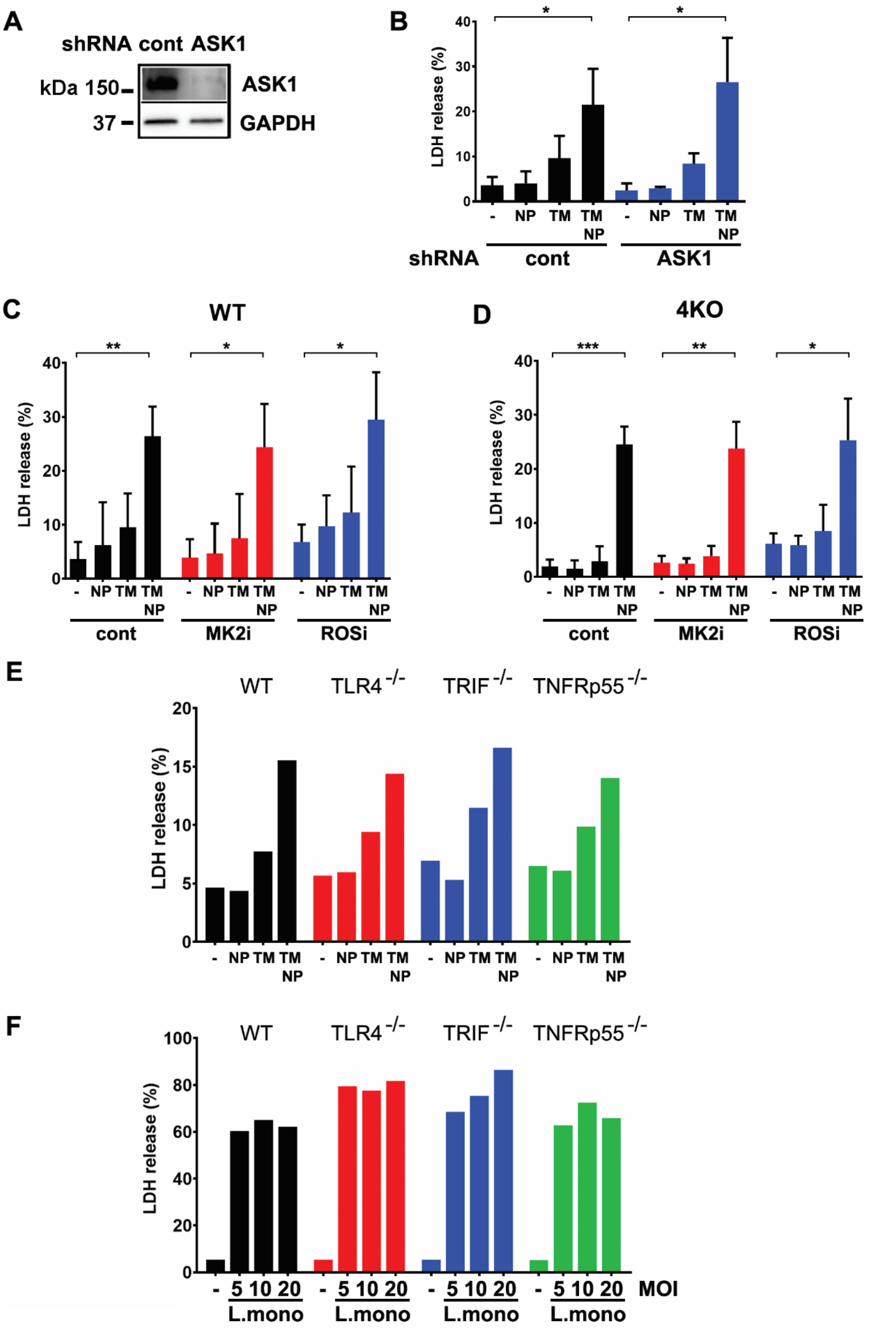

A
shRNA cont ASK1
kDa 150
37
ASK1
GAPDH
B
LDH release (%)
- NP TM TM NP - NP TM TM NP
shRNA cont ASK1
C
WT
LDH release (%)
- NP TM TM NP - NP TM TM NP - NP TM TM NP
cont MK2i ROSi
D
4KO
LDH release (%)
- NP TM TM NP - NP TM TM NP - NP TM TM NP
cont MK2i ROSi
E
WT TLR4 -/- TRIF -/- TNFRp55 -/-
LDH release (%)
- NP TM TM NP - NP TM TM NP - NP TM TM NP - NP TM TM NP
F
WT TLR4 -/- TRIF -/- TNFRp55 -/-
LDH release (%)
- 5 10 20 - 5 10 20 - 5 10 20 - 5 10 20 MOI
L.mono L.mono L.mono L.mono

Fig. S9.

(A) Expression of ASK1 was knocked down in BMDM from 4KO mice using lentiviral shRNA. ASK1 protein levels were determined by Western Blotting analysis. **(B)** Necrotic cell death was assessed by LDH activity release in the culture media, N=3. P= 0.0195 (control), P= 0.0137 (ASK1 shRNA). **(C)** Effects of chemicals targeting MK2 activity (MK2i) and ROS generation (ROSi) were assessed in BMDM from WT mice. LDH activity release in the culture media from different conditions was determined. N=3, P= 0.0034(cont); P= 0.0151(MK2i); P= 0.0137 (ROSi). **(D)** Same chemicals as in **(C)** were tested in 4KO BMDM, N=3, P= 0.0004 (cont); P= 0.0020 (MK2i); P= 0.0135 (ROSi). **(E)** BMDM cells from WT mice and mouse strains knockout for TLR4, TRIF, and TNFRp55 were treated with TM and NP and cell death was assayed by LDH activity release in the media. **(F)** BMDM cells from WT mice and mouse strains knockout for TLR4, TRIF, and TNFRp55 were infected with *L. monocytogenes* at different MOI and cell death was assayed by LDH activity in the media.

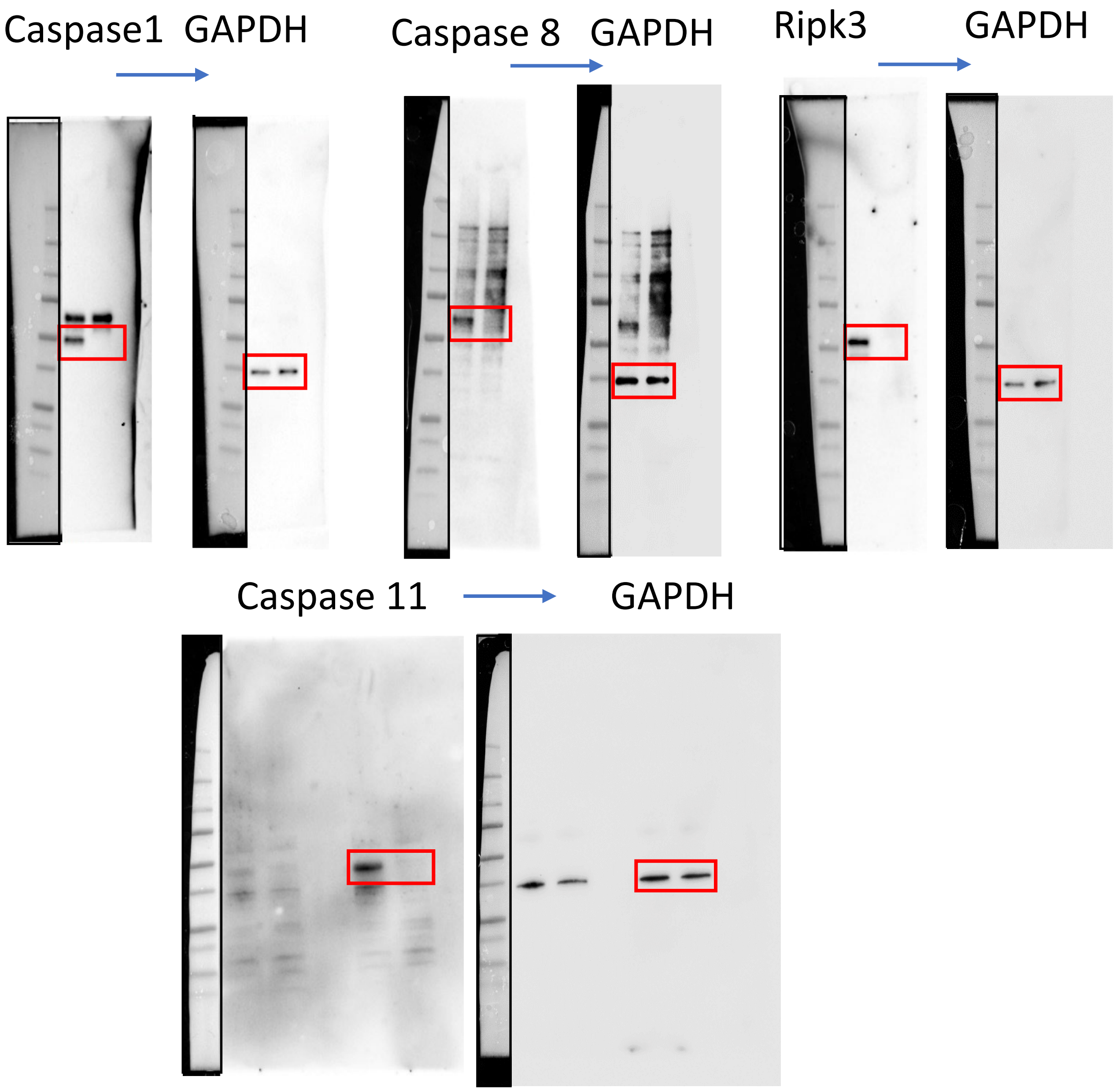

Fig. S10. The full-length images of the original Western blot membranes demonstrating expression of indicated proteins, shown in Fig. 1C in a cropped form. The areas of the membranes that were cropped and presented in Fig. 1C for each protein are depicted with red rectangles. The image of the molecular weight ladder loaded on the same gel was taken for each original membrane and was juxtaposed with the image of the same membrane after the Western blot processing. The border of the molecular weight ladder image is depicted by the black rectangle. The molecular weights corresponding to specific bands in the ladder are shown in Fig. 1C. Re-probing sequence for the same membrane is indicated with blue arrow.

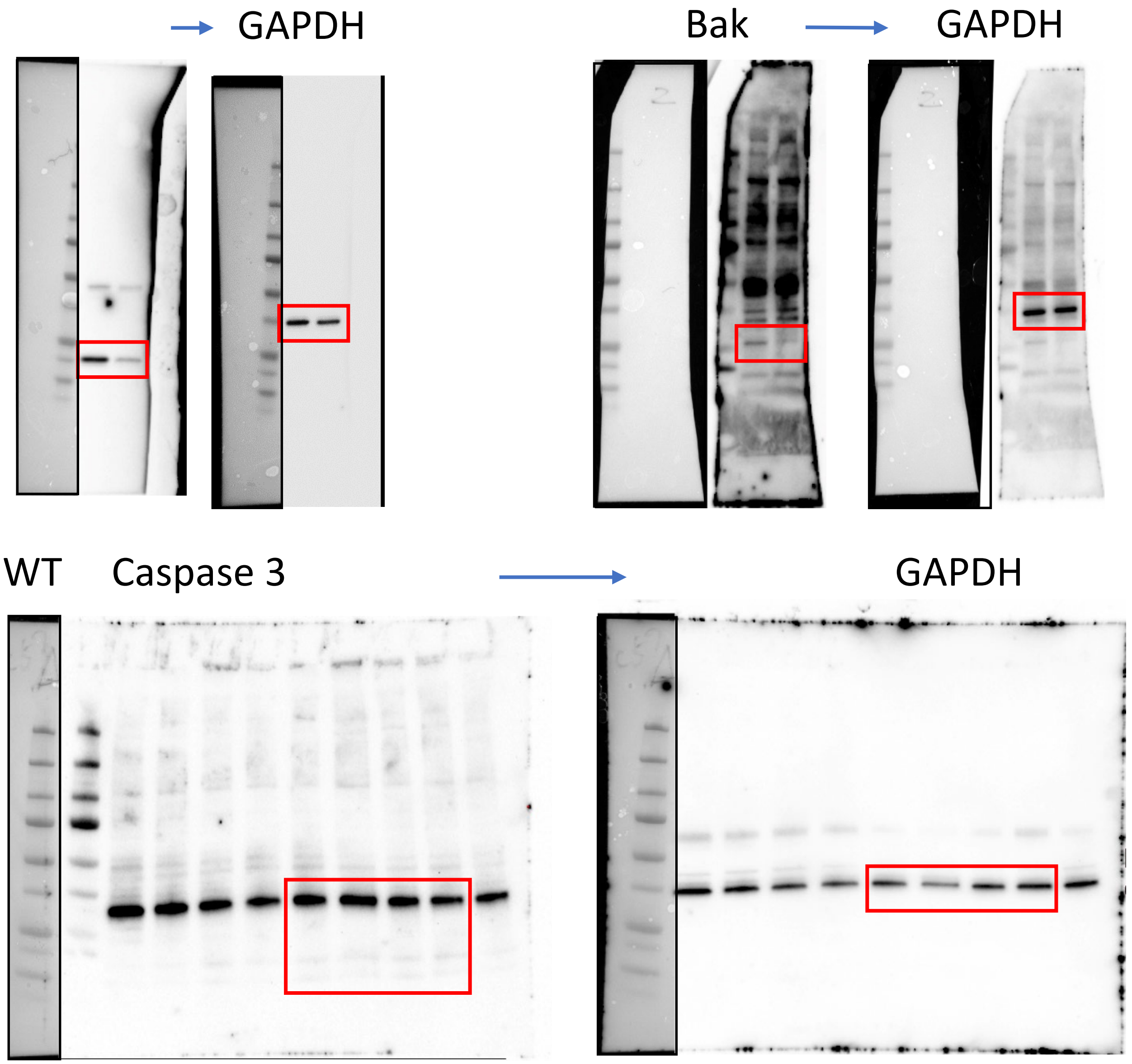

Fig. S11. The full-length images of the original Western blot membranes demonstrating expression of indicated proteins, shown in Fig. 2A and Fig. 2C in a cropped form. The areas of the membranes that were cropped and presented in Fig. 2A and Fig. 2C for each protein are depicted with red rectangles. The image of the molecular weight ladder loaded on the same gel was taken for each original membrane and was juxtaposed with the image of the same membrane after the Western blot processing. The border of the molecular weight ladder image is depicted by the black rectangle. The molecular weights corresponding to specific bands in the ladder are shown in Fig. 2A and Fig. 2C. Re-probing sequence for the same membrane is indicated with blue arrow.

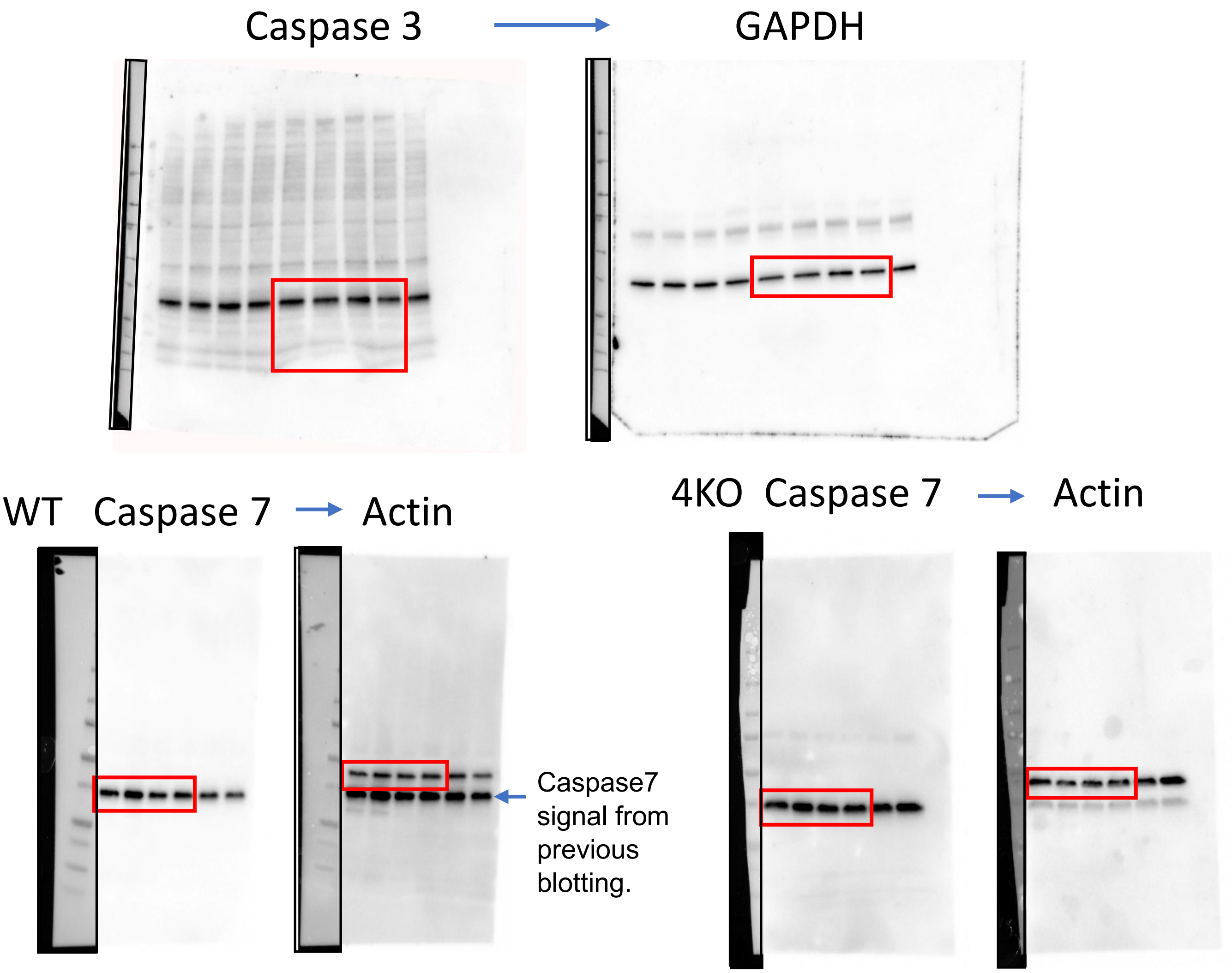

Fig. S12. The full-length images of the original Western blot membranes demonstrating expression of indicated proteins, shown in Fig. 2C and Fig. 2D in a cropped form. The areas of the membranes that were cropped and presented in Fig. 2C and Fig. 2D for each protein are depicted with red rectangles. The image of the molecular weight ladder loaded on the same gel was taken for each original membrane and was juxtaposed with the image of the same membrane after the Western blot processing. The border of the molecular weight ladder image is depicted by the black rectangle. The molecular weights corresponding to specific bands in the ladder are shown in Fig. 2C and Fig. 2D. Re-probing sequence for the same membrane is indicated with blue arrow.

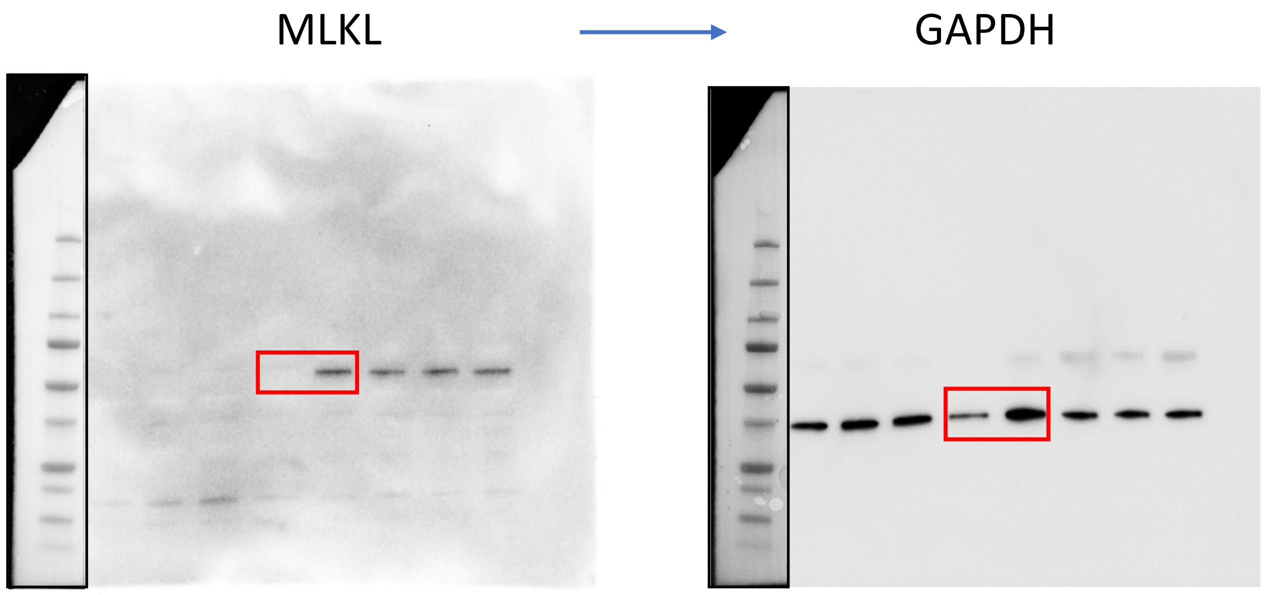

Fig. S13. The full-length images of the original Western blot membranes demonstrating expression of indicated proteins, shown in Fig. 2G in a cropped form. The areas of the membranes that were cropped and presented in Fig. 2G for each protein are depicted with red rectangles. The image of the molecular weight ladder loaded on the same gel was taken for each original membrane and was juxtaposed with the image of the same membrane after the Western blot processing. The border of the molecular weight ladder image is depicted by the black rectangle. The molecular weights corresponding to specific bands in the ladder are shown in Fig. 2G. Re-probing sequence for the same membrane is indicated with blue arrow.

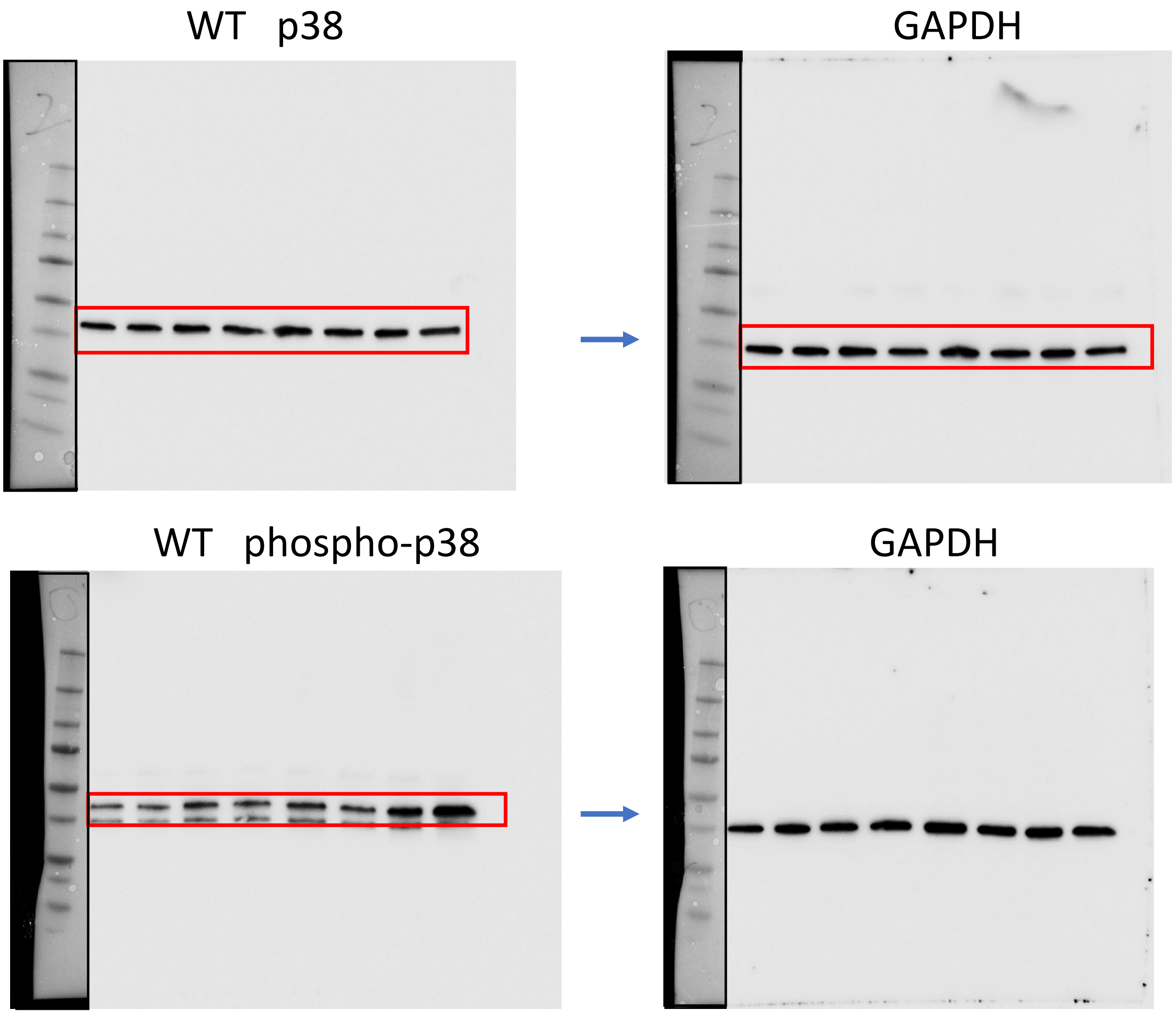

Fig. S14. The full-length images of the original Western blot membranes demonstrating expression of indicated proteins, shown in Fig. 4A in a cropped form. The areas of the membranes that were cropped and presented in Fig. 4A for each protein are depicted with red rectangles. The image of the molecular weight ladder loaded on the same gel was taken for each original membrane and was juxtaposed with the image of the same membrane after the Western blot processing. The border of the molecular weight ladder image is depicted by the black rectangle. The molecular weights corresponding to specific bands in the ladder are shown in Fig. 4A. Re-probing sequence for the same membrane is indicated with blue arrow.

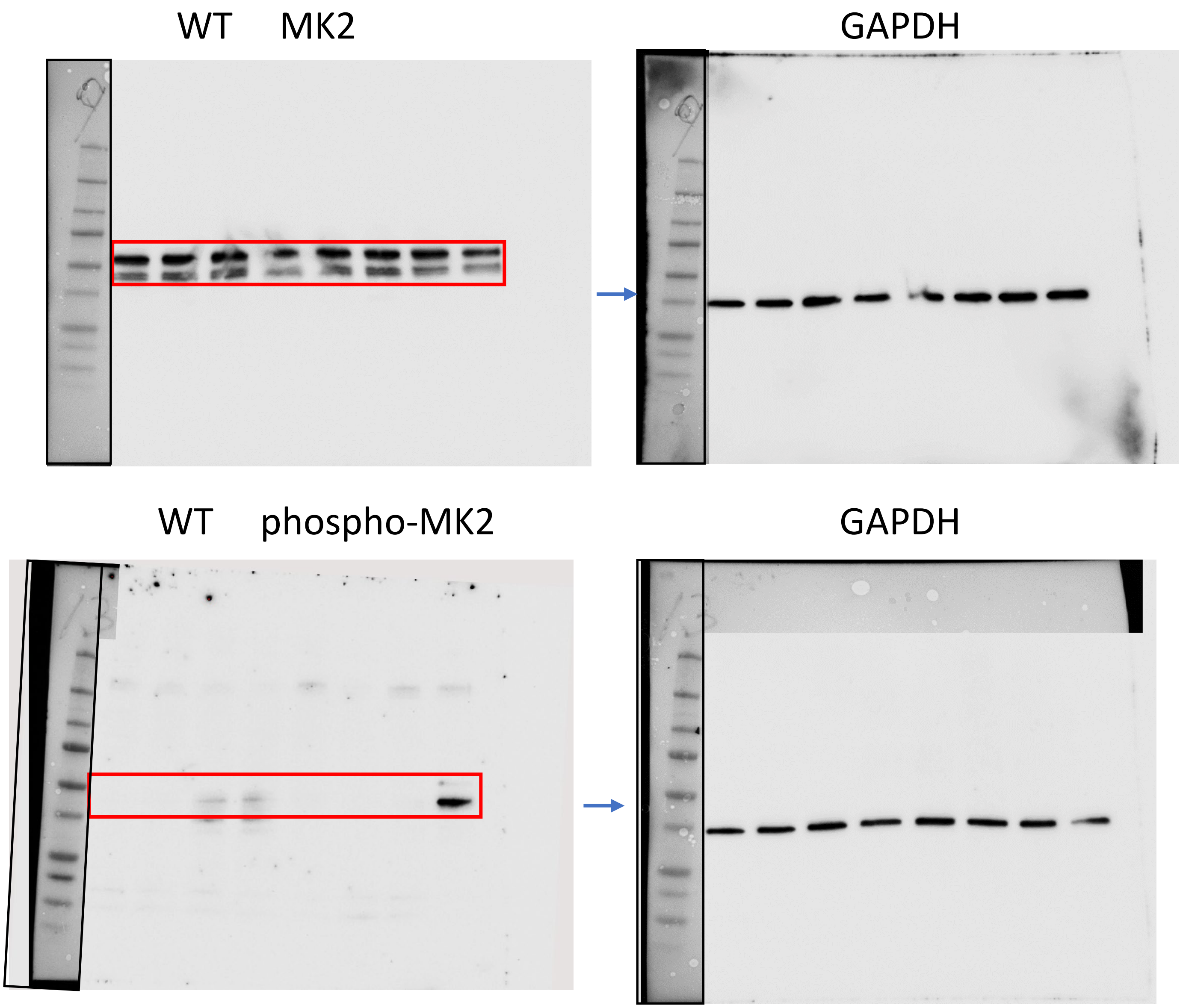

Fig. S15. The full-length images of the original Western blot membranes demonstrating expression of indicated proteins, shown in Fig. 4A in a cropped form. The areas of the membranes that were cropped and presented in Fig. 4A for each protein are depicted with red rectangles. The image of the molecular weight ladder loaded on the same gel was taken for each original membrane and was juxtaposed with the image of the same membrane after the Western blot processing. The border of the molecular weight ladder image is depicted by the black rectangle. The molecular weights corresponding to specific bands in the ladder are shown in Fig. 4A. Re-probing sequence for the same membrane is indicated with blue arrow.

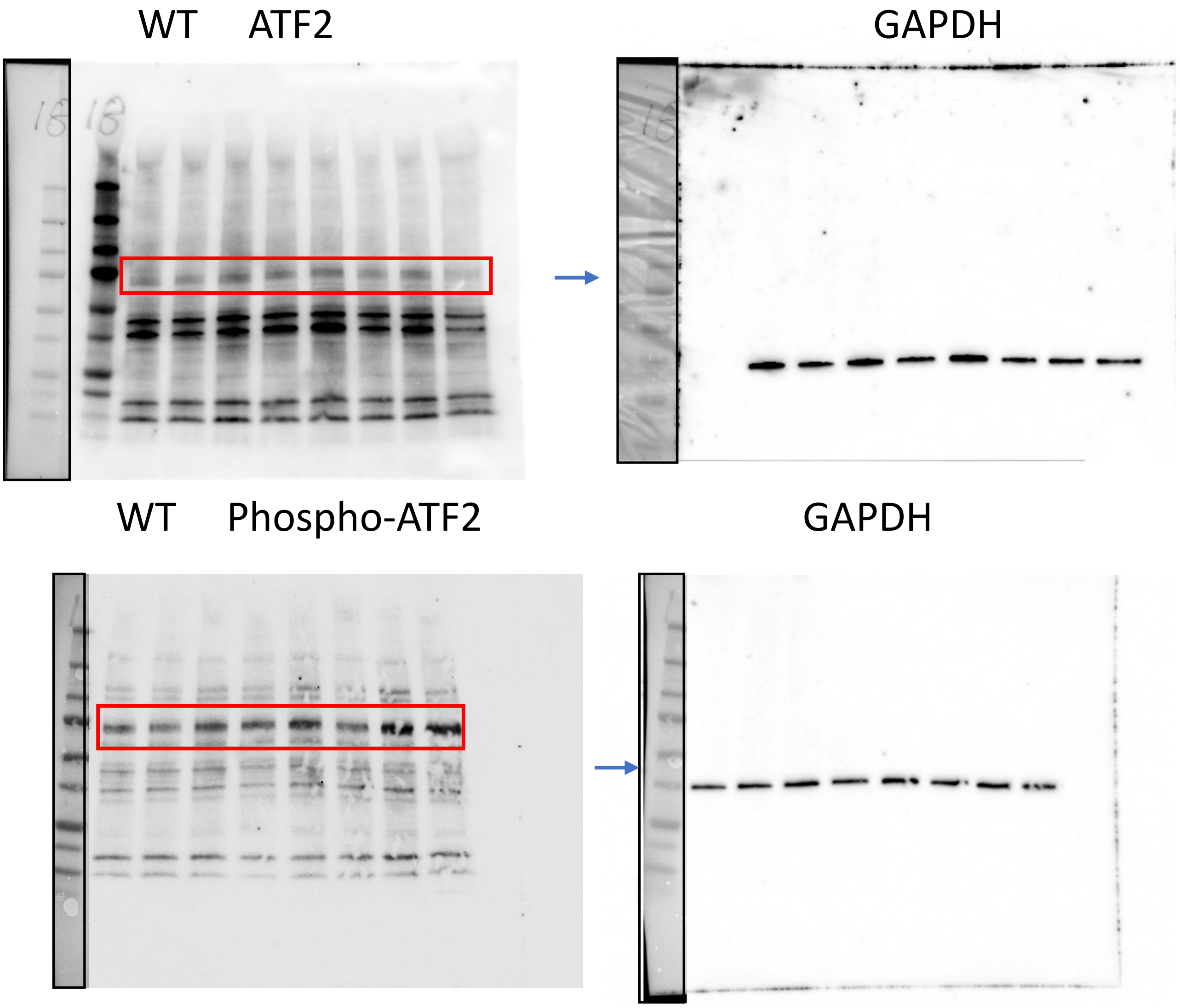

Fig. S16. The full-length images of the original Western blot membranes demonstrating expression of indicated proteins, shown in Fig. 4A in a cropped form. The areas of the membranes that were cropped and presented in Fig. 4A for each protein are depicted with red rectangles. The image of the molecular weight ladder loaded on the same gel was taken for each original membrane and was juxtaposed with the image of the same membrane after the Western blot processing. The border of the molecular weight ladder image is depicted by the black rectangle. The molecular weights corresponding to specific bands in the ladder are shown in Fig. 4A. Re-probing sequence for the same membrane is indicated with blue arrow.

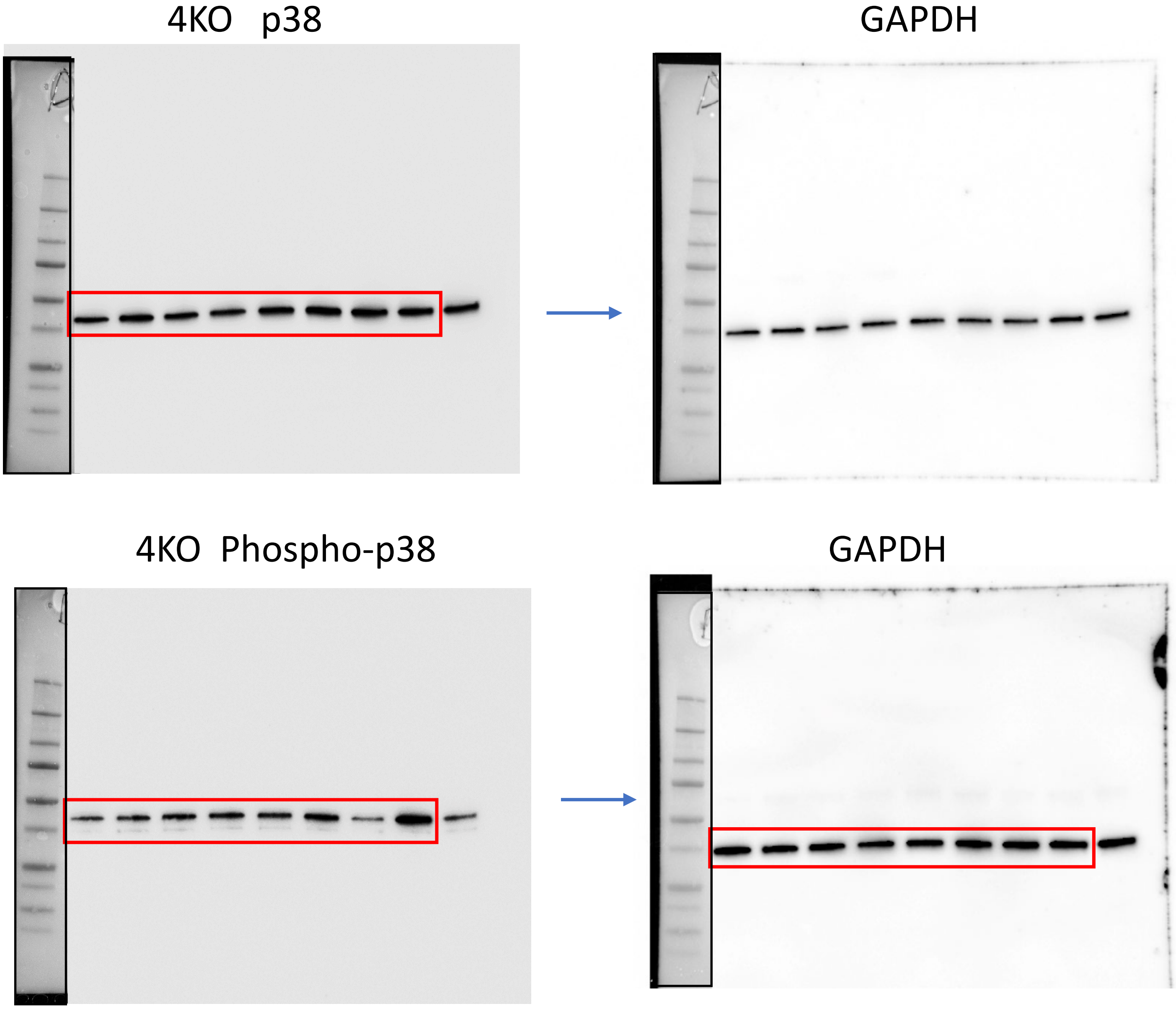

Fig. S17. The full-length images of the original Western blot membranes demonstrating expression of indicated proteins, shown in Fig. 4A in a cropped form. The areas of the membranes that were cropped and presented in Fig. 4A for each protein are depicted with red rectangles. The image of the molecular weight ladder loaded on the same gel was taken for each original membrane and was juxtaposed with the image of the same membrane after the Western blot processing. The border of the molecular weight ladder image is depicted by the black rectangle. The molecular weights corresponding to specific bands in the ladder are shown in Fig. 4A. Re-probing sequence for the same membrane is indicated with blue arrow.

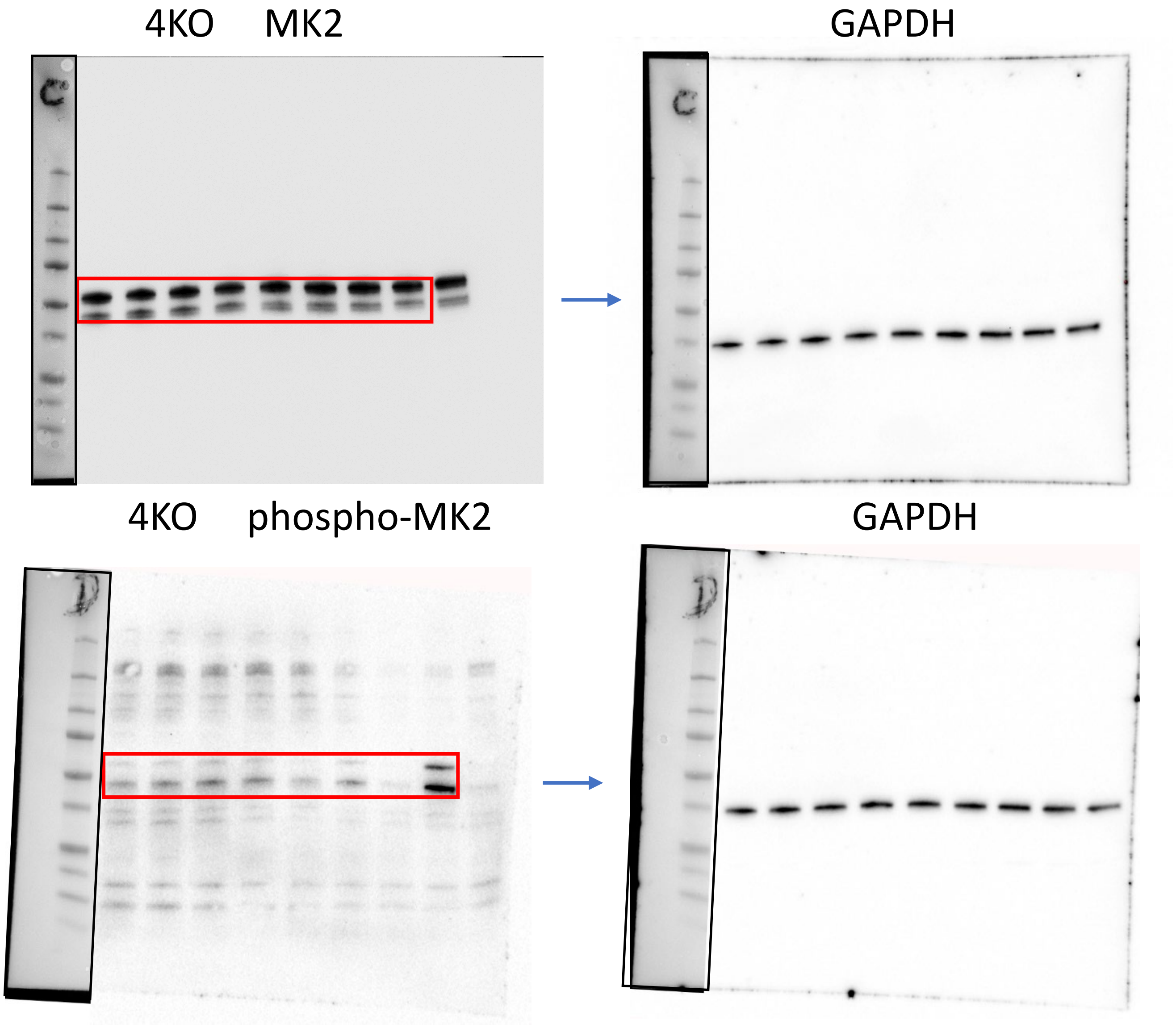

Fig. S18. The full-length images of the original Western blot membranes demonstrating expression of indicated proteins, shown in Fig. 4A in a cropped form. The areas of the membranes that were cropped and presented in Fig. 4A for each protein are depicted with red rectangles. The image of the molecular weight ladder loaded on the same gel was taken for each original membrane and was juxtaposed with the image of the same membrane after the Western blot processing. The border of the molecular weight ladder image is depicted by the black rectangle. The molecular weights corresponding to specific bands in the ladder are shown in Fig. 4A. Re-probing sequence for the same membrane is indicated with blue arrow.

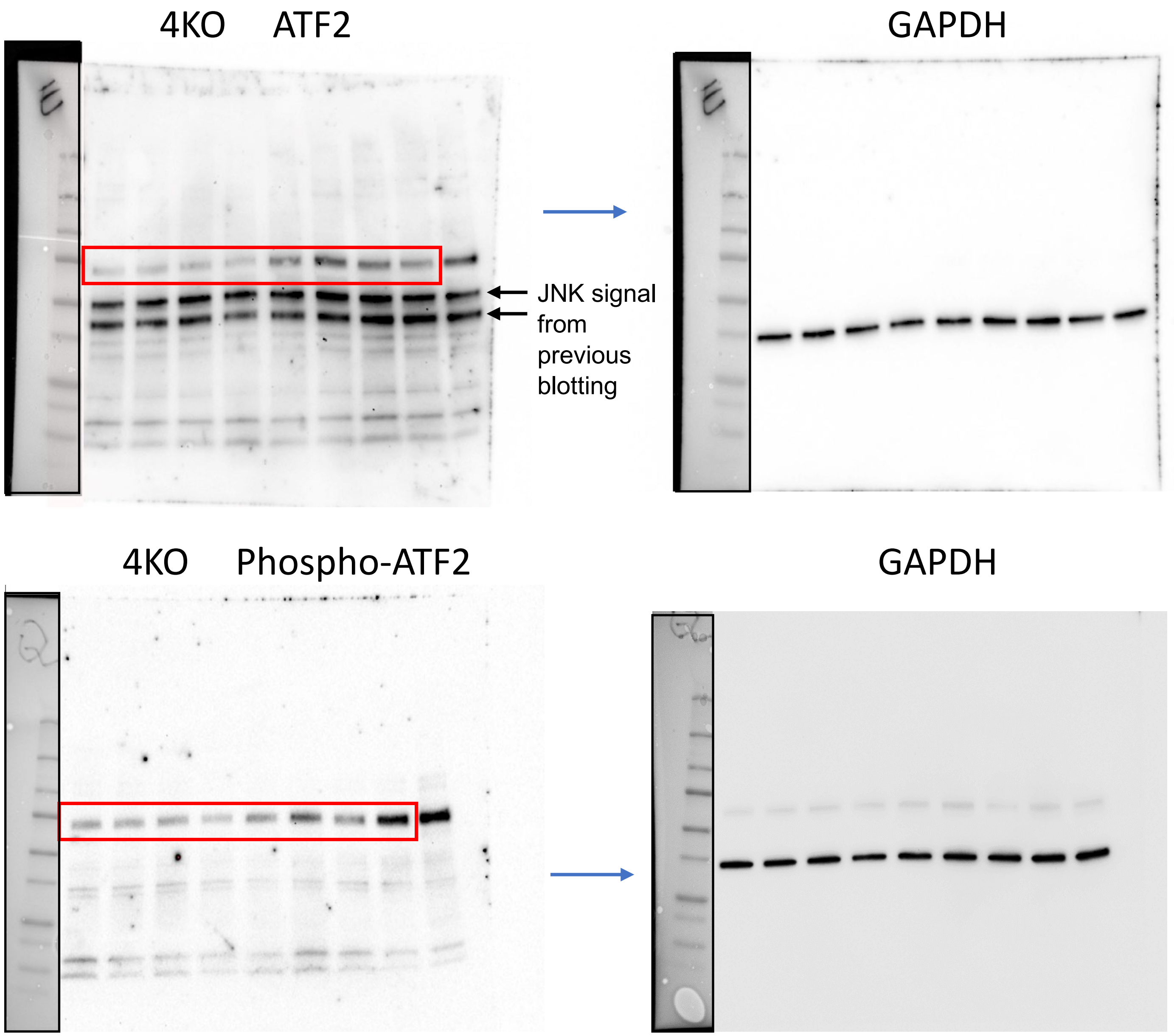

Fig. S19. The full-length images of the original Western blot membranes demonstrating expression of indicated proteins, shown in Fig. 4A in a cropped form. The areas of the membranes that were cropped and presented in Fig. 4A for each protein are depicted with red rectangles. The image of the molecular weight ladder loaded on the same gel was taken for each original membrane and was juxtaposed with the image of the same membrane after the Western blot processing. The border of the molecular weight ladder image is depicted by the black rectangle. The molecular weights corresponding to specific bands in the ladder are shown in Fig. 4A. Re-probing sequence for the same membrane is indicated with blue arrow.

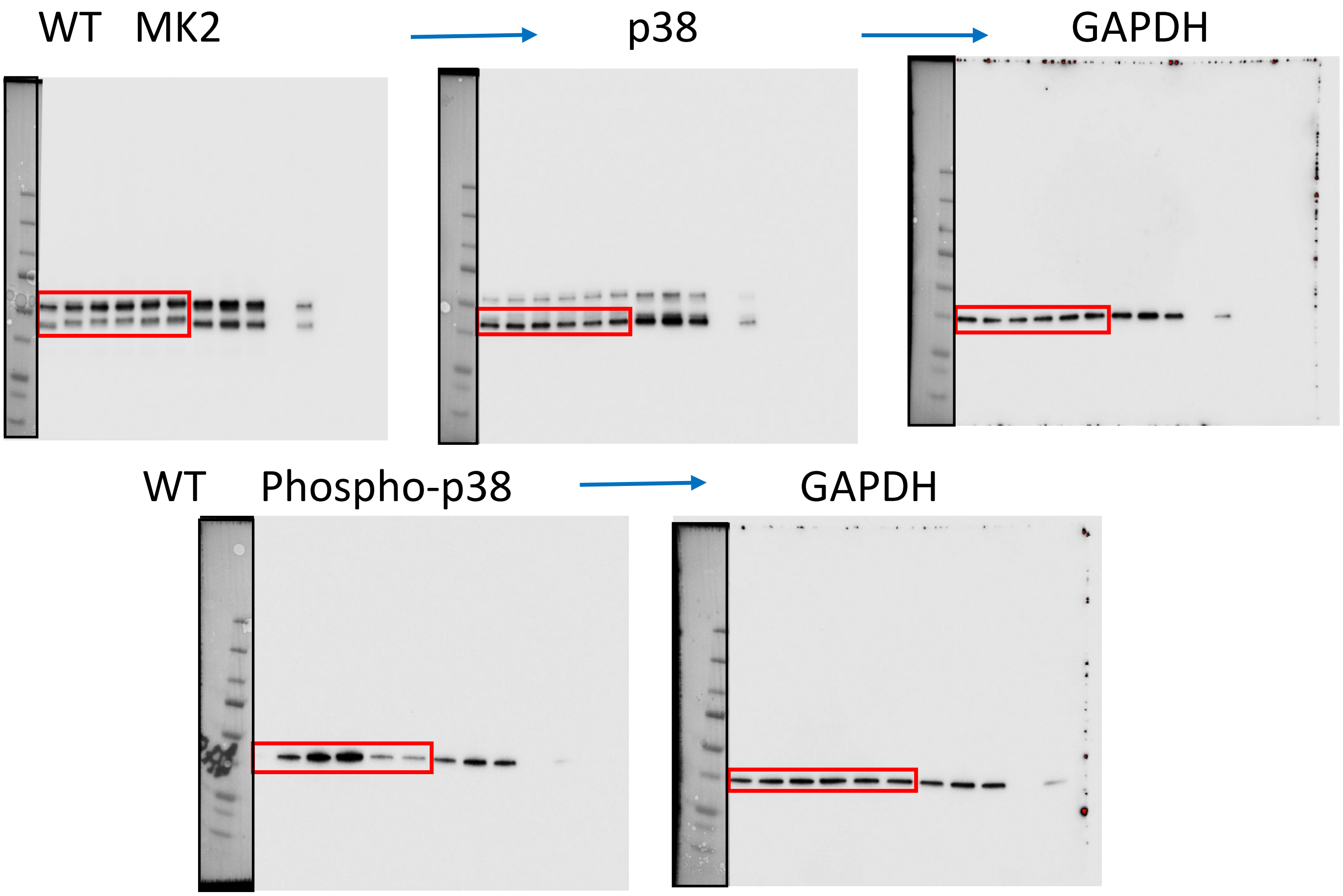

Fig. S20. The full-length images of the original Western blot membranes demonstrating expression of indicated proteins, shown in Fig. 4B in a cropped form. The areas of the membranes that were cropped and presented in Fig. 4B for each protein are depicted with red rectangles. The image of the molecular weight ladder loaded on the same gel was taken for each original membrane and was juxtaposed with the image of the same membrane after the Western blot processing. The border of the molecular weight ladder image is depicted by the black rectangle. The molecular weights corresponding to specific bands in the ladder are shown in Fig. 4B. Re-probing sequence for the same membrane is indicated with blue arrow.

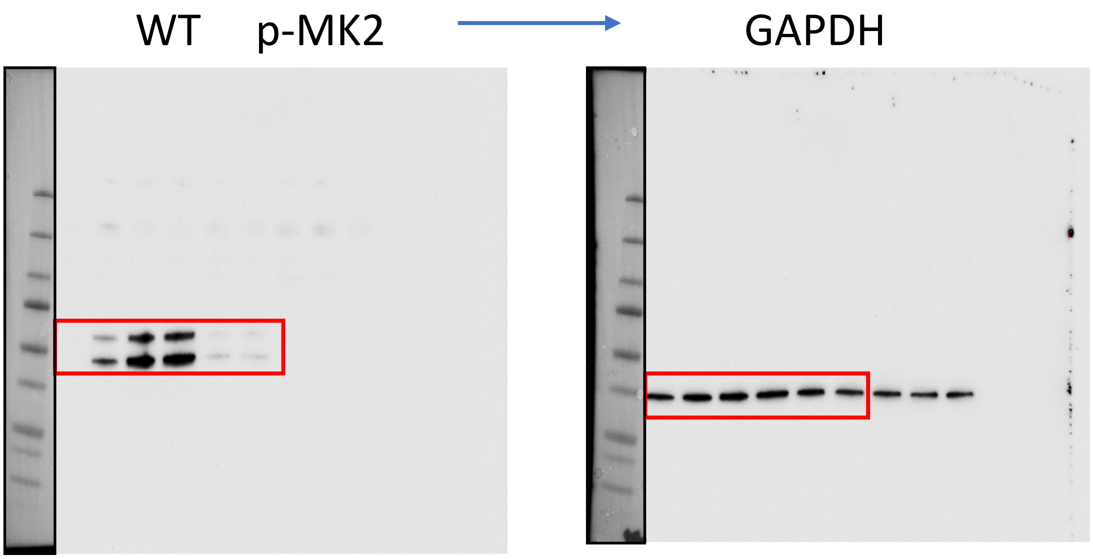

Fig. S21. The full-length images of the original Western blot membranes demonstrating expression of indicated proteins, shown in Fig. 4B in a cropped form. The areas of the membranes that were cropped and presented in Fig. 4B for each protein are depicted with red rectangles. The image of the molecular weight ladder loaded on the same gel was taken for each original membrane and was juxtaposed with the image of the same membrane after the Western blot processing. The border of the molecular weight ladder image is depicted by the black rectangle. The molecular weights corresponding to specific bands in the ladder are shown in Fig. 4B. Re-probing sequence for the same membrane is indicated with blue arrow.

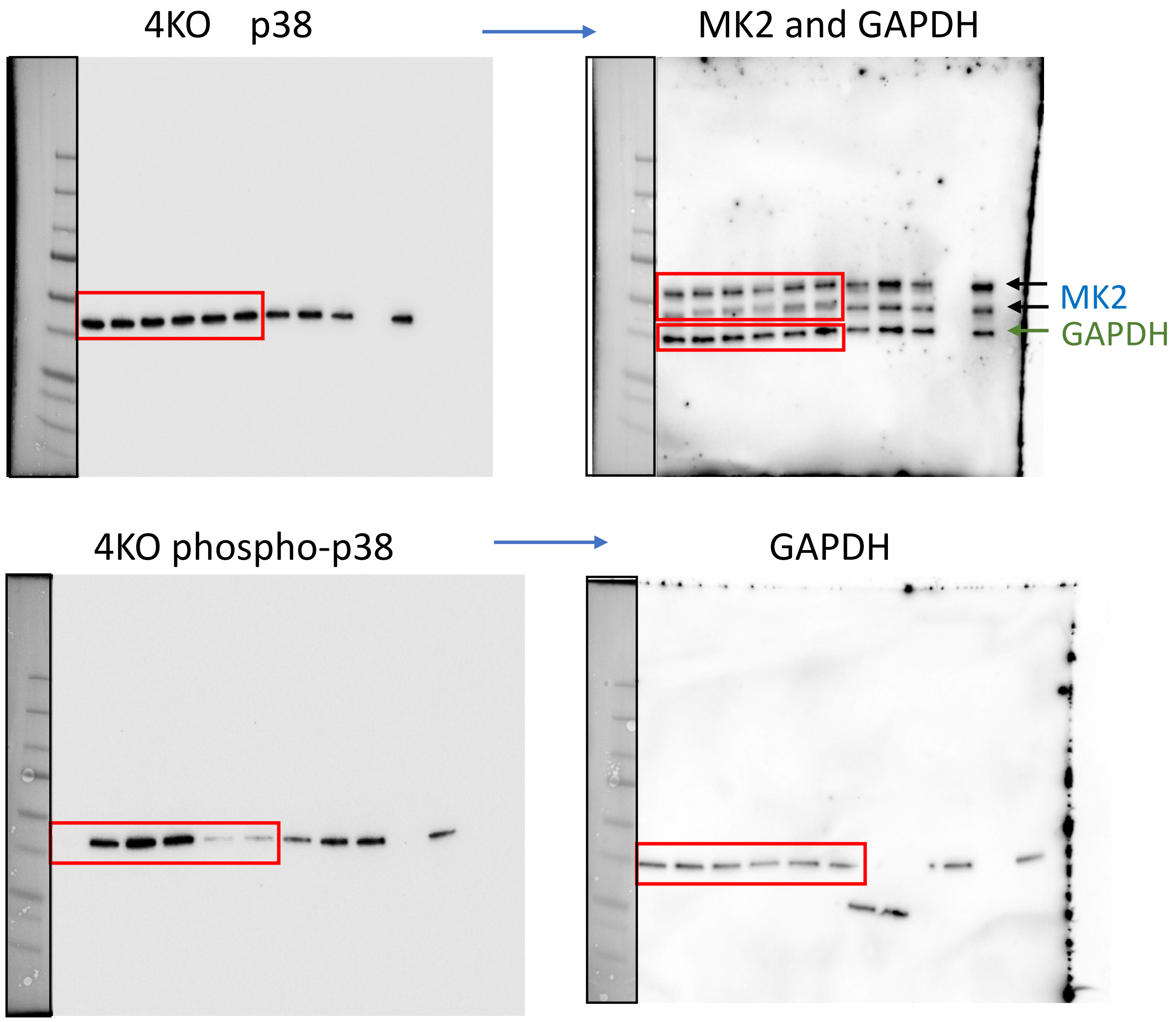

Fig. S22. The full-length images of the original Western blot membranes demonstrating expression of indicated proteins, shown in Fig. 4B in a cropped form. The areas of the membranes that were cropped and presented in Fig. 4B for each protein are depicted with red rectangles. The image of the molecular weight ladder loaded on the same gel was taken for each original membrane and was juxtaposed with the image of the same membrane after the Western blot processing. The border of the molecular weight ladder image is depicted by the black rectangle. The molecular weights corresponding to specific bands in the ladder are shown in Fig. 4B. Re-probing sequence for the same membrane is indicated with blue arrow.

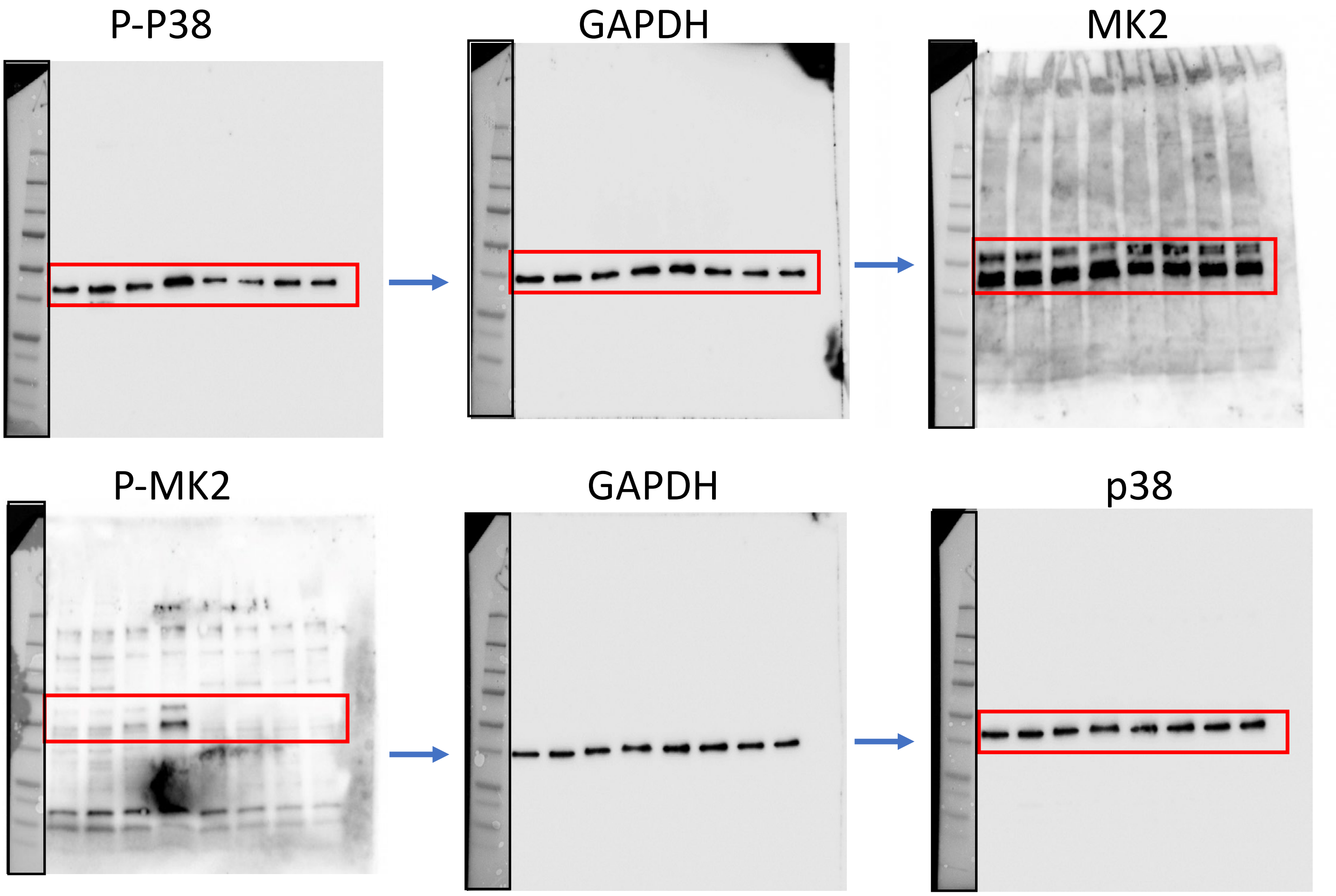

Fig. S23. The full-length images of the original Western blot membranes demonstrating expression of indicated proteins, shown in Fig. 5A in a cropped form. The areas of the membranes that were cropped and presented in Fig. 5A for each protein are depicted with red rectangles. The image of the molecular weight ladder loaded on the same gel was taken for each original membrane and was juxtaposed with the image of the same membrane after the Western blot processing. The border of the molecular weight ladder image is depicted by the black rectangle. The molecular weights corresponding to specific bands in the ladder are shown in Fig. 5A. Re-probing sequence for the same membrane is indicated with blue arrow.

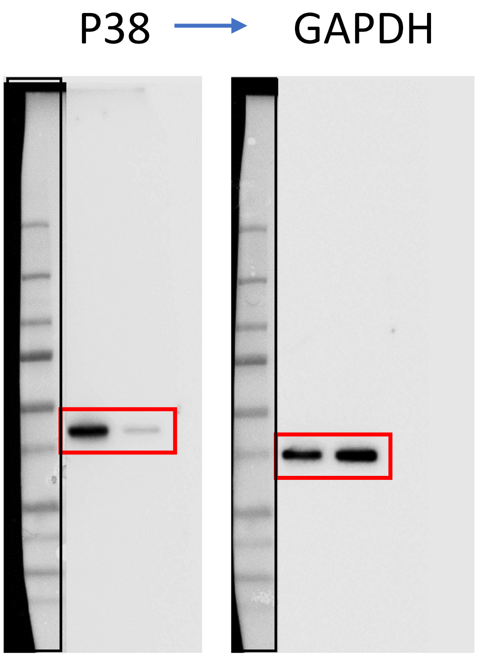

Fig. S24. The full-length images of the original Western blot membranes demonstrating expression of indicated proteins, shown in Fig. 5C in a cropped form. The areas of the membranes that were cropped and presented in Fig. 5C for each protein are depicted with red rectangles. The image of the molecular weight ladder loaded on the same gel was taken for each original membrane and was juxtaposed with the image of the same membrane after the Western blot processing. The border of the molecular weight ladder image is depicted by the black rectangle. The molecular weights corresponding to specific bands in the ladder are shown in Fig. 5C. Re-probing sequence for the same membrane is indicated with blue arrow.

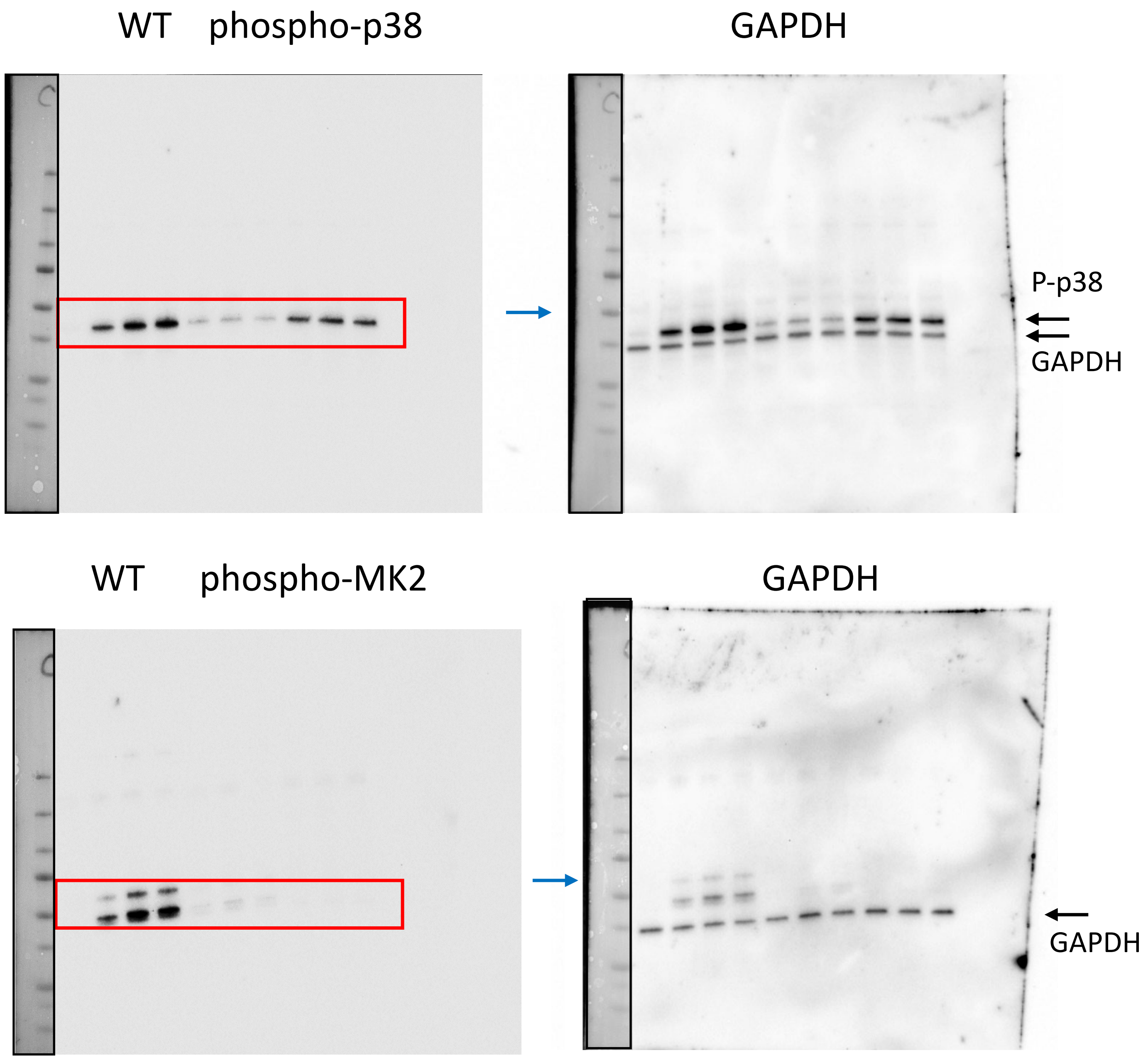

Fig. S25. The full-length images of the original Western blot membranes demonstrating expression of indicated proteins, shown in Fig. 5E in a cropped form. The areas of the membranes that were cropped and presented in Fig. 5E for each protein are depicted with red rectangles. The image of the molecular weight ladder loaded on the same gel was taken for each original membrane and was juxtaposed with the image of the same membrane after the Western blot processing. The border of the molecular weight ladder image is depicted by the black rectangle. The molecular weights corresponding to specific bands in the ladder are shown in Fig. 5E. Re-probing sequence for the same membrane is indicated with blue arrow.

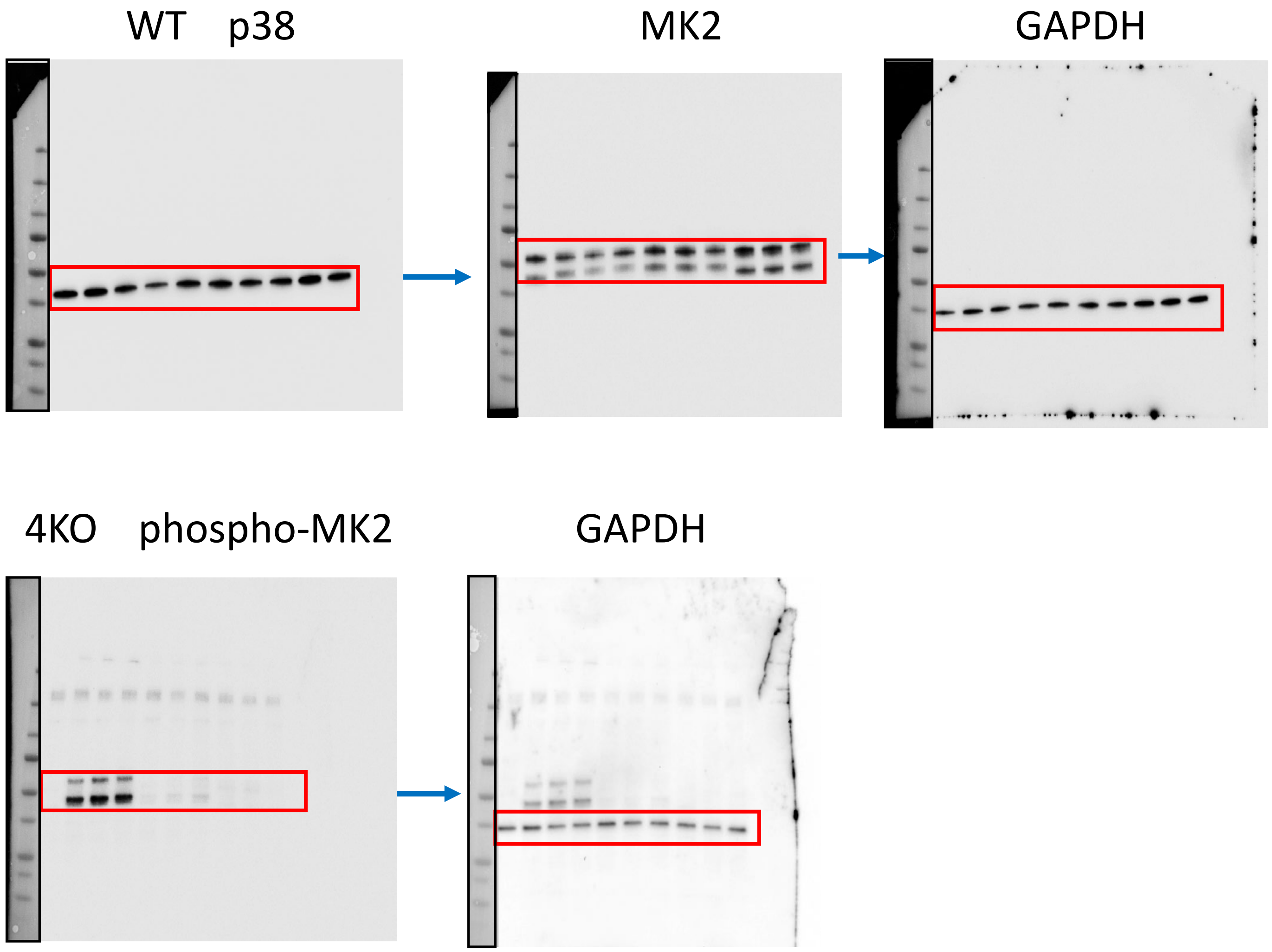

Fig. S26. The full-length images of the original Western blot membranes demonstrating expression of indicated proteins, shown in Fig. 5E in a cropped form. The areas of the membranes that were cropped and presented in Fig. 5E for each protein are depicted with red rectangles. The image of the molecular weight ladder loaded on the same gel was taken for each original membrane and was juxtaposed with the image of the same membrane after the Western blot processing. The border of the molecular weight ladder image is depicted by the black rectangle. The molecular weights corresponding to specific bands in the ladder are shown in Fig. 5E. Re-probing sequence for the same membrane is indicated with blue arrow.

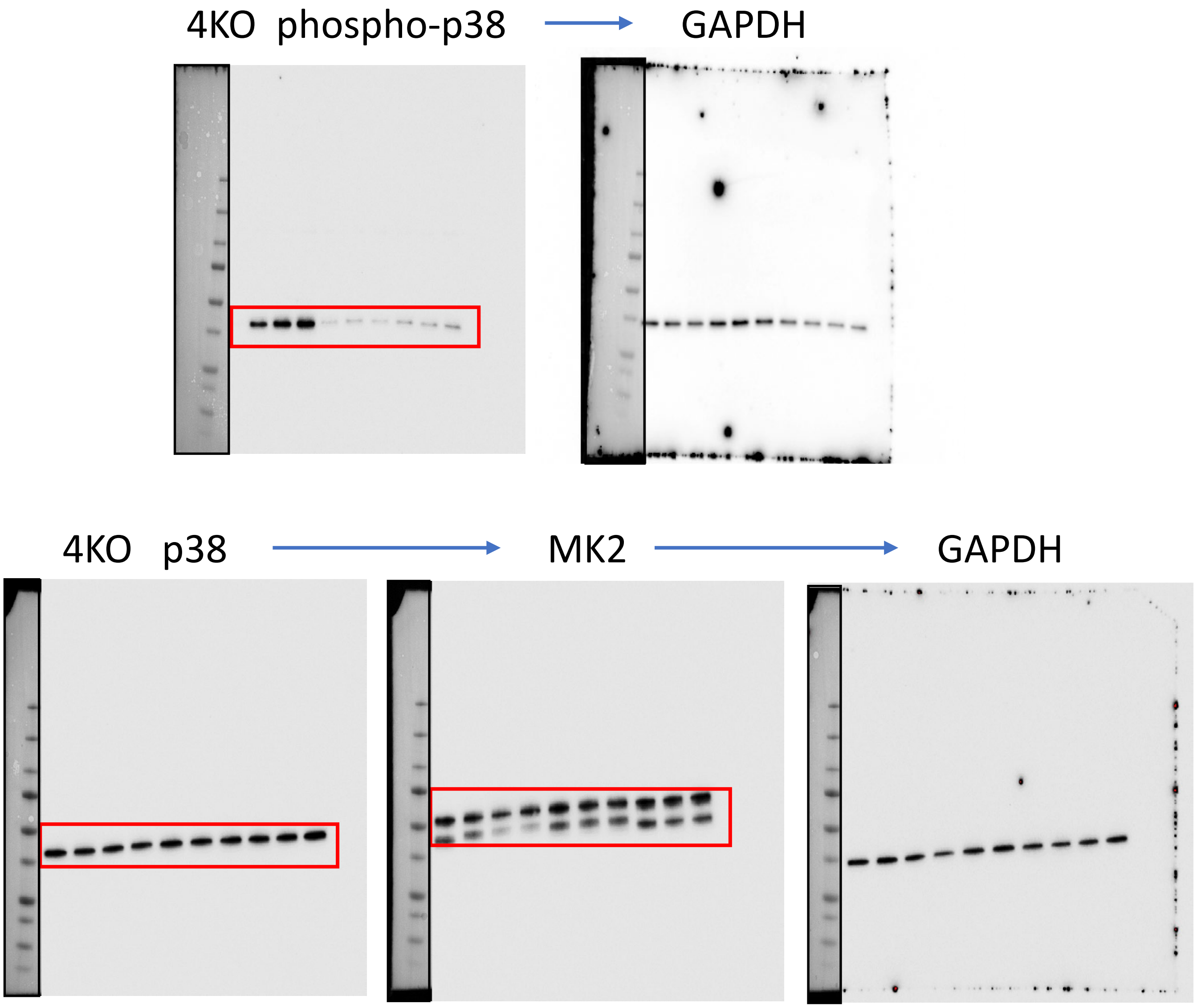

Fig. S27. The full-length images of the original Western blot membranes demonstrating expression of indicated proteins, shown in Fig. 5E in a cropped form. The areas of the membranes that were cropped and presented in Fig. 5E for each protein are depicted with red rectangles. The image of the molecular weight ladder loaded on the same gel was taken for each original membrane and was juxtaposed with the image of the same membrane after the Western blot processing. The border of the molecular weight ladder image is depicted by the black rectangle. The molecular weights corresponding to specific bands in the ladder are shown in Fig. 5E. Re-probing sequence for the same membrane is indicated with blue arrow.

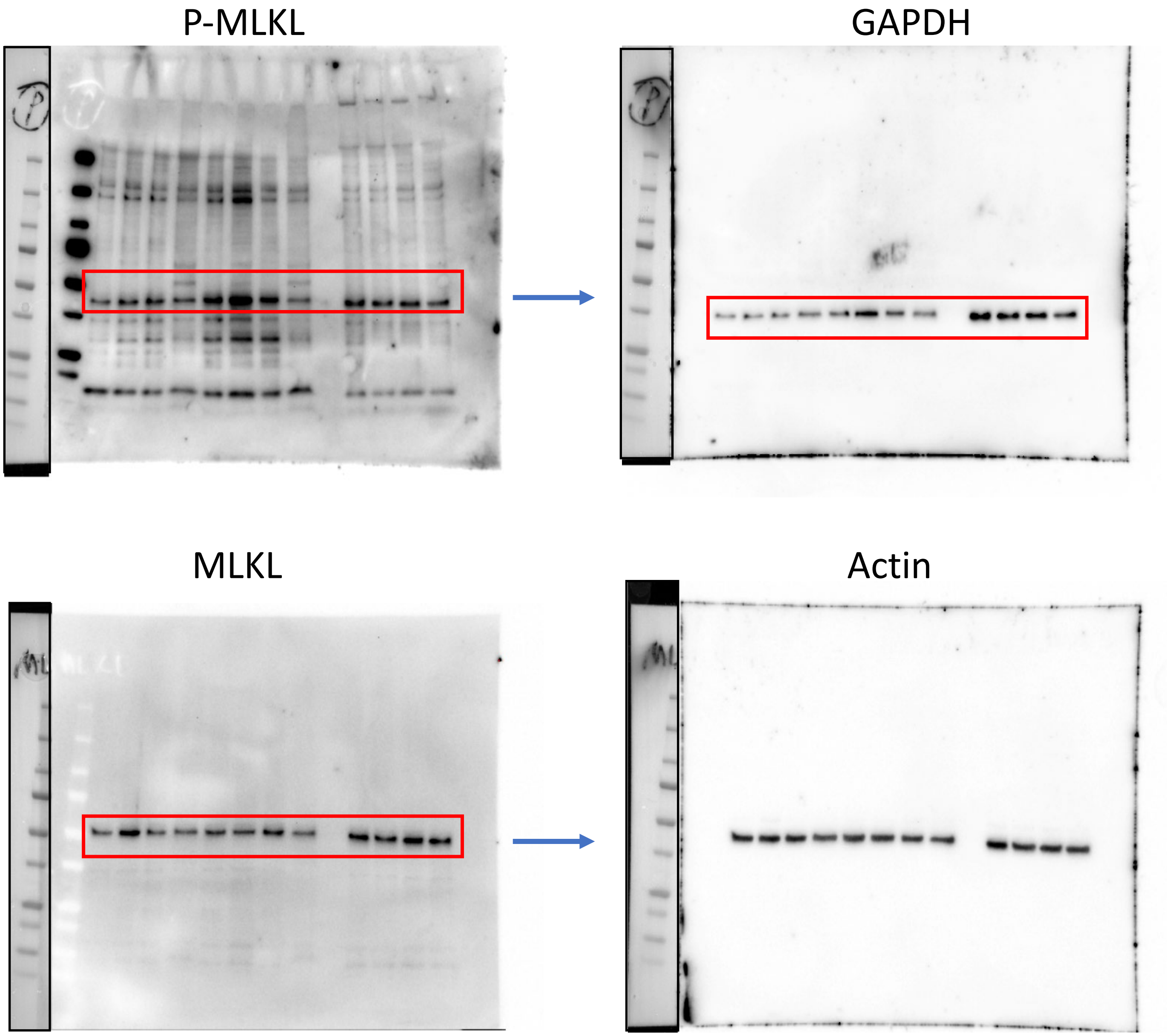

Fig. S28. The full-length images of the original Western blot membranes demonstrating expression of indicated proteins, shown in Fig. 6B in a cropped form. The areas of the membranes that were cropped and presented in Fig. 6B for each protein are depicted with red rectangles. The image of the molecular weight ladder loaded on the same gel was taken for each original membrane and was juxtaposed with the image of the same membrane after the Western blot processing. The border of the molecular weight ladder image is depicted by the black rectangle. The molecular weights corresponding to specific bands in the ladder are shown in Fig. 6B. Re-probing sequence for the same membrane is indicated with blue arrow.

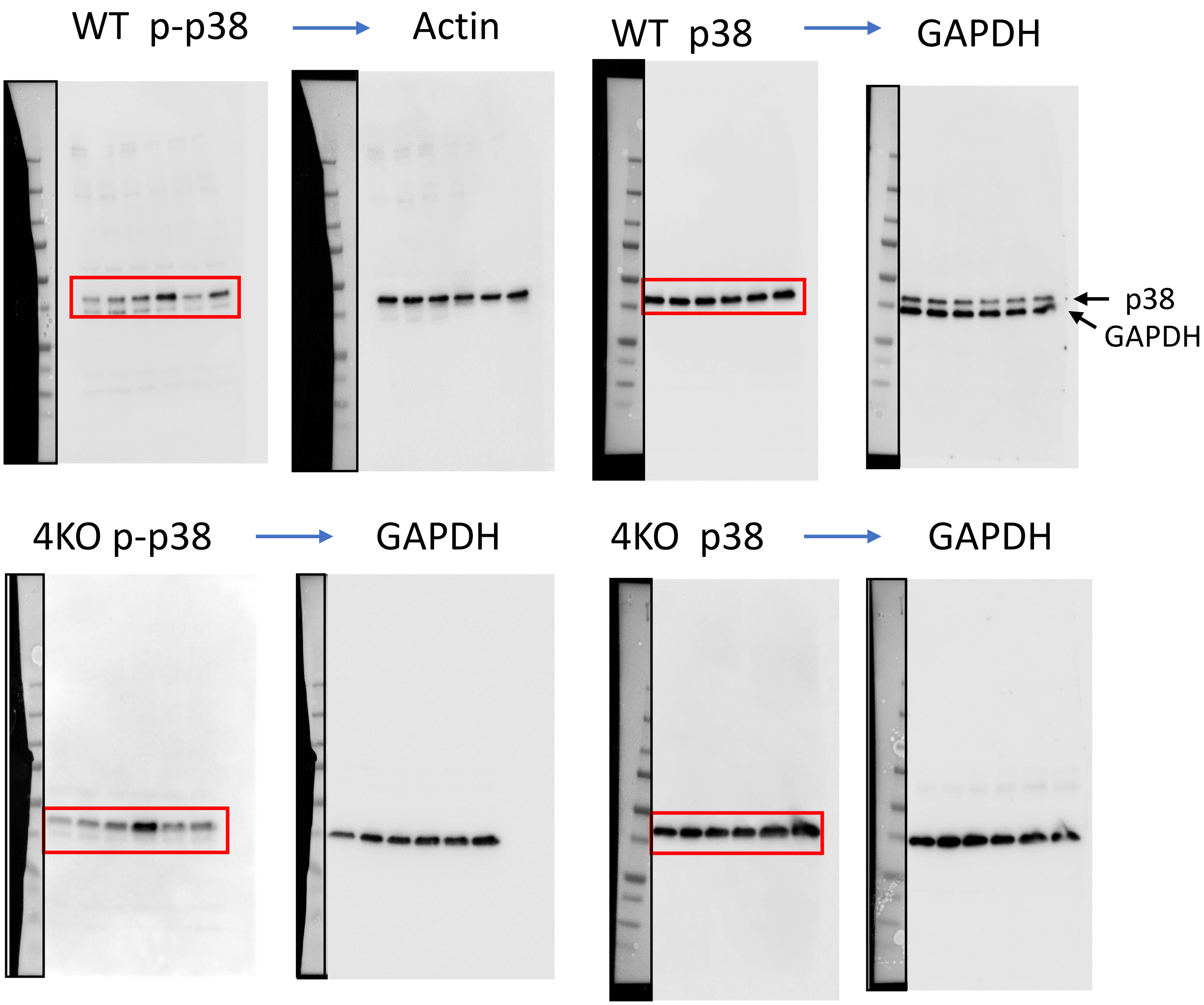

Fig. S29. The full-length images of the original Western blot membranes demonstrating expression of indicated proteins, shown in Fig. 6E in a cropped form. The areas of the membranes that were cropped and presented in Fig. 6E for each protein are depicted with red rectangles. The image of the molecular weight ladder loaded on the same gel was taken for each original membrane and was juxtaposed with the image of the same membrane after the Western blot processing. The border of the molecular weight ladder image is depicted by the black rectangle. The molecular weights corresponding to specific bands in the ladder are shown in Fig. 6E. Re-probing sequence for the same membrane is indicated with blue arrow.

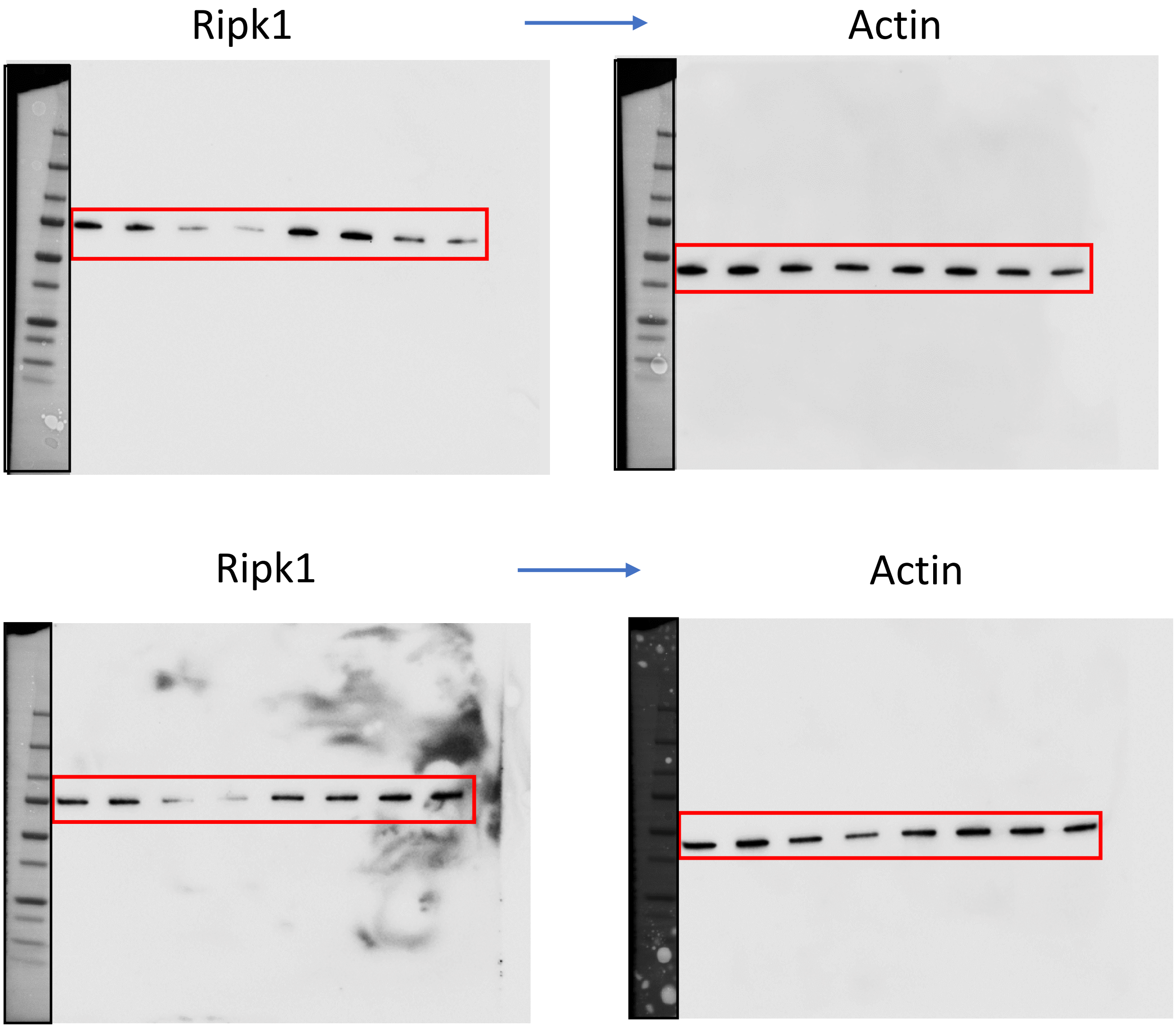

Fig. S30. The full-length images of the original Western blot membranes demonstrating expression of indicated proteins, shown in Fig. 6I and Fig. 6J in a cropped form. The areas of the membranes that were cropped and presented in Fig. 6I and Fig. 6J for each protein are depicted with red rectangles. The image of the molecular weight ladder loaded on the same gel was taken for each original membrane and was juxtaposed with the image of the same membrane after the Western blot processing. The border of the molecular weight ladder image is depicted by the black rectangle. The molecular weights corresponding to specific bands in the ladder are shown in Fig. 6I and Fig. 6J. Re-probing sequence for the same membrane is indicated with blue arrow.

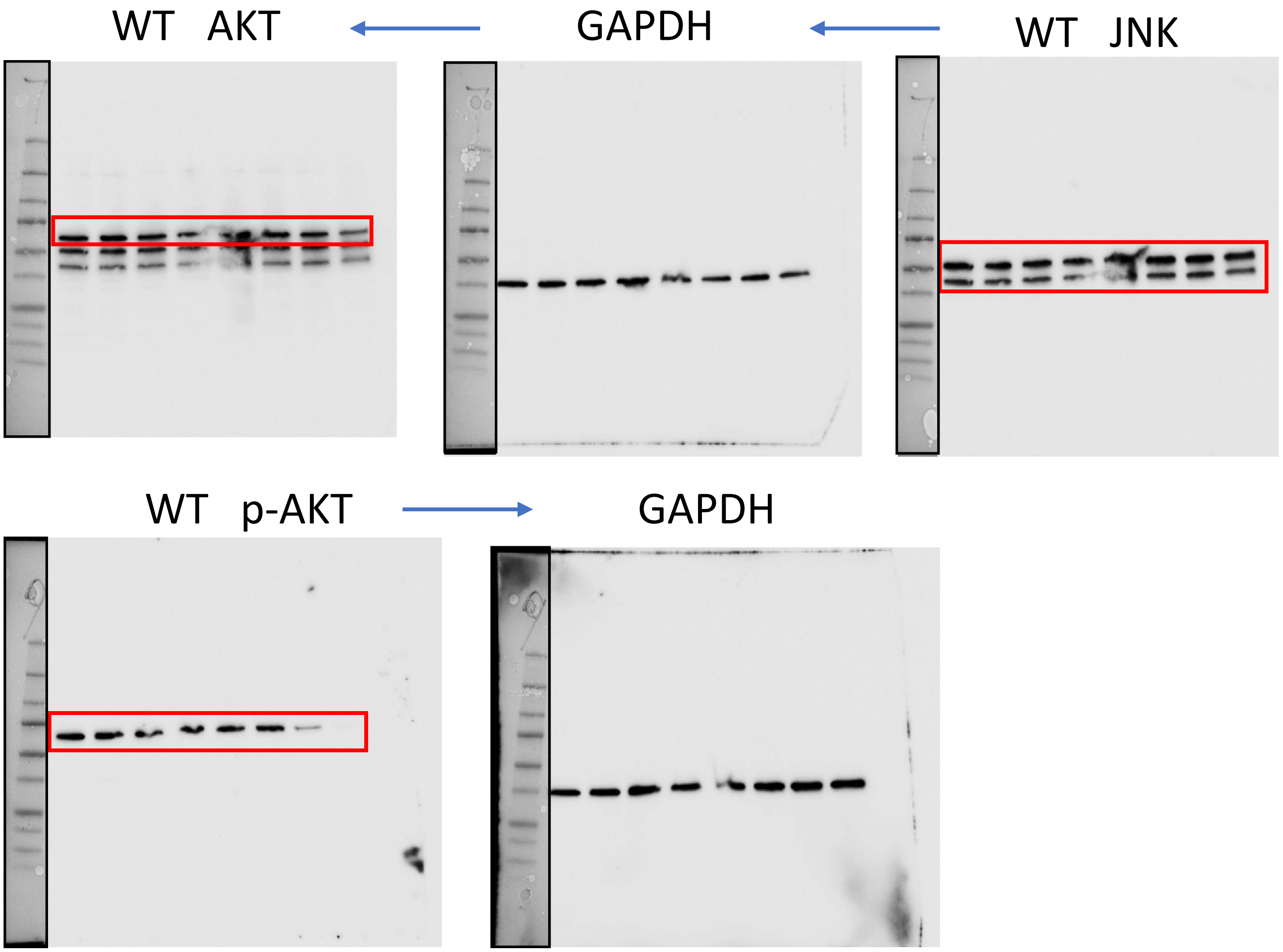

Fig. S31. The full-length images of the original Western blot membranes demonstrating expression of indicated proteins, shown in Supplemental Fig. S8A in a cropped form. The areas of the membranes that were cropped and presented in Supplemental Fig. S8A for each protein are depicted with red rectangles. The image of the molecular weight ladder loaded on the same gel was taken for each original membrane and was juxtaposed with the image of the same membrane after the Western blot processing. The border of the molecular weight ladder image is depicted by the black rectangle. The molecular weights corresponding to specific bands in the ladder are shown in Supplemental Fig. S8A. Re-probing sequence for the same membrane is indicated with blue arrow. The blotting order for p-AKT membrane was P-AKT, GAPDH, and then MK2 for Fig. 4A.

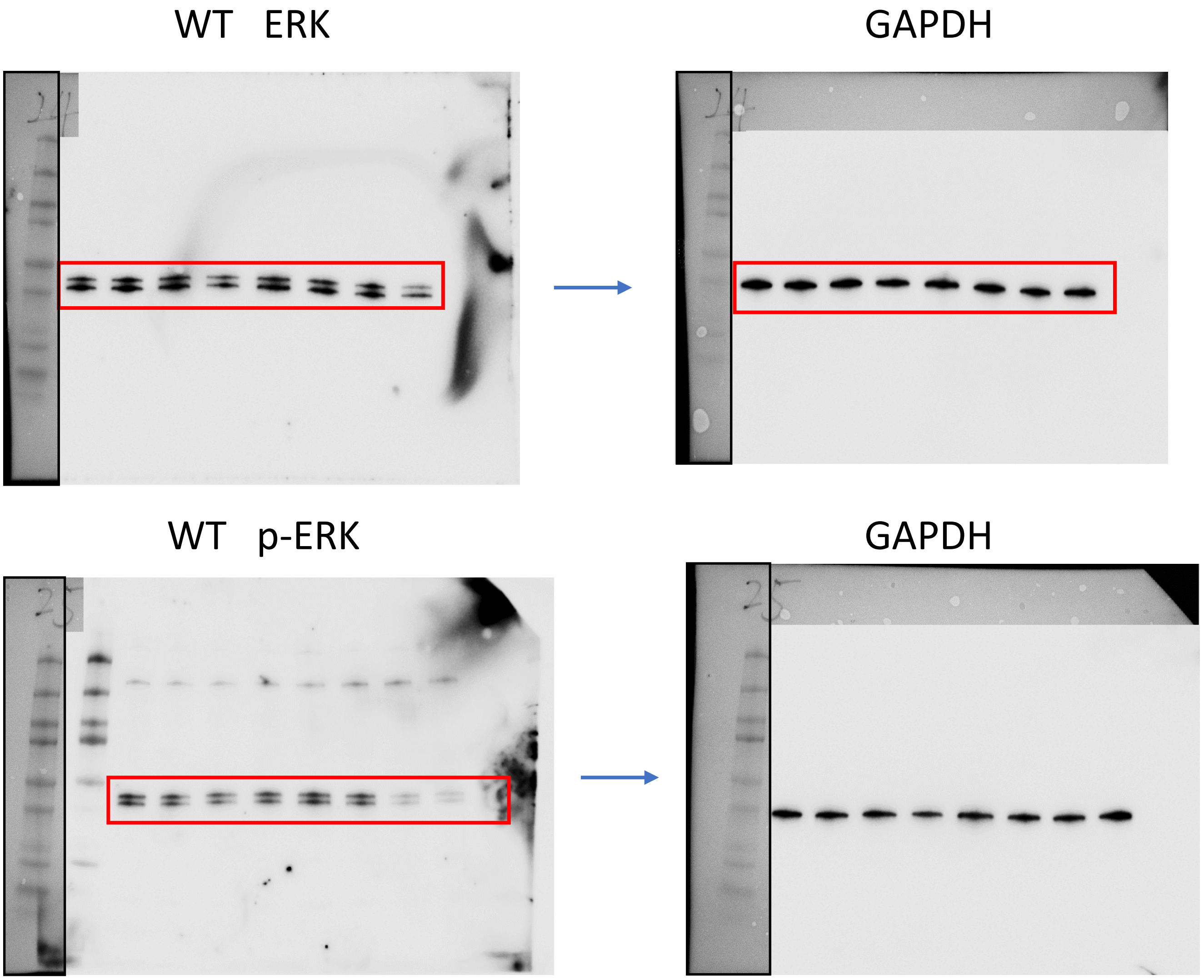

Fig. S32. The full-length images of the original Western blot membranes demonstrating expression of indicated proteins, shown in Supplemental Fig. S8A in a cropped form. The areas of the membranes that were cropped and presented in Supplemental Fig. S8A for each protein are depicted with red rectangles. The image of the molecular weight ladder loaded on the same gel was taken for each original membrane and was juxtaposed with the image of the same membrane after the Western blot processing. The border of the molecular weight ladder image is depicted by the black rectangle. The molecular weights corresponding to specific bands in the ladder are shown in Supplemental Fig. S8A. Re-probing sequence for the same membrane is indicated with blue arrow.

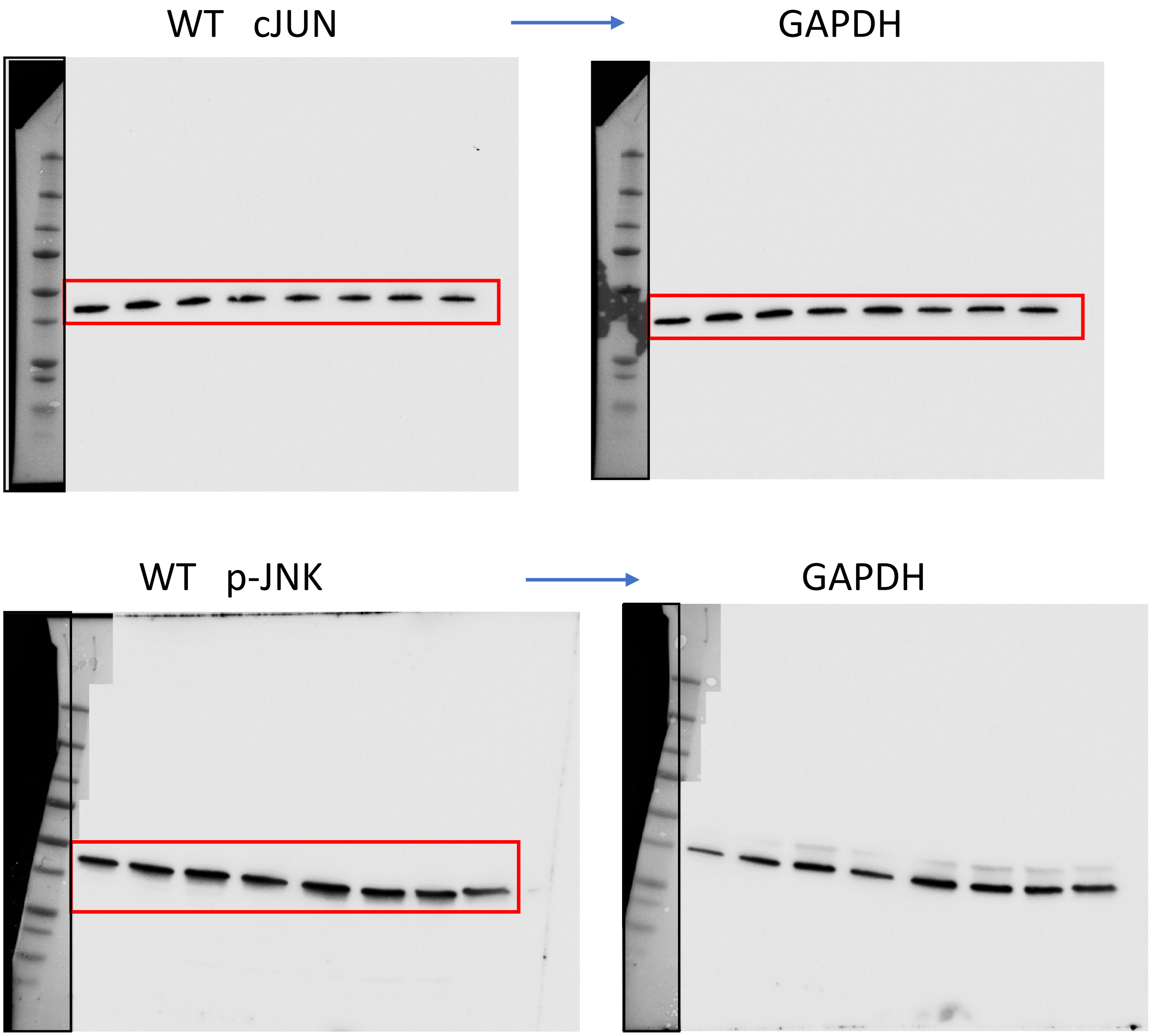

Fig. S33. The full-length images of the original Western blot membranes demonstrating expression of indicated proteins, shown in Supplemental Fig. S8A in a cropped form. The areas of the membranes that were cropped and presented in Supplemental Fig. S8A for each protein are depicted with red rectangles. The image of the molecular weight ladder loaded on the same gel was taken for each original membrane and was juxtaposed with the image of the same membrane after the Western blot processing. The border of the molecular weight ladder image is depicted by the black rectangle. The molecular weights corresponding to specific bands in the ladder are shown in Supplemental Fig. S8A. Re-probing sequence for the same membrane is indicated with blue arrow.

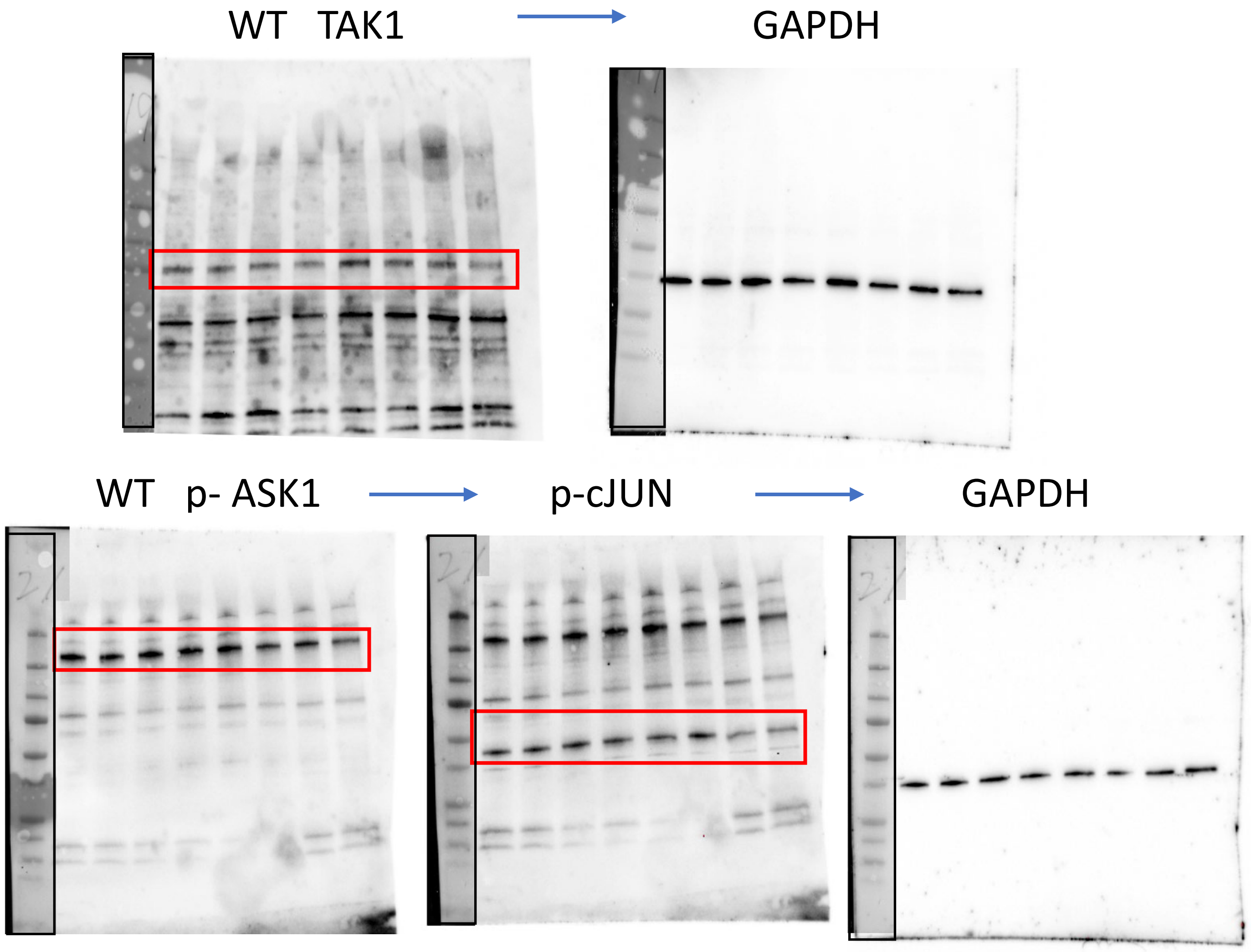

Fig. S34. The full-length images of the original Western blot membranes demonstrating expression of indicated proteins, shown in Supplemental Fig. S8A and Fig. S8C in a cropped form. The areas of the membranes that were cropped and presented in Supplemental Fig. S8A and Fig. 8C for each protein are depicted with red rectangles. The image of the molecular weight ladder loaded on the same gel was taken for each original membrane and was juxtaposed with the image of the same membrane after the Western blot processing. The border of the molecular weight ladder image is depicted by the black rectangle. The molecular weights corresponding to specific bands in the ladder are shown in Supplemental Fig. S8A and Fig. 8C. Re-probing sequence for the same membrane is indicated with blue arrow.

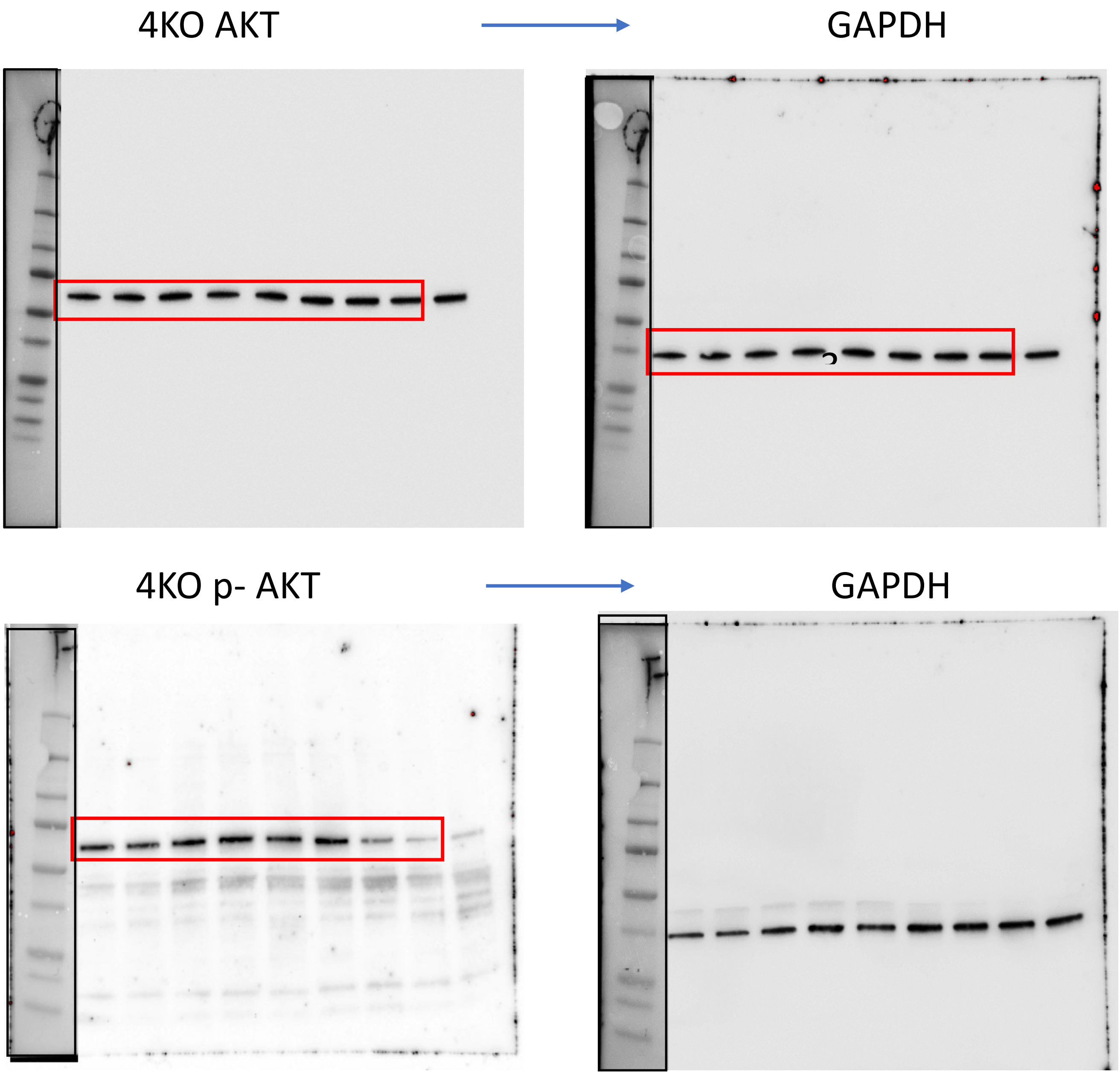

Fig. S35. The full-length images of the original Western blot membranes demonstrating expression of indicated proteins, shown in Supplemental Fig. S8B in a cropped form. The areas of the membranes that were cropped and presented in Supplemental Fig. S8B for each protein are depicted with red rectangles. The image of the molecular weight ladder loaded on the same gel was taken for each original membrane and was juxtaposed with the image of the same membrane after the Western blot processing. The border of the molecular weight ladder image is depicted by the black rectangle. The molecular weights corresponding to specific bands in the ladder are shown in Supplemental Fig. S8B. Re-probing sequence for the same membrane is indicated with blue arrow.

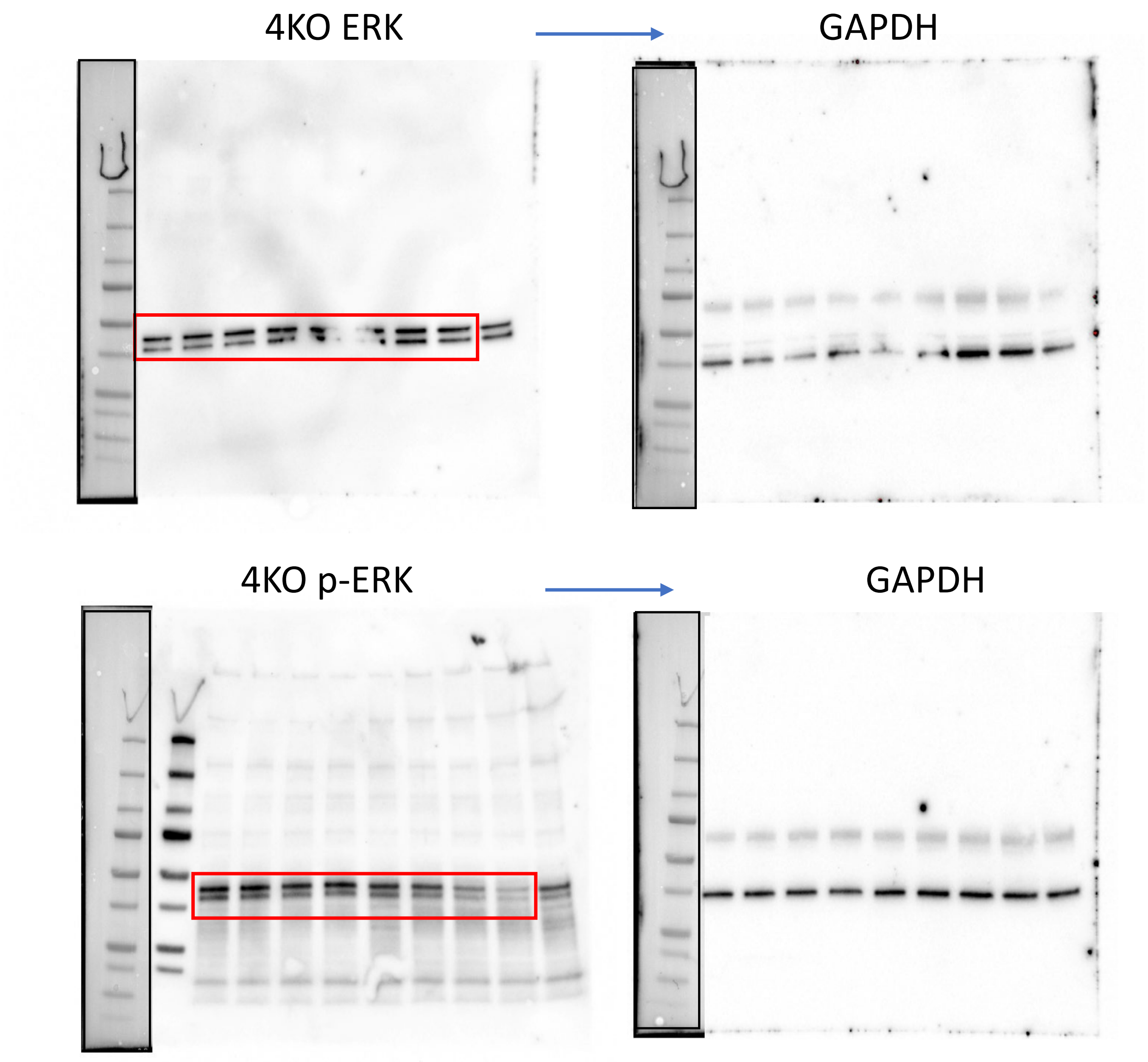

Fig. S36. The full-length images of the original Western blot membranes demonstrating expression of indicated proteins, shown in Supplemental Fig. S8B in a cropped form. The areas of the membranes that were cropped and presented in Supplemental Fig. S8B for each protein are depicted with red rectangles. The image of the molecular weight ladder loaded on the same gel was taken for each original membrane and was juxtaposed with the image of the same membrane after the Western blot processing. The border of the molecular weight ladder image is depicted by the black rectangle. The molecular weights corresponding to specific bands in the ladder are shown in Supplemental Fig. S8B. Re-probing sequence for the same membrane is indicated with blue arrow.

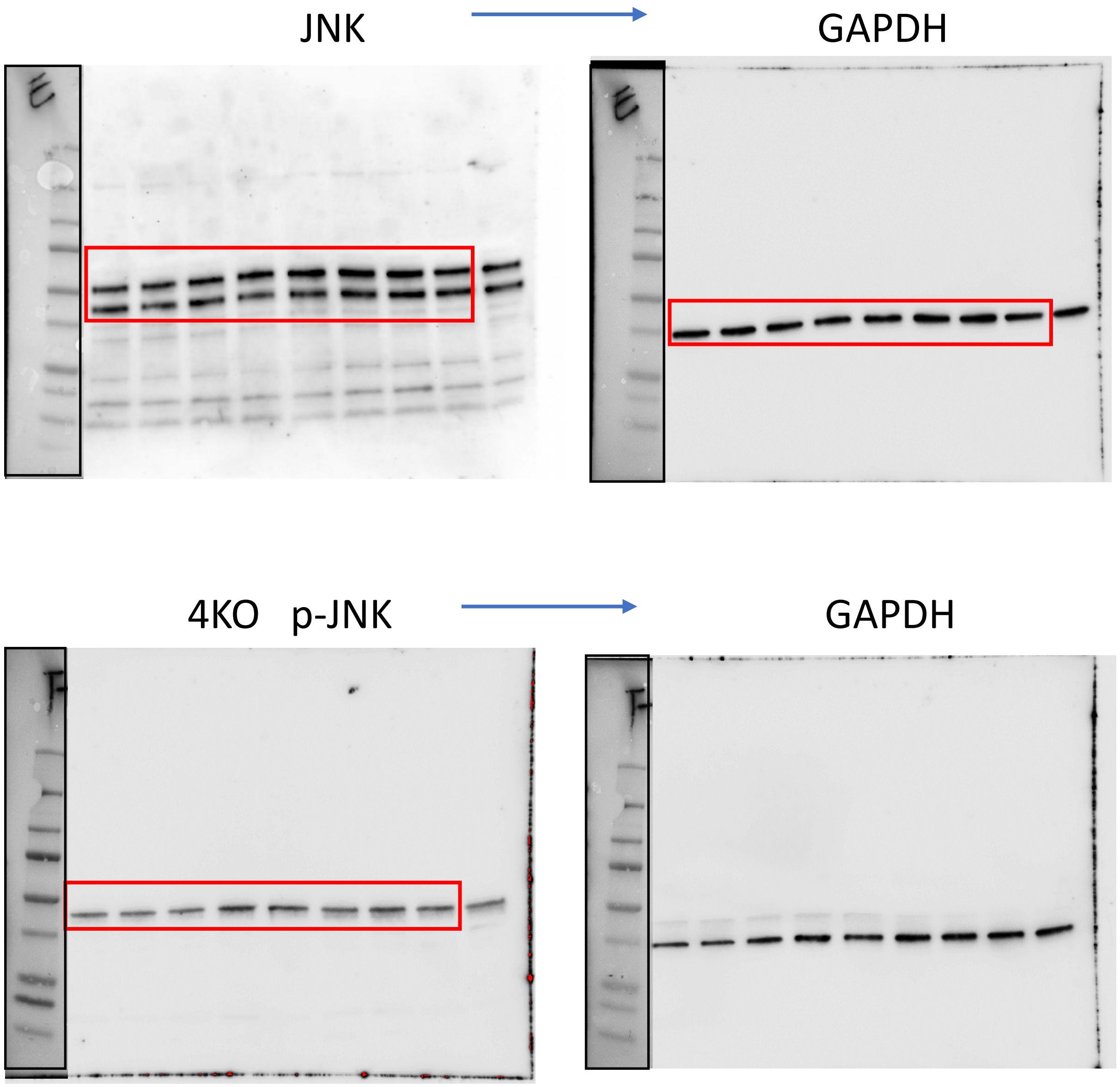

Fig. S37. The full-length images of the original Western blot membranes demonstrating expression of indicated proteins, shown in Supplemental Fig. S8B in a cropped form. The areas of the membranes that were cropped and presented in Supplemental Fig. S8B for each protein are depicted with red rectangles. The image of the molecular weight ladder loaded on the same gel was taken for each original membrane and was juxtaposed with the image of the same membrane after the Western blot processing. The border of the molecular weight ladder image is depicted by the black rectangle. The molecular weights corresponding to specific bands in the ladder are shown in Supplemental Fig. S8B. Re-probing sequence for the same membrane is indicated with blue arrow. The membrane for JNK was also used to analyze expression of ATF2 (Fig. 4A). The probing order was JNK, ATF2, GAPDH. The membrane for p-JNK was also used to analyze expression of p-AKT (S35); the order of processing was p-JNK, p-AKT, GAPDH.

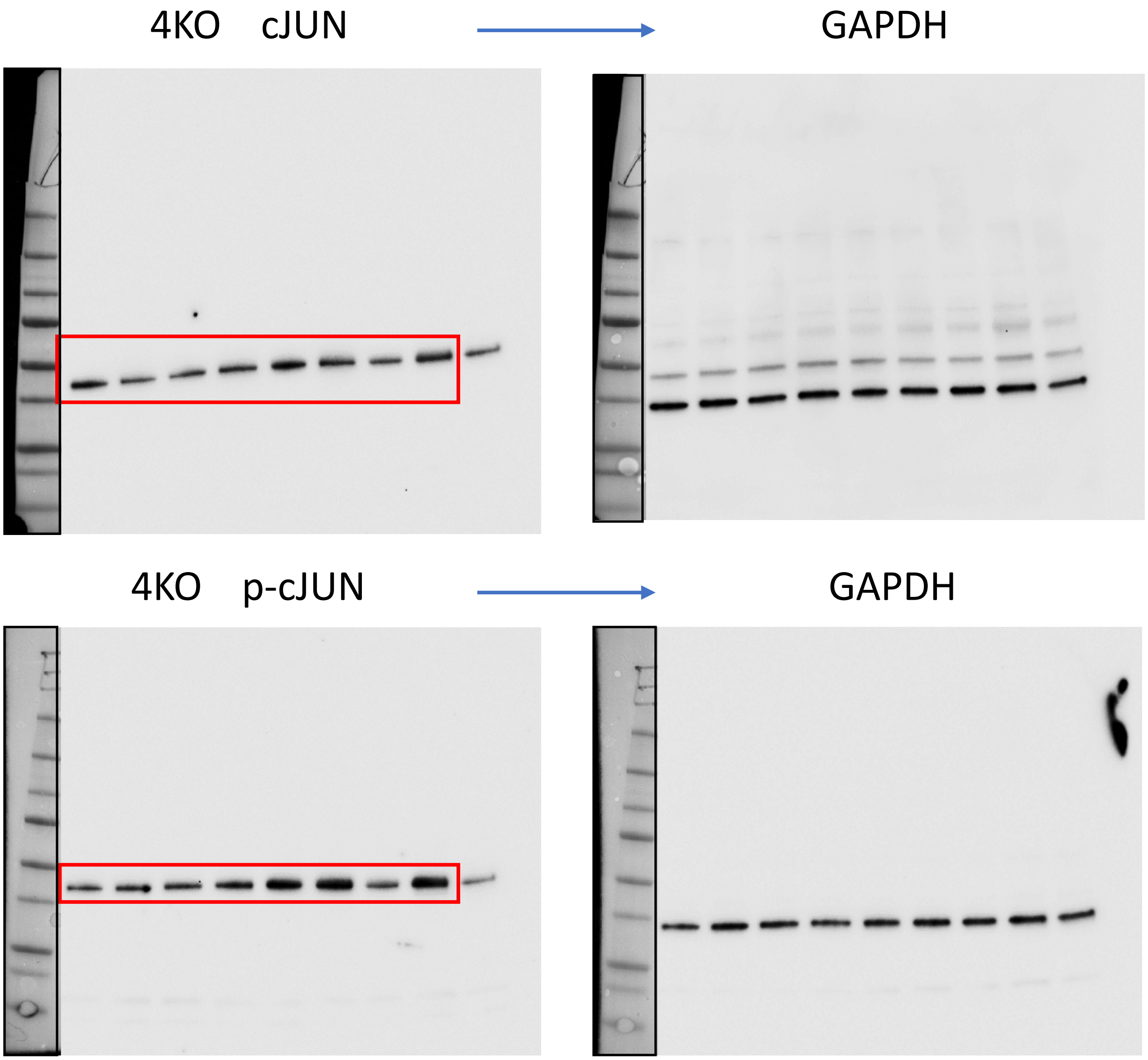

Fig. S38. The full-length images of the original Western blot membranes demonstrating expression of indicated proteins, shown in Supplemental Fig. S8B in a cropped form. The areas of the membranes that were cropped and presented in Supplemental Fig. S8B for each protein are depicted with red rectangles. The image of the molecular weight ladder loaded on the same gel was taken for each original membrane and was juxtaposed with the image of the same membrane after the Western blot processing. The border of the molecular weight ladder image is depicted by the black rectangle. The molecular weights corresponding to specific bands in the ladder are shown in Supplemental Fig. S8B. Re-probing sequence for the same membrane is indicated with blue arrow.

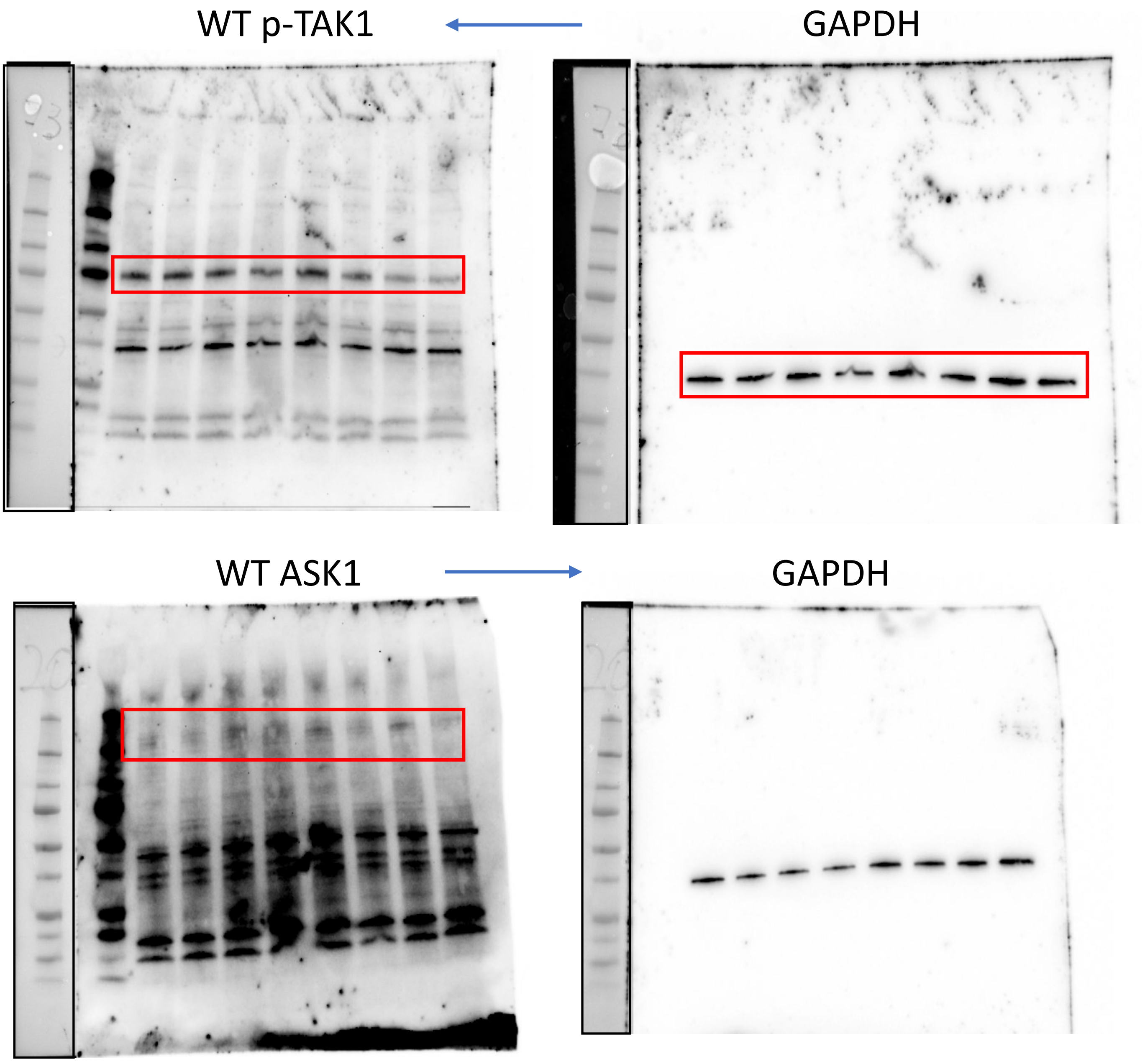

Fig. S39. The full-length images of the original Western blot membranes demonstrating expression of indicated proteins, shown in Supplemental Fig. S8C in a cropped form. The areas of the membranes that were cropped and presented in Supplemental Fig. S8C for each protein are depicted with red rectangles. The image of the molecular weight ladder loaded on the same gel was taken for each original membrane and was juxtaposed with the image of the same membrane after the Western blot processing. The border of the molecular weight ladder image is depicted by the black rectangle. The molecular weights corresponding to specific bands in the ladder are shown in Supplemental Fig. S8C. Re-probing sequence for the same membrane is indicated with blue arrow.

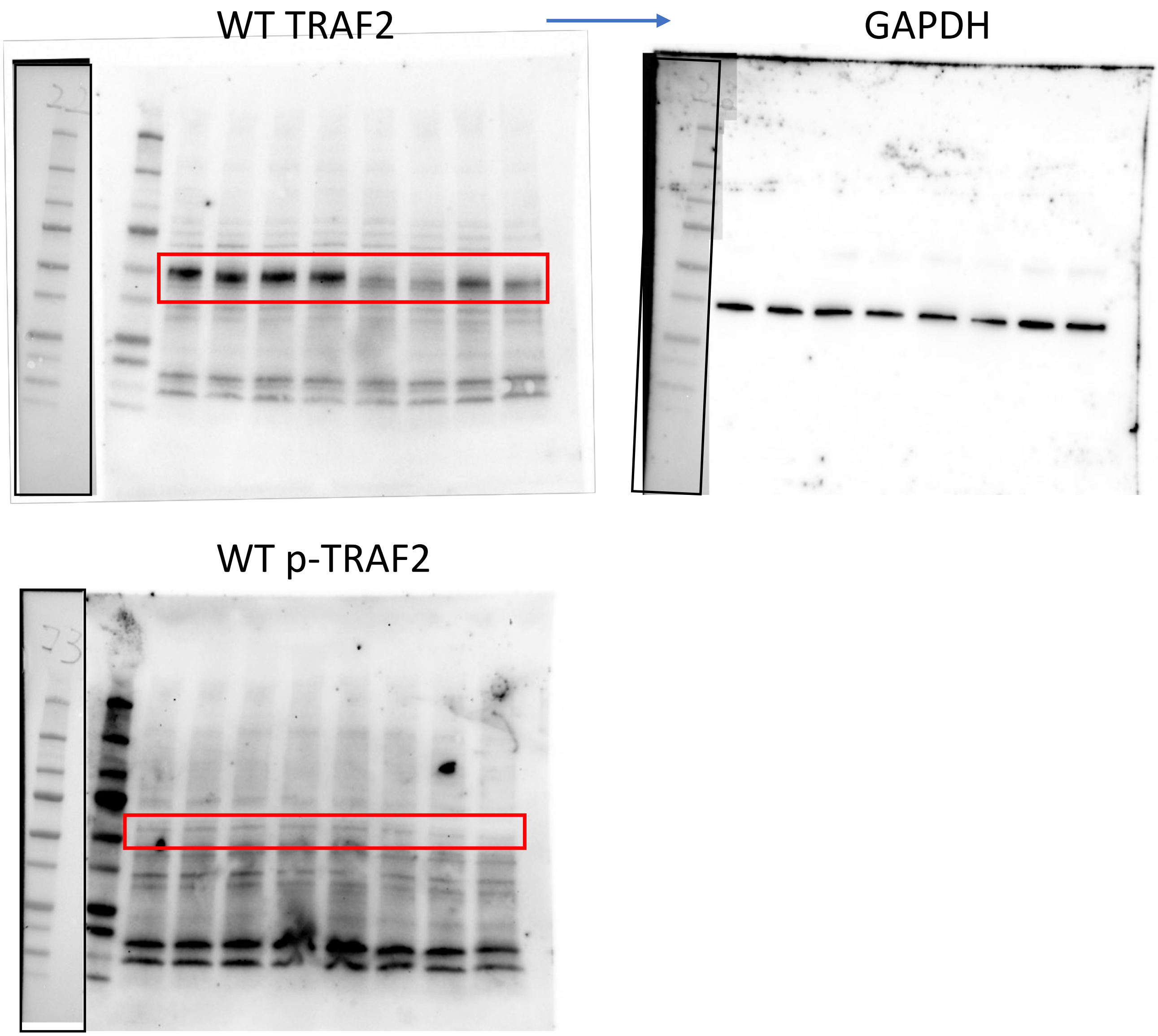

Fig. S40. The full-length images of the original Western blot membranes demonstrating expression of indicated proteins, shown in Supplemental Fig. S8C in a cropped form. The areas of the membranes that were cropped and presented in Supplemental Fig. S8C for each protein are depicted with red rectangles. The image of the molecular weight ladder loaded on the same gel was taken for each original membrane and was juxtaposed with the image of the same membrane after the Western blot processing. The border of the molecular weight ladder image is depicted by the black rectangle. The molecular weights corresponding to specific bands in the ladder are shown in Supplemental Fig. S8C. Re-probing sequence for the same membrane is indicated with blue arrow.

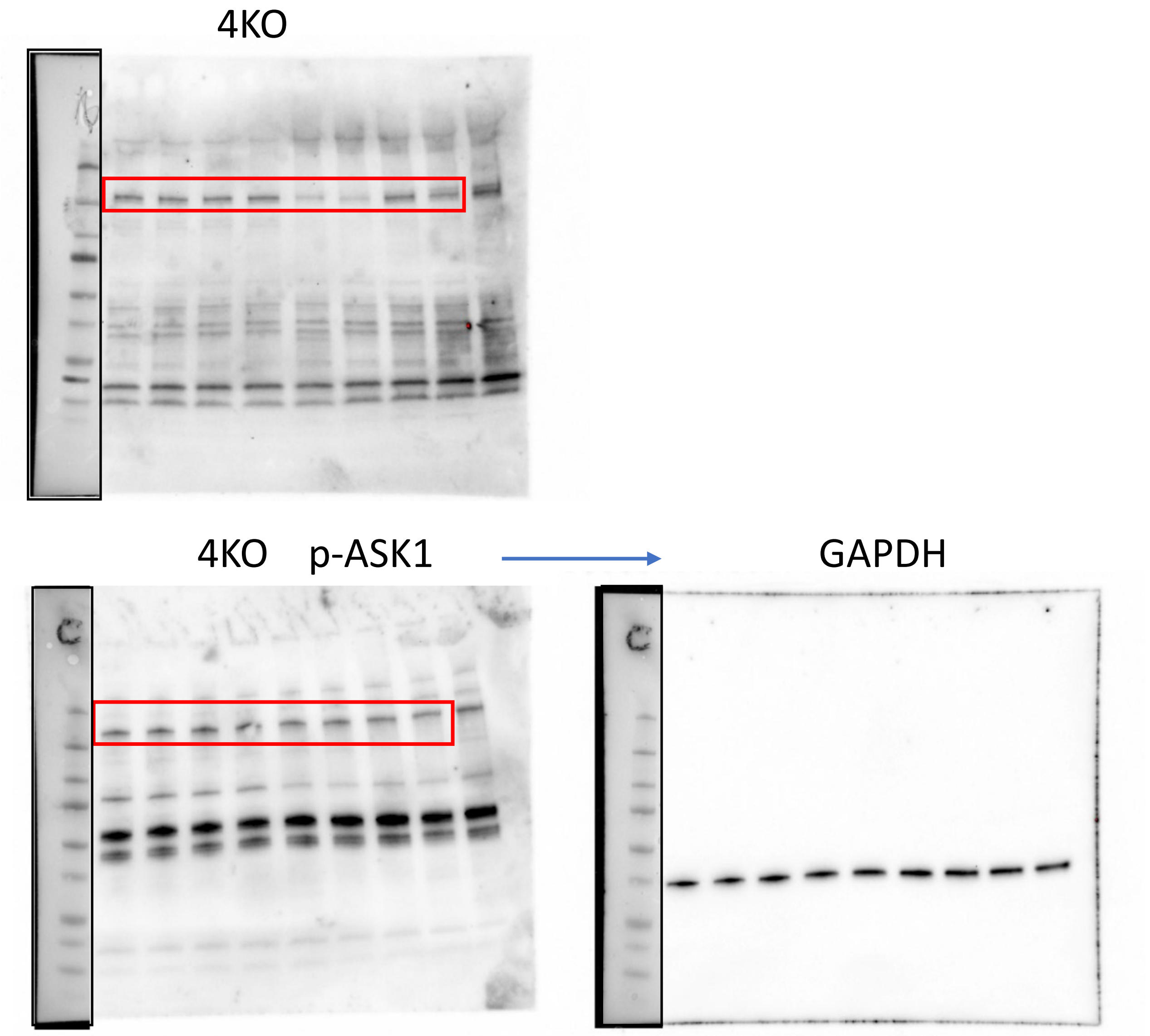

Fig. S41. The full-length images of the original Western blot membranes demonstrating expression of indicated proteins, shown in Supplemental Fig. S8D in a cropped form. The areas of the membranes that were cropped and presented in Supplemental Fig. S8D for each protein are depicted with red rectangles. The image of the molecular weight ladder loaded on the same gel was taken for each original membrane and was juxtaposed with the image of the same membrane after the Western blot processing. The border of the molecular weight ladder image is depicted by the black rectangle. The molecular weights corresponding to specific bands in the ladder are shown in Supplemental Fig. S8D. Re-probing sequence for the same membrane is indicated with blue arrow. The membrane for 4KO p-ASK1 was also used for analyzing MK2 in 4KO cells (Fig4A). The probing order was MK2, pASK1, GAPDH.

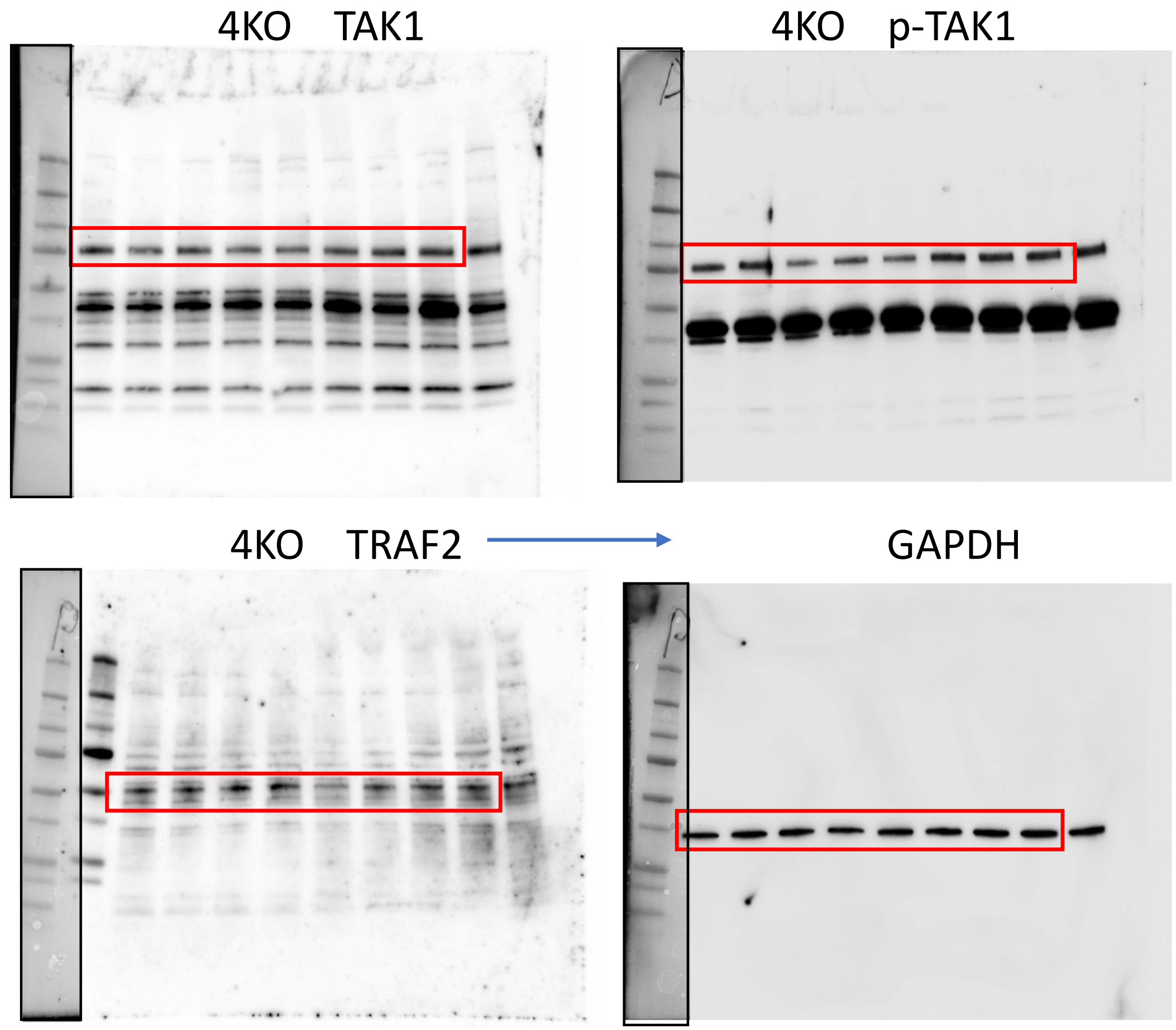

Fig. S42. The full-length images of the original Western blot membranes demonstrating expression of indicated proteins, shown in Supplemental Fig. S8D in a cropped form. The areas of the membranes that were cropped and presented in Supplemental Fig. S8D for each protein are depicted with red rectangles. The image of the molecular weight ladder loaded on the same gel was taken for each original membrane and was juxtaposed with the image of the same membrane after the Western blot processing. The border of the molecular weight ladder image is depicted by the black rectangle. The molecular weights corresponding to specific bands in the ladder are shown in Supplemental Fig. S8D. Re-probing sequence for the same membrane is indicated with blue arrow. The membrane for TAK1 was used to analyze expression of p-p38 first (Fig4A). The membrane for p-TAK1 was used to analyze expression of p38 (Fig4A). The strong low molecular weight bands represent signal post p-p38 and p38 staining, respectively.

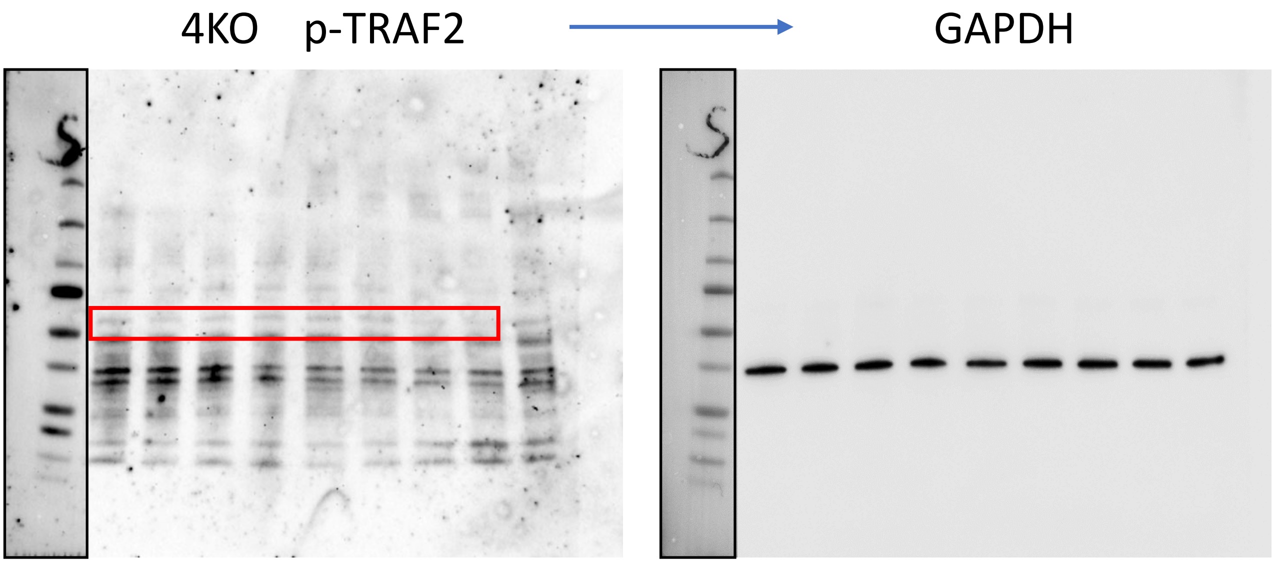

Fig. S43. The full-length images of the original Western blot membranes demonstrating expression of indicated proteins, shown in Supplemental Fig. S8D in a cropped form. The areas of the membranes that were cropped and presented in Supplemental Fig. S8D for each protein are depicted with red rectangles. The image of the molecular weight ladder loaded on the same gel was taken for each original membrane and was juxtaposed with the image of the same membrane after the Western blot processing. The border of the molecular weight ladder image is depicted by the black rectangle. The molecular weights corresponding to specific bands in the ladder are shown in Supplemental Fig. S8D. Re-probing sequence for the same membrane is indicated with blue arrow.

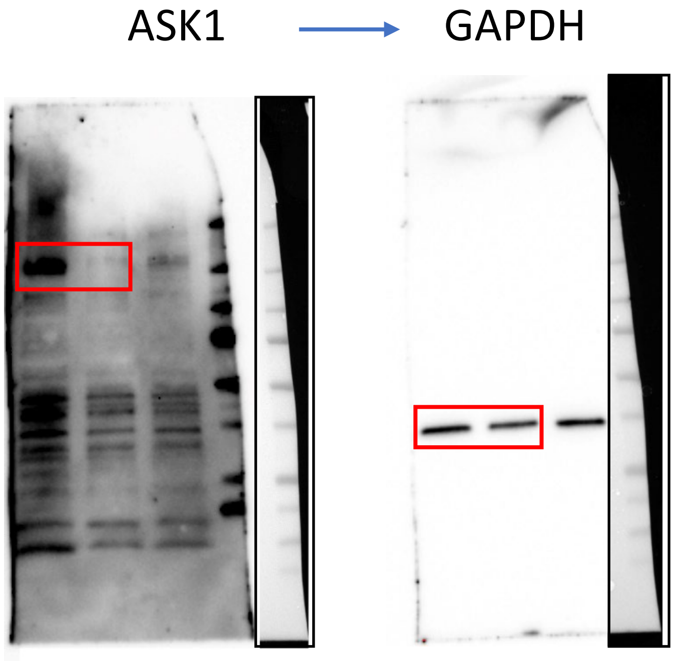

Fig. S44. The full-length images of the original Western blot membranes demonstrating expression of indicated proteins, shown in Supplemental Fig. S8D in a cropped form. The areas of the membranes that were cropped and presented in Supplemental Fig. S8D for each protein are depicted with red rectangles. The image of the molecular weight ladder loaded on the same gel was taken for each original membrane and was juxtaposed with the image of the same membrane after the Western blot processing. The border of the molecular weight ladder image is depicted by the black rectangle. The molecular weights corresponding to specific bands in the ladder are shown in Supplemental Fig. S8D. Re-probing sequence for the same membrane is indicated with blue arrow.

Table S3. List of antibodies used in this study and their providers.

Proteins detected	Cat #	Vendors
Actin	A3853	Sigma
Akt (pan)	#4691	Cell Signaling
phospho-Akt (Ser473)	#4060	Cell Signaling
ASK1	ab45178	abcam
phospho-ASK1 (Thr845)	sc-109911	Santa Cruz
ATF2	#9226	Cell Signaling
phospho- ATF2 (Thr71)	#9221	Cell Signaling
Bak	SAB2500149	Sigma
Bax	ab32503	abcam
Bid	MAB860	R&D
Caspase-1	sc-514	Santa Cruz
Caspase- 3	#9662	Cell Signaling
Caspase -7	ab181579, ab2323	abcam
Caspase- 8	ALX-804-447	Enzo
Caspase- 11	14-9935	affymetrix eBioscience
p44/42 MAPK (Erk1/2)	#9107	Cell Signaling
Phospho-Erk1/2 (Thr202/Tyr204)	#4370	Cell Signaling
GAPDH	MA5-15738	ThermoFisher Scientific
Goat anti-rabbit IgG, HRP	#7074	Cell Signaling
Goat anti-Mouse IgG, HRP	# 62-6520	Invitrogen
Goat Anti-Rat IgG, HRP	ab97057	abcam
JNK	#9252	Cell Signaling
phospho-JNK (Thr183/Tyr185)	sc-6254	Santa Cruz
c-JUN	#9165	Cell Signaling
phospho-c-JUN (Ser73)	#3270	Cell Signaling
MAPKAPK2(MK2)	#12155	Cell Signaling
phospho-MK2 (Thr334)	#3007	Cell Signaling
MLKL	MABC604	Millipore
phospho-MLKL (Ser345)	ab196436	abcam
p38 MAPK	#9212	Cell Signaling
phospho-p38 (Thr180/Tyr182)	#4511	Cell Signaling
Ripk1	ab72139	abcam
Ripk1	#3493	Cell Signaling
Ripk3	#14401, #15828	Cell Signaling
TAK1	#4505	Cell Signaling
phospho-TAK1 (Ser412)	#9339	Cell Signaling
TRAF2	#4724	Cell Signaling
phospho-TRAF2 (Ser11)	#13908	Cell Signaling

Table S4. List of chemicals used in this study and their concentrations.

Chemicals	Targeted proteins/pathways	Final concentration (µm)
BIRB796	pan p38MAPK	10
Cyclosporine A (CsA)	calcineurin, cyclophilin D	1
Deferoxamine mesylate	ferroptosis	100
Ferrostatin-1	ferroptosis	10
GSK843	Ripk3	3
GSK872	Ripk3	3
Liproxstatin-1	ferroptosis	10
N-acetyl-L-cysteine	ROS	3000
Necrostatin-1	Ripk1	20
PF3644022	MK2	10
Z-vad	pan-caspases	25
